# Nickel-Catalyzed Deuteration of Primary, Secondary, and Tertiary Silanes: Scope and Mechanistic Insights

**DOI:** 10.1021/acs.joc.5c00107

**Published:** 2025-04-08

**Authors:** Carlos J. Laglera-Gándara, Rafael Jiménez-Rioboó, Lucía Álvarez-Rodríguez, Riccardo Peloso, Pablo Ríos, Amor Rodríguez

**Affiliations:** Instituto de Investigaciones Químicas, Departamento de Química Inorgánica, 16778 CSIC and Universidad de Sevilla, Centro de Innovación en Química Avanzada (ORFEO−CINQA), C/Américo Vespucio 49, Sevilla 41092, Spain

## Abstract

Deuterated silanes are crucial reagents for deuteration, with a diverse range of applications in materials science, pharmaceuticals, and isotopic labeling. While most methods for synthesizing deuterated silanes rely on stoichiometric environmentally harmful processes or noble metal catalysts, research into more sustainable alternatives has received relatively less attention. In this study, we introduce a catalyst based on a nickel PBP-pincer system (PBP = bis­(phosphino)­boryl), which effectively facilitates catalytic hydrogen/deuterium exchange for primary, secondary, and tertiary silanes, as well as tertiary siloxanes and certain boranes, utilizing a catalyst loading of 2 mol % at 25 °C. DFT calculations identify two reaction pathways that require overcoming similar energy barriers for the H/D exchange step: silane activation assisted by the PBP ligand (Δ*G* ^⧧^ = 24.1 kcal mol^–1^) and H/D exchange promoted by nucleophilic Ni-hydride (Δ*G* ^⧧^ = 22.4 kcal mol^–1^). These results suggest that both pathways are feasible, with a slight energetic preference for the latter. We also present detailed mechanistic studies, including control experiments, an analysis of catalyst deactivation pathways, and kinetic studies that are in excellent agreement with the outcome of the theoretical calculations.

## Graphical abstract

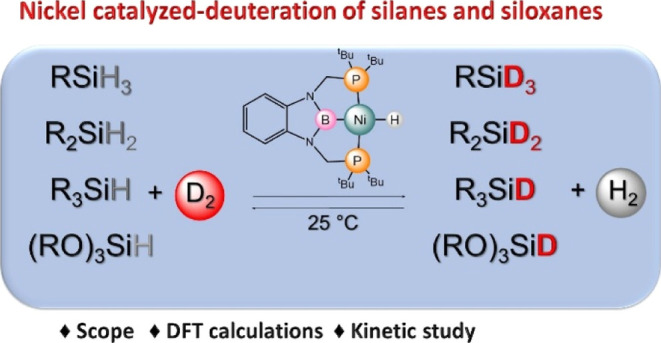

## Introduction

Deuterium-labeled compounds are of great importance due to their usefulness in chemical research and food and pharmaceutical industry.^1^ Replacement of hydrogen for deuterium in an organic entity provides valuable information on reaction mechanisms in biological and chemical processes while maintaining their chemical structure and biological activity virtually unchanged.^2,3^ Additionally, they are also very effective for quantitative analysis and bioanalytical investigations as internal standards for mass spectrometry. In medicinal chemistry, the use of deuterium instead of hydrogen can affect the metabolism of deuterated drugs with beneficial effects on safety, tolerability, and efficacy. The US Food and Drug Administration's approval of the first deuterated drug in 2017 serves as an important stimulus for the development of this field with direct implications in our lives.^4–6^

Deuterated silanes can be used as efficient reagents to incorporate deuterium atoms into organic molecules via addition reactions to unsaturated carbon–carbon and carbon–heteroatom bonds.^7–9^ However, the synthetic protocols associated with their synthesis suffer from environmental problems due to the generation of significant amounts of waste from the stoichiometric reaction of chlorosilanes with metal deuterides such as NaBD_4_ or LiAlD_4_, which makes them unattractive for practical and economic reasons. Recent reports have highlighted interesting approaches utilizing organo photocatalysts using D_2_O as a deuterium source.^10,11^ Alternatively, catalysts for H/D exchange of silanes based on precious metals (Rh,^12–14^ Ir,^15,16^ Ru,^17^ and Pt^18^) have successfully been applied in recent years. Although most of these systems are restricted to the use of tertiary silanes, rhodium and platinum catalysts reported by the groups of Carmona^12^ and Apeloig^18^ were shown to be active also for primary and secondary silanes. Moreover, the associated advantages of low cost and wide availability of 3d metals have motivated research in this area.^19–21^ The inclusion of first-row transition metals in this family of catalysts came from Webster's group with the iron β-diketiminato complex **1** (Chart 1).^19^ Primary and secondary silanes, both aliphatic and aromatic, as well as tertiary siloxanes were deuterated; however, deuteration of tertiary silanes was not observed. Subsequently, Tamm and co-workers reported an amido-imidazolidin-2-imine cobalt complex, **2**,^20^ for the deuteration of a wide variety of silanes and extended this work to analogous iron catalysts **3** and **4** (Chart 1).^21^ To date, precedents for nickel-catalyzed H/D exchange of silanes with D_2_ are limited to two examples: one contribution by Peters and co-workers, in which only one silane (Ph_2_SiH_2_) was tested,^22^ and the other by Radius and co-workers, who described the deuteration of Et_3_SiH but using C_6_D_6_ as the deuterium source.^23^

**Chart 1:**
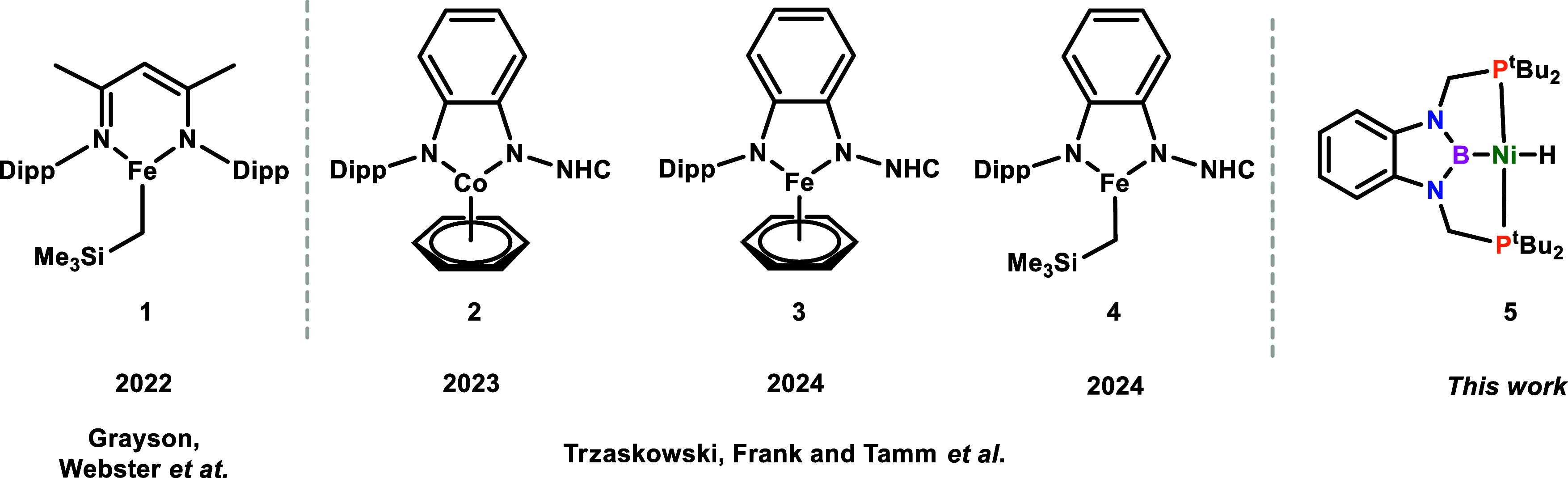
Reported Catalysts for the H/D Exchange of Silanes with D_2_ Based on Earth-Abundant Transition Metals[Fn c1fn1]

a Dipp: 2,6-^*i*^Pr_2_C_6_H_4_; NHC: C­(N^*i*^PrCMe)_2_.

Research in our group over the past few years has focused on bis­(phosphino)­boryl nickel pincer (PBP) systems that promote metal–ligand cooperativity for different chemical transformations.^24–27^ Highly selective catalytic hydrosilylation of carbon dioxide to bis­(silyl)­acetal,^24,25^ reduction of aldehydes and ketones,^26^ and oligomerization of ethylene have been achieved.^27^ Our efforts have been dedicated to shed light on the mechanisms involved in these reactions, probing a relevant role of the boryl group in the activation processes. The strong sigma-donor properties of the sp^2^ boron center along with the presence of an empty p orbital allow for an ambiphilic reactivity that reveals unexpected reaction pathways.^28^

Inspired by the recent work on first-row transition metals for deuteration of silanes^19–23^ and considering the scarcity of nickel-based compounds capable to catalyze this transformation, we decided to test the complex (^*t*Bu^PBP)­NiH (**5**) in the catalytic H/D exchange of hydrosilanes and some hydroboranes (Chart 1).^29^

## Results

### Catalytic Studies

We began our studies with the secondary silane Et_2_SiH_2_ as a model substrate, using 5 mol % catalyst loading at room temperature under 2 bar of deuterium gas, in a J. Young valve NMR tube. The 72% deuterium incorporation was measured by ^1^H NMR by integration of the residual Si–H resonance and the ethyl signals after 19 h (Table 1, entry 1). By increasing the D_2_ pressure from 2 to 4 bar, a conversion of 83% was achieved (Table 1, entry 2). Under these reaction conditions (4 bar D_2_, 25 °C, 19 h) but with lower catalyst loading (2 mol % of Ni), a conversion of 80% was obtained (Table 1, entry 3).

**Table 1:** Initial Attempts for Deuteration of Diethylsilane^a^

entry	catalyst	loading (mol %)	time (h)	P_D2_ (bar)	yield (%)^b^
1	**5**	5	19	2	72
2	**5**	5	19	4	83
3	**5**	2	19	4	80
4^c^	**5**	2	19	4	85
5^d^	**5**	2	4	2	99
6	^*t*Bu^PBPH	5	19	2	0
7	(DME)NiBr_2_	5	19	2	0
8^e^	**5**	5	19	0	0

a Reaction conditions: (^*t*Bu^PBP)­NiH (0.004 mmol); 19 h; using C_6_D_6_ as the solvent (400 μL for each reaction); the reactions are performed at 25 °C in a J. Young NMR tube.

b Conversion determined by ^1^H NMR.

c After 19 h, the tube was frozen and evacuated under vacuum, after which the headspace was filled with deuterium gas (4 bar).

d The reaction is performed in a 20 mL Fischer–Porter tube.

e C_6_D_6_ was the only deuterium source.

Evacuation of the headspace of the NMR tube and replenishment of the deuterium atmosphere did not significantly increase the deuterium incorporation (Table 1, entry 4). Increasing the volume of the reaction vessel (from a 1.5 mL volume NMR tube to a 20 mL Fischer–Porter tube) allows for higher conversion and shorter reaction times (99% incorporation of deuterium, 4 h, 2 bar D_2_, 25 °C) (Table 1, entry 5). Control experiments showed no reaction in the absence of **5**, confirming that the deuteration was indeed catalyzed by the nickel hydride complex. Control experiments using the ^*t*Bu^PBP ligand or nickel precursor (DME)­NiBr_2_ as potential catalysts or using C_6_D_6_ as the only deuterium source showed no activity for the deuteration of Et_2_SiH_2_ (Table 1, entries 6–8). With the optimal conditions in hand, we set out to explore the substrate scope (Table 2). Catalytic deuteration of other aliphatic (PhMeSiH_2_, **1c**) and aromatic (Ph_2_SiH_2_, **1b**) secondary silanes, under optimized conditions, gives 94% and 95% deuterium incorporation, respectively (Table 2, entries 2 and 3). With a bulkier silane such as ^*t*^Bu_2_SiH_2_ (**1d**), the reaction proved to be more difficult, reaching only 54% deuterium incorporation (Table 2, entry 4). Large-scale synthesis of Et_2_SiD_2_ (**2a**) could be performed in high yield with 98% incorporation of deuterium (Table 2, entry 5). H/D exchange with tertiary silanes Ph_3_SiH (**1e**), Et_3_SiH (**1f**), Ph_2_MeSiH (**1g**), and PhMe_2_SiH (**1h**) takes place with conversions of 94 to 97% (Table 2, entries 6–9). (EtO)_3_SiH (**1i**), (MeO)_3_SiH (**1j**), and (MeO)_2_MeSiH (**1k**) were also deuterated and a small amount of disilane formation (from 5 to 6%) was detected (Table 2, entries 10 and 12). When primary *n*-hexylsilane (**1l**) was used, fully deuterated silane **2l** was formed in 98% spectroscopic yield (Table 2, entry 13). On the contrary, deuteration of PhSiH_3_ (**1m**) also exhibited partial decomposition of the catalyst (Table 2, entry 14).^30^ No deuteration was observed when polymethylhydrosiloxane (PMHS) (**1n**) or ClMe_2_SiH (**1o**) was used (entries 15 and 16). In the former case, **5** is recovered intact, but in the presence of **1o**, catalyst **5** is transformed into inactive species (^*t*Bu^PBP)­NiCl (**6**).^31^

**Table 2:** Summary of the Results of H/D Exchanges of Silanes and Boranes Using **5** as a Catalyst^a^

entry	substrate	product	yield (%)^b^
**1**	Et_2_SiH_2_ (**1a**)	Et_2_SiD_2_ (**2a**)	99
**2**	Ph_2_SiH_2_ (**1b**)	Ph_2_SiD_2_ (**2b**)	95
**3**	PhMeSiH_2_ (**1c**)	PhMeSiD_2_ (**2c**)	94
**4**	^*t*^Bu_2_SiH_2_ (**1d**)	^*t*^Bu_2_SiD_2_ (**2d**)	54
**5**	Et_2_SiH_2_ (**1a**)	Et_2_SiD_2_ (**2a**)	98 (60)^c^
**6**	Ph_3_SiH (**1e**)	Ph_3_SiD (**2e**)	94
**7**	Et_3_SiH (**1f**)	Et_3_SiD (**2f**)	96
**8**	Ph_2_MeSiH (**1g**)	Ph_2_MeSiD (**2g**)	96
**9**	PhMe_2_SiH (**1h**)	PhMe_2_SiD (**2h**)	97
**10**	(EtO)_3_SiH (**1i**)	(EtO)_3_SiD (**2i**) (EtO)_6_Si_2_ (**2i′**)	91/5
**11**	(MeO)_3_SiH (**1j**)	(MeO)_3_SiD (**2j**) (MeO)_6_Si_2_ (**2j′**)	91/6
**12**	(MeO)_2_MeSiH (**1k**)	(MeO)_2_MeSiD (**2k**)	96
**13** ^d^	^*n*^HexSiH_3_ (**1l**)	^*n*^HexSiD_3_ (**2l**) ^*n*^HexSiD_2_H (**2l′**) ^*n*^HexSiDH_2_ (**2l″**)	98 < 1 < 1
**14** ^e^	PhSiH_3_ (**1m**)	PhSiD_3_ (**2m**) PhSiD_2_H (**2m′**) PhSiDH_2_ (**2m’’**)	57/2 /< 1
**15** ^f^	PMHS (**1n**)		0
**16**	ClMe_2_SiH (**1o**)		0
**17** ^g^	HBpin (**1p**)	DBpin (**2p**)	99
**18** ^g^	H–B(C_6_H_6_N_2_) (**1q**)	D-B(C_6_H_6_N_2_) (**2q**)	99
**19**	HBcat (**1r**)		0
**20**	9-BBN (**1s**)		0

a Reaction conditions: **5** (0.004 mmol), 4 h, C_6_D_6_, 25 °C, 2 bar D_2_; the reactions are performed in a 20 mL Fischer–Porter tube.

b Deuterium incorporation determined by ^1^H NMR spectroscopy using HMDSO as the internal standard.

c Reaction performed in 1 mmol scale, isolated yield 60%.

d The reaction time was 6 h.

e Catalyst decomposition was observed.

f PMHS: polymethylhydrosiloxane.

g H–B signal was not detected by ^1^H NMR spectroscopy, and spectroscopic yield determined by ^2^H NMR spectroscopy relative to toluene-*d*_8_.

Encouraged by these excellent results with silanes, we wondered whether boranes could also be deuterated. Deuterated boranes hold significant potential as highly effective deuterium sources and can be converted into a wide array of isotopically labeled compounds, capitalizing on the versatile synthetic applications of organoborons. Various transition metal catalysts, including noble metals (Ru, Rh, and Ir)^14,32–34^ as well as first-row transition metals (Fe and Co),^35,36^ have been reported for the deuteration of boranes and diboranes. To evaluate the catalytic performance of **5**, we conducted reactions with various boranes under standard conditions (2 bar D_2_, 25 °C, 4 h, C_6_D_6_). H/D exchange was accomplished for pinacolborane (**1p**) and diaminoborane **1q** (with 99% deuterium incorporation); however, extension to other commercially available boranes such as catecholborane (**1r**) and 9-borabicyclo (3.3.1) nonane (**1s**) failed (Table 2, entries 17–20). Previous work in our group on dihydrogen activation by bis-boryl nickel species (^*t*Bu^PBP)­NiBcat (**7**) proved the formation of a nickel dihydro–borate complex (^*t*Bu^PBP)­NiH_2_Bcat (**8**) after heterolytic activation of H_2_ by the B–Ni–B unit of **7** (Scheme 1).^28^

**Scheme 1:**
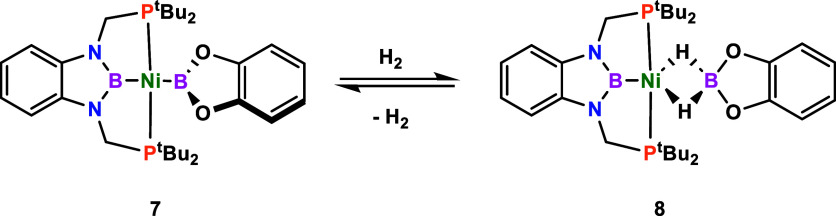
Dihydrogen Activation by **7** to Form Dihydro–Borate Complex **8** ^a^

a Reproduced from ref 28. Available under a CC-BY 3.0 license. Copyright © 2021 Royal Society of Chemistry.

We hypothesize that **8** is formed during the catalytic reaction and may be responsible for the inhibition of catalysis. Consistent with this, when we perform the H/D exchange reaction between HBcat and D_2_, under standard conditions and using complex **8** as a catalyst, deuterium incorporation does not occur. Indeed, computational studies indicate that the formation of **8** is thermodynamically favorable (−3.4 kcal mol^–1^), whereas the Bpin derivative (^*t*Bu^PBP)­NiH_2_Bpin is 1.2 kcal mol^–1^ above the reagents. This energy difference is likely sufficient to prevent borane dissociation necessary for H/D exchange at 25 °C. To overcome this energy barrier, we performed the reaction at 50 °C; however, catalyst decomposition occurred, and deuteration was not observed.

### Computational Investigation and Mechanistic Studies

After analyzing the catalytic activity of our system, we shifted our efforts to the understanding of the reaction mechanism and conducted kinetic studies to clarify how the reaction rate depends on each component involved. Preliminary qualitative observations indicated that the deuteration rate was significantly influenced by the initial concentration of the silane, the pressure of D_2_, and the catalyst loading. The time dependence of the conversion of PhMeSiH_2_ into PhMeSiD_2_ in C_6_D_6_ under an initial pressure of 2 atm of D_2_ was studied by ^1^H NMR spectroscopy, aiming to disclose the rate law of the catalytic deuteration. The results obtained from these experiments are in good agreement with a first-order rate dependence on both the silane and D_2_, i.e., with an overall reaction order of 2 according to the following equation: $-\frac{d\left[{PhMeSiH}_{2}\right]}{dt}=k_{obs}\left(T,\left[5\right]\right)\cdot \left[PhMe{SiH}_{2}\right]\cdot \left[D_{2}\right]$ , in which the rate constant, *k*_obs_, depends on the temperature (*T*) and the catalyst concentration. Additionally, the catalyst loading was varied from 6 to 14% (mol/mol, relative to silane) to calculate the corresponding values of the observed rate constant (*k*_obs_) at 298 K and determine the reaction order with respect to complex **5**. As shown in Figure 1, a linear dependence of *k*_obs_ on the catalyst concentration, [**5**], was found, which is in agreement with a corresponding partial reaction order of 1 (see the Supporting Information for details). Additionally, the catalyst order was also visually confirmed through Variable Time Normalization Analysis (VTNA). The concentration plots obtained by ^1^H NMR display the concentration of PhMeSiH_2_ plotted against a normalized time scale, showing zeroth-, 0.5th-, first-, and second-order dependencies on the initial catalyst concentration. This analysis reveals that the best overlapping curve corresponds to a first-order reaction with respect to the catalyst (see Figure S30).

**Figure 1:**
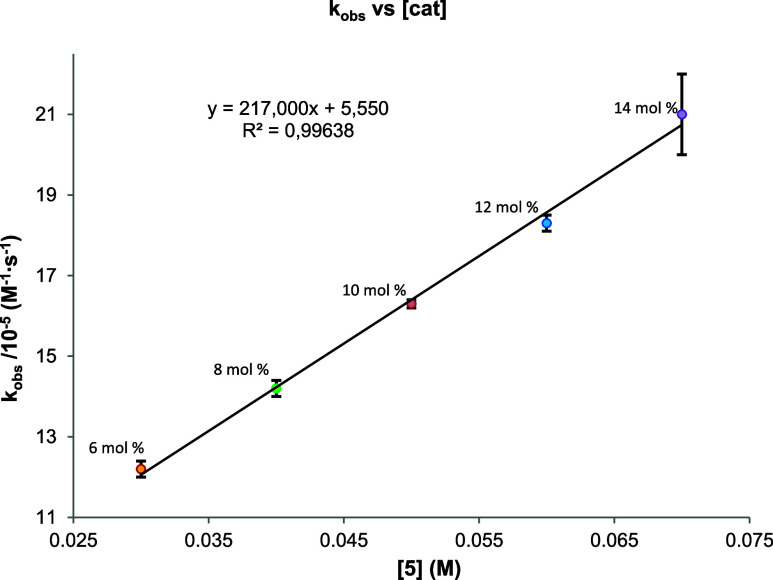
Deuteration of PhMeSiH_2_. Variation of *k*_obs_ vs [**5**].

Last, to estimate experimentally the thermodynamic parameters of the overall energy barrier (Δ*H* ^⧧^, Δ*S* ^⧧^, and Δ*G* ^⧧^), the reaction was monitored by ^1^H NMR spectroscopy at 15, 25, and 35 °C (see the Supporting Information). The representation from the Eyring equation of ln­(*k*_obs_/*T*) versus *1*/*T* provided activation enthalpy (Δ*H* ^⧧^) and entropy (Δ*S* ^⧧^) values of 5.25 ± 0.04 kcal·mol^–1^ and –50.2 ± 0.6 cal·mol^–1^·K^–1^, respectively, with a corresponding free energy of activation (Δ*G* ^⧧^) at 298 K of 20.2 ± 0.3 kcal·mol^–1^ (Figure 2).

**Figure 2:**
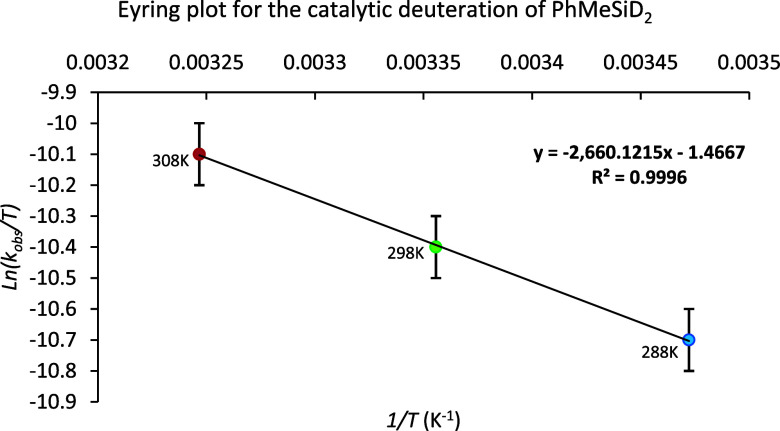
Deuteration of PhMeSiH_2_. Variation of ln­(*k*_obs_/*T*) vs 1/*T*.

The mechanism of the deuteration of silanes catalyzed by **5** was investigated by means of DFT methods using **5-D** as the catalyst and PhMeSiH_2_ as the substrate (preliminary calculations using PhSiH_3_ can also be found in the Supporting Information).^37^ For this process, several mechanistic scenarios were considered, namely, (a) phosphine decoordination, (b) nucleophilic attack on Si, and (c) metal–ligand cooperativity, with active participation of the PBP scaffold. Similar to previous examples,^28^ exploratory calculations revealed that decoordination of one of the phosphine groups from the metal coordination sphere is too energy demanding (36.3 kcal·mol^–1^) to be compatible with the experimental observations (Figure S38). Alternatively, the hydricity of the deuteride ligand due to the strong trans influence exerted by the boryl moiety combined with the hypervalency of Si could lead to H/D exchange on silicon, similar to that reported by Grayson and Webster^19^ or Trzaskowski, Frank, and Tamm.^21^ The results of these calculations are displayed in Figure 3.

**Figure 3:**
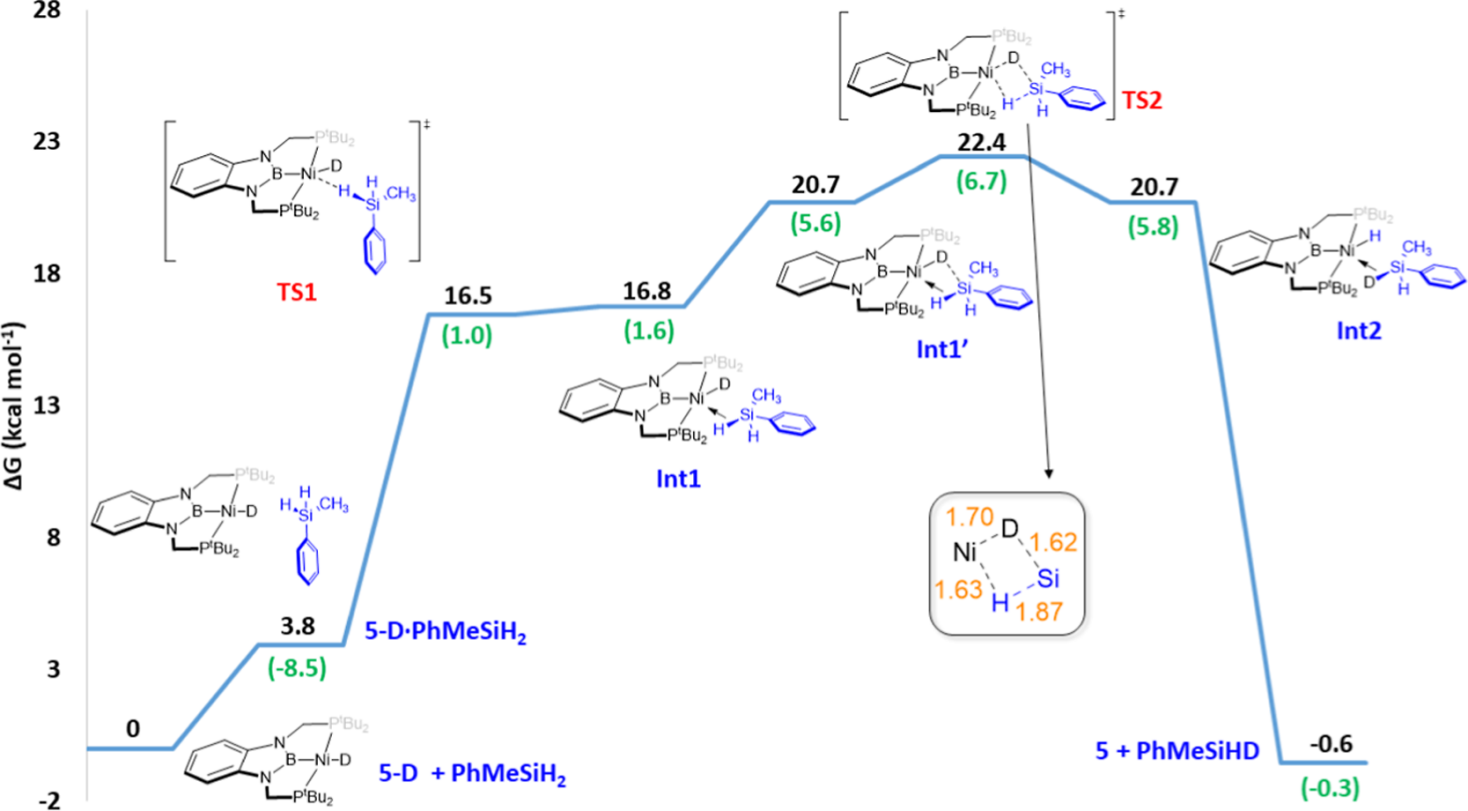
Computed Gibbs energy profile in benzene (SMD) for the H/D exchange in PhMeSiH_2_ mediated by complex **5-D** (nucleophilic attack on Si). Relative Gibbs energies computed at 298 K and 1 M are given in kcal·mol^–1^. The Gibbs energy of **5-D** + PhMeSiH_2_ has been taken as zero energy. Enthalpy values (at 298 K and 1 atm) are highlighted in green. All data have been computed at the SMD-M06L-D3/def2-TZVP/def2-QZVP&SDD//SMD-M06L-D3/6-31G­(d,p)&SDD­(+f) level.

Starting from **5-D** and PhMeSiH_2_, the first step consists of the silane approach to the nickel complex, which takes place via **TS1**. This barrier requires 16.5 kcal·mol^–1^, and it leads to the σ-Si–H complex **Int1**, where the Ni···H and Ni···Si distances (1.69 and 2.51 Å, respectively) are slightly longer than the sum of their covalent radii (1.55 and 2.35 Å, respectively).^38^ Upon coordination, the Si–H bond undergoes considerable weakening, as judged by the elongation from 1.49 (free silane) to 1.61 Å in **Int1**. This intermediate is thermodynamically unstable (16.8 kcal·mol^–1^ above the energy reference) since the Si–H bond is interacting with a filled d_*z*_^2^ orbital. Indeed, an even higher in energy intermediate **Int1′** (20.7 kcal·mol^–1^) was located in the potential energy surface, which exhibits rather similar Ni···H and Ni···Si distances (1.71 and 2.47 Å) but a shorter Si···D distance (1.72 Å vs 1.97 Å in **Int1**). This subtle geometry rearrangement preorganizes the structure for the H/D exchange mediated by Ni, which occurs in a concerted manner through the 4-membered transition state **TS2** (22.4 kcal·mol^–1^). The bond distances shown in Figure 3 (inset) suggest that **TS2** is a late transition state, as the outcome of this step is the σ-Si–D complex **Int2**, analogous to **Int1**. Simple decoordination gives the deuterated silane, which can undergo subsequent H/D exchange steps in order to reach full deuteration.

On the other hand, previous examples from our laboratory have demonstrated the critical role of the boryl moiety of the PBP scaffold in the activation of small molecules such as H_2_. Therefore, a mechanistic scenario involving silane activation assisted by the PBP ligand was investigated, as depicted in Figure 4. Starting from **5-D** and PhMeSiH_2_, the first approach involves again a σ-Si–H complex, namely, **Int3** (18.0 kcal·mol^–1^), which possesses almost identical Ni···Si, Ni···H, and Si–H distances to those observed for complex **Int1**. From this point, the metal center mediates the Si–H bond cleavage and subsequent hydrogen migration to the boron atom of the PBP ligand in transition state **TS3**. This process, which is the rate-determining step of the mechanism, requires 24.1 kcal·mol^–1^ and entails the simultaneous formation of B–H and Si–D bonds. This transformation gives species **Int4** (10.0 kcal·mol^–1^), which consists of a Ni(0) bis σ complex, similar to those previously calculated for nickel­(II) bis­(boryl) compounds.^28^ From this point, silane decoordination followed by oxidative addition of the B–H bond would give **5** and PhMeSiHD, similar to the last step in Figure 3. Nevertheless, exploratory calculations using a smaller basis set revealed that the oxidative addition step in a tricoordinate Ni(0) complex is exceedingly high in energy (33.7 kcal·mol^–1^).

**Figure 4:**
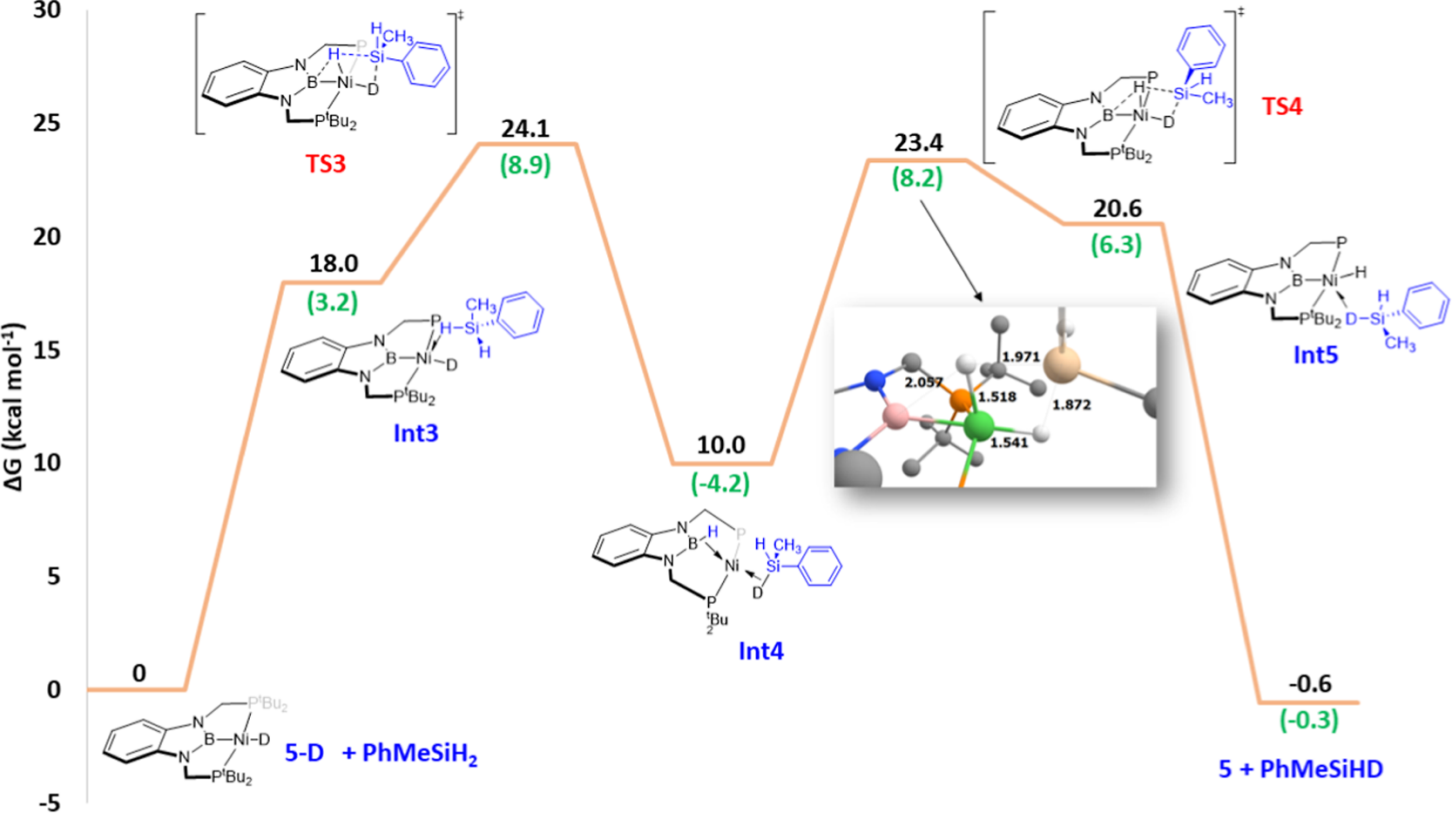
Computed Gibbs energy profile in benzene (SMD) for the H/D exchange in PhMeSiH_2_ mediated by complex **5-D** (metal–ligand cooperativity). Relative Gibbs energies computed at 298 K and 1 M are given in kcal·mol^–1^. The Gibbs energy of **5-D** + PhMeSiH_2_ has been taken as zero energy. Enthalpy values (at 298 K and 1 atm) are highlighted in green. All data have been computed at the SMD-M06L-D3/def2-TZVP/def2-QZVP&SDD//SMD-M06L-D3/6-31G­(d,p)&SDD­(+f) level.

Interestingly, this step can be assisted by the bound deuterated silane (**TS4**), as displayed in Figure 4. In **TS4**, the B–H bond is cleaved and, at the same time, the H atom migrates to occupy the coordination position trans to the resulting boryl fragment (the key bonding metrics can be found in Figure 4). During this step, the bound-deuterated silane assists the process through a weak Si···H interaction (1.97 Å). As a result, the Gibbs free energy necessary is 23.4 kcal·mol^–1^. After **TS4**, the corresponding σ-Si–D complex **Int5** is observed, almost identical to **Int2** in the mechanism depicted in Figure 3. Again, silane extrusion would give partially deuterated PhMeSiHD and complex **5**. This metal–ligand cooperativity mechanism exhibits a Δ*G* barrier (24.1 kcal·mol^–1^) comparable to that observed for the calculated pathway involving nucleophilic attack on Si (22.4 kcal·mol^–1^), and both of them are consistent with the experimental value determined via kinetic studies (20.2 ± 0.3 kcal·mol^–1^). These mechanistic proposals provide plausible pathways by which H/D exchange on Si can take place and manifest the key role of the PBP framework, either as a strong trans influence ligand or as a noninnocent fragment of the catalyst. Unfortunately, a mechanistic scenario for the regeneration of **5-D** from **5** and D_2_ (i.e., H/D exchange on Ni) could not be found, in line with previous reports for silane deuteration^19,39^ (examples of mechanistic possibilities with high kinetic barriers can be found in Figures S38–S40).

## Conclusions

In this study, pincer nickel hydride complex **5** was identified as an effective catalyst for H/D exchange in primary to tertiary silanes and boranes. The reaction was carried out under mild catalytic conditions with low catalyst loading (2 mol %), resulting in spectroscopic yields of up to 99%. A detailed mechanistic investigation of the H/D exchange reaction is presented, revealing two plausible pathways with similar energy barriers. In the first pathway, the strong trans-influence of the boryl group enhances the nucleophilic character of the hydride, facilitating a nucleophilic attack on the silicon atom of the silane (Δ*G* ^⧧^ = 22.4 kcal mol^–1^). Alternatively, the boryl-nickel moiety may promote cooperative activation of the Si–H bond, leading to the formation of a Ni(0) σ-Si–D intermediate, followed by activation of the B–H bond with assistance from the coordinated silane. This pathway is associated with a slightly higher Gibbs free energy (Δ*G* ^⧧^ = 24.1 kcal mol^–1^). Kinetic experiments estimate the overall energy cost of the deuteration reaction to be 20.2 ± 0.3 kcal·mol^–1^, suggesting that both pathways are feasible. Additionally, experimental evidence supporting the proposed mechanism was obtained from reactions with boranes. Thus, the formation of the intermediate (^*t*Bu^PBP)­NiH_2_Bcat (**8**) was shown to inhibit the H/D exchange, which is consistent with the proposed mechanism when boranes are used. These results underscore the key role of the boryl group in modulating the reactivity of the PBP-Ni system and provide valuable insights for the future design of related catalysts.

## Supplementary Material



## Data Availability

The data underlying this study are available in the published article and its Supporting Information.

## Notes

Full synthetic methods, characterization data, catalysis procedures, and NMR data (PDF)
