# Supplementary material for: Nickel-Catalyzed Deuteration of Primary, Secondary, and Tertiary Silanes: Scope and Mechanistic Insights
Source: J Org Chem. 2025 Apr 8;90(15):5206–12. doi: 10.1021/acs.joc.5c00107 (PMC12510143; doi:10.1021/acs.joc.5c00107)
Supplement: Supplementary file 1 [file jo5c00107_si_001.pdf]

Supporting Information

Nickel-Catalyzed Deuteration of Primary, Secondary and Tertiary Silanes: Scope and Mechanistic Insights

Carlos J. Laglera-Gándara, Rafael Jiménez-Rioboó, Lucía Álvarez-Rodríguez, Riccardo Peloso, Pablo Ríos, Amor Rodríguez**

Table of Contents

1. Experimental procedures 2
2. Catalytic reactions 2
2.1 General Method for Deuteration of Hydrosilanes and Alkoxysilanes 2
2.2 Deuteration of 4,4,5,5-Tetramethyl-1,3,2-dioxa(borolane) (HBpin) and Benzo-1,3,2-diazoborolane. 3
2.3 Preparative scale synthesis of Et_2SiD_2 4
3. Substrate Scope Spectroscopic Data 4
4. *In Situ* NMR Studies 23
5. Kinetics studies 29
6. Reactivity studies of complex **5** with $ClMe_2SiH$ and HBcat 37
7. Computational details 40
8. Cartesian Coordinates of the optimized structures 44
9. References 99

1. Experimental procedures

General considerations. All manipulations were carried out using standard Schlenk and glovebox techniques, under an atmosphere of argon and of high purity nitrogen, respectively. All solvents were dried and degassed prior to use. n-Pentane was distilled over sodium and stored under Na/K alloy. NMR spectra were recorded on Bruker DRX-500, DRX-400 and DPX-300 spectrometers, and they were referenced to external $SiMe_4$ (δ 0 ppm) using the residual protio solvent peaks as internal standard (^{1}H NMR experiments) or the characteristic resonances of the solvent nuclei (^{13}C NMR experiments). ^{11}B NMR spectra were referenced to an external standard of $BF_3{\cdot}Et_2O$ (δ 0 ppm). ^{31}P NMR chemical shifts were referenced to an external 85% solution of H_3PO_4 (δ 0 ppm) in the appropriate solvent. The (^{tBu}PBP)NiH was prepared as previously described.[1]

2. Catalytic reactions

2.1 General Method for Deuteration of Hydrosilanes and Alkoxysilanes.

$$\underset{(0.20\ \text{mmol})}{R_3SiH} \xrightarrow[\substack{C_6D_6\ (0.4\ \text{mL}) \\ D_2\ (2\ \text{bar}),\ \text{rt},\ 4\text{-}6\text{h}}]{\textbf{[Ni]}\ (2\ \text{mol}\ \%)} R_3SiD$$

All reactions were performed in duplicate in deuterated solvent. To a fischer-porter vessel 20 mL containing catalyst **5** (2 mol %), C_6D_6 (400 μL) and silane or alkoxysilanes (0.20 mmol) were added and degassed via three freeze-pump-thaw cycles. Then, the fischer-porter tube was backfilled with D_2 gas (2 bar). The mixture was stirred at room temperature for 4 hours (tertiary or secondary silanes and alkoxysilanes) or 6h (primary silanes), turning from dark yellow to yellow after 5 minutes. Spectroscopic yields were determined by ^{1}H NMR spectroscopy (see Table 1).

Table S1a. Optimization of Silane and Alkoxysilanes Deuteration[a]

Entry	Starting material	Products	Yield (%)[b]
1	Et_2SiH_2 (**1a**)	Et_2SiD_2 (**2a**)	99
2	Ph_2SiH_2 (**1b**)	Ph_2SD_2 (**2b**)	95
3	$PhMeSiH_2$ (**1c**)	$PhMeSiD_2$ (**2c**)	94
4	$^{t}Bu_2SiH_2$ (**1d**)	$^{t}Bu_2SiD_2$ (**2d**)	54
5	Ph_3SiH (**1e**)	Ph_3SiD (**2e**)	94
6	Et_3SiH (**1f**)	Et_3SiD (**2f**)	96
7	Ph_2MeSiH (**1g**)	Ph_2MeSiD (**2g**)	96
8	$PhMe_2SiH$ (**1h**)	$PhMe_2SiD$ (**2h**)	97
9	$(EtO)_3SiH$ (**1i**)	$(EtO)_3SiD$ (**2i**)<br>$(EtO)_6Si_2$ (**2i'**)	91<br>5
10	$(MeO)_3SiH$ (**1j**)	$(MeO)_3SiD$ (**2j**)<br>$(MeO)_3Si\text{-}Si(MeO)_3$ (**2j'**)	91<br>6
11	$(MeO)_2MeSiH$ (**1k**)	$(MeO)_2MeSiD$ (**2k**)	96
12	$^{n}C_6H_{13}SiH_3$ (**1l**)	$^{n}C_6H_{13}SiD_3$ (**2l**)	98

		$^{n}C_6H_{13}SiD_2H$ (**2l’**)	<1
		$^{n}C_6H_{13}SiDH_2$ (**2l’’**)	<1
		$PhSiD_3$ (**2m**)	57
13	$PhSiH_3$ (**1m**)	$PhSiD_2H$ (**2m’**)	5
		$PhSiDH_2$ (**2m’’**)	<1

a All reactions were performed under D_2 (after freeze-pump-thaw cycle) for 4h or 6h at room temperature.

b NMR spectroscopic yield by ^1H NMR analysis.

2.2 Deuteration of 4,4,5,5-Tetramethyl-1,3,2-dioxa(borolane) (HBpin) and Benzo-1,3,2-diazoborolane.

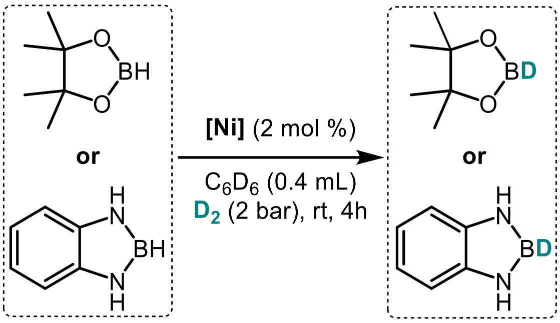

The reaction was performed in duplicate in deuterated solvent. Catalyst **5** (2 mol %), C_6D_6 (400 μL) and HBpin or the amine-borane (0.20 mmol) were added to a 20 mL fischer-porter vessel, then the fischer-porter tube was degassed via three freeze-pump-thaw cycles and afterwards was backfilled with D_2 gas (2 bar). The mixture was stirred at room temperature for 4 hours, turning from dark yellow to yellow after 5 minutes. Spectroscopic yield was determined by ^2H NMR spectroscopy using toluene-d_8 (21.3 μL, 0.20 mmol) to DBpin (**2p**) and D-B($C_6H_6N_2$) (**2q**) (> 99%, both cases).

Table S1b. Results of the Deuteration Experiments of Each Silane, Alkoxysilane, and Borane

Entry	Starting material	Yield (%)	Yield Duplicate (%)	Average (%)
1	Et_2SiH_2 (**1a**)	>99	>99	>99(0)
2	Ph_2SiH_2 (**1b**)	96	94	95(1)
3	$PhMeSiH_2$ (**1c**)	94	95	94(1)
4	$^{t}Bu_2SiH_2$ (**1d**)	53	55	54(1)
5	Ph_3SiH (**1e**)	94	95	94(1)
6	Et_3SiH (**1f**)	97	96	96(1)
7	Ph_2MeSiH (**1g**)	96	97	96(1)
8	$PhMe_2SiH$ (**1h**)	97	97	97(0)
9	$(EtO)_3SiH$ (**1i**)	91	91	91(0)
10	$(MeO)_3SiH$ (**1j**)	91	92	91(1)
11	$(MeO)_2MeSiH$ (**1k**)	96	97	96(1)
12	$^{n}C_6H_{13}SiH_3$ (**1l**)	96	98	97(1)
13	$PhSiH_3$ (**1m**)	58	56	57(1)
14	HBpin (**1p**)	>99	>99	>99(0)
15	H-B($C_6H_6N_2$) (**1q**)	>99	>99	>99(0)

2.3 Preparative scale synthesis of Et_2SiD_2.

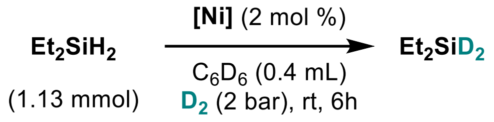

In a glove box, diethylsilane (0.100 g, 1.13 mmol) and **5** (0.011, 0.022 mmol) were mixed in 2 mL of C_6D_6 in a fischer-porter vessel (60 mL) and degassed via three freeze-pump-thaw cycles. Then, the fischer-porter tube was backfilled with D_2 gas (2 bar). The mixture was stirred at room temperature for 6 hours, turning from dark yellow to yellow after 30 min. The mixture was transferred to a H-distillation tube and separated from **5** by vacuum distillation at -20 ^{o}C (isolated yield 60%). Hexamethyldisiloxane (HMDSO) (20%) was added and spectroscopic yield was determined by ^{1}H NMR spectroscopy (98%).

3. Substrate Scope Spectroscopic Data

Diethyl(silane-d$_2$), 2a

Spectroscopic yield: >99%.

<1% Et_2SiH_2 was detected.

^{1}H NMR (400 MHz, C_6D_6): δ (ppm) = 0.95 (t, 9H, $J_{H,H}$ = 7.9 Hz, C***H*$_3$** of Si***Et*$_2$**), 0.53 (q, 6H, $J_{H,H}$ = 7.9 Hz, C***H*$_2$** of Si***Et*$_2$**).

^{13}C{^{1}H} NMR (75 MHz, C_6D_6): δ (ppm) = 9.21 (**C**H$_3$ of Si***Et*$_2$**), 1.13 (**C**H$_2$ of Si***Et*$_2$**).

Diphenyl(silane-d$_2$), 2b

Spectroscopic yield: 95%.

Deuterium incorporation: 97%

4% Ph_2SiHD and 1% Ph_2SiH_2 were detected.

^{1}H NMR (400 MHz, C_6D_6): δ (ppm) = 7.64–7.45 (m, 4H, ^{m}Ar***H***), 7.26–6.95 (m, 6H, ^{o,p}Ar***H***), 5.06 (s (broad), 0.04H, residual Ph$_2$Si***H***D), 5.07 (s (broad), 0.01H, residual Ph$_2$Si***H*$_2$**).

^{13}C{^{1}H} NMR (75 MHz, C_6D_6): δ (ppm) = 136.1 (**C**H of Si***Ph*$_2$**), 131.7 (***C*$_{ipso}$** of Si***Ph*$_2$**), 130.1 (**C**H of Si***Ph*$_2$**), 128.5 (**C**H of Si***Ph*$_2$**).

Methylphenyl(silane-d$_2$), 2c

Spectroscopic yield: 91%.

Deuterium incorporation: 95%

9 % PhMeSiHD was detected.

^{1}H NMR (400 MHz, C_6D_6): δ (ppm) = 7.51–7.37 (m, 2H, ^{m}Ar***H***), 7.17–7.01 (m, 3H, ^{o,p}Ar***H***), 4.46 (qt, 0.06 H, $^3J_{H,H}$ = 4.2 Hz, $^2J_{H,D}$ = 0.9 Hz, 0.06H residual Si***HD***), 0.17 (s, 3H, C***H*$_3$** of SiPh***Me*$_2$**).

^{13}C{^{1}H} NMR (75 MHz, C_6D_6): δ (ppm) = 135.2 (**C**H of SiMe***Ph***), 133.4 (***C*$_{ipso}$** of SiMe***Ph***), 129.8 (**C**H of SiMe***Ph***), 128.3 (Ph), -7.74 (***Me*** of Si***Me***Ph).

Ditertbutyl(silane-d$_2$), 2d

Spectroscopic yield: 54%.

46% ^{t}Bu$_2$SiH$_2$ was detected.

^{1}H NMR (400 MHz, C_6D_6): δ (ppm) = 3.66 (br s, 0.85H, residual Si***H***), 1.04 (s, 18H, C***H$_3$*** of Si***^{t}Bu$_2$***).

^{13}C{^{1}H} NMR (75 MHz, C_6D_6): δ (ppm) = 28.9 (***C***H$_3$ of Si***^{t}Bu$_2$***), 17.7 (***C$_{ipso}$*** of Si***^{t}Bu$_2$***).

Triphenyl(silane-d), 2e

Spectroscopic yield: 94%.

6% Ph$_3$SiH was detected.

^{1}H NMR (400 MHz, C_6D_6): δ (ppm) = 7.58 (dd, 6H, $J_{H,H}$ = 7.6, 2.0 Hz, ^{m}Ar***H***), 7.30–7.03 (m, 9H, ^{o,p}Ar***H***), 5.71 (s, 0.05H, residual Si***H***).

^{13}C{^{1}H} NMR (75 MHz, C_6D_6): δ (ppm) = 136.2 (***C***H of Si***Ph$_3$***), 135.8 (***C$_{ipso}$*** of Si***Ph$_3$***), 130.1 (***C***H of Si***Ph$_3$***), 128.4 (***C***H of Si***Ph$_3$***).

Triethyl(silane-d), 2f

Spectroscopic yield: 96%.

4% Et$_3$SiH was detected.

^{1}H NMR (400 MHz, C_6D_6): δ (ppm) = 3.80 (t, 0.04H, $J_{H,H}$ = 1.8 Hz, residual Si***H***), 0.98 (t, 9H, $J_{H,H}$ = 7.9 Hz, C***H$_3$*** of Si***Et$_3$***), 0.54 (q, 6H, $J_{H,H}$ = 7.9 Hz, C***H$_2$*** of Si***Et$_3$***).

^{13}C{^{1}H} NMR (75 MHz, C_6D_6): δ (ppm) = 8.41 (***C***H$_3$ of Si***Et$_2$***), 2.74 (***C***H$_2$ of Si***Et$_2$***).

Methyldiphenyl(silane-d), 2g

Spectroscopic yield: 96%.

4% MePh$_2$SiH was detected.

^{1}H NMR (400 MHz, C_6D_6): δ (ppm) = 7.65–7.44 (m, 4H, ^{m}Ar***H***), 7.26–6.99 (m, 6H, ^{o,p}Ar***H***), 5.14 (s, 0.04H, residual Si***H***), 0.46 (s, 3H, C***H$_3$*** of SiPh$_2$***Me***).

^{13}C{^{1}H} NMR (75 MHz, C_6D_6): δ (ppm) = 135.6 (***C$_{ipso}$*** of Si***Ph$_2$***Me), 135.2 (***C***H of Si***Ph$_2$***Me), 129.8 (***C***H of Si***Ph$_2$***Me), 128.3 (***C***H of Si***Ph$_2$***Me), -4.97 (***Me*** of SiPh$_2$***Me***).

Dimethylphenyl(silane-d), 2h

Spectroscopic yield: 97%.

3% Me$_2$PhSiH was detected.

^{1}H NMR (400 MHz, C_6D_6): δ (ppm) = 7.51–7.42 (m, 2H, ^{m}Ar***H***), 7.23–7.14 (m, 3H, ^{o,p}Ar***H***), 4.62 (p, 0.03H, $J_{H,H}$ = 3.8 Hz, residual Si***H***), 0.20 (s, 3H, C***H$_3$*** of SiPh***Me$_2$***).

^{13}C{^{1}H} NMR (75 MHz, C_6D_6): δ (ppm) = 137.5 (***C$_{ipso}$*** of Si***Ph***Me$_2$), 134.4 (***C***H of Si***Ph***Me$_2$), 129.5 (***C***H of Si***Ph***Me$_2$), 128.2 (***C***H of Si***Ph***Me$_2$), -3.81 (***Me*** of SiPh***Me$_2$***).

Triethoxy(silane-d), 2i

Spectroscopic yield: 91%.

5% of coupling product $(EtO)_3Si-Si(EtO)_3$ detected.

4% $(EtO)_3SiH$ was detected.

^{1}H NMR (400 MHz, C_6D_6): δ (ppm) = 4.58 (br s, 0.04, residual Si***H***), 3.77 (q, 6H, $J_{H,H}$ = 7.0 Hz, C***H₂*** of ***Et₃***O), 1.13 (t, 9H, $J_{H,H}$ = 7.0 Hz, C***H₃*** of ***Et₃***O).

^{13}C{^{1}H} NMR (75 MHz, C_6D_6): δ (ppm) = 58.4 (***C***H_2O of SiO***Et₃***), 18.5 (***C***H_3 of SiO***Et₃***).

Trimethoxy(silane-d), 2j

Spectroscopic yield: 91%.

6% of coupling product $(MeO)_3Si-Si(MeO)_3$ detected.

3% $(MeO)_3SiH$ was detected.

^{1}H NMR (400 MHz, C_6D_6): δ (ppm) = 4.43 (br s, 0.03, residual Si***H***), 3.36 (s, 9H, C***H₃*** of C***H***$_3$O).

^{13}C{^{1}H} NMR (75 MHz, C_6D_6): δ (ppm) = 49.7.4 (***C***H_3O of SiO***Me₃***)

Dimethoxymethyl(silane-d), 2k

Spectroscopic yield: 96%.

4% $(MeO)_2MeSiH$ was detected.

^{1}H NMR (400 MHz, C_6D_6): δ (ppm) = 4.73 (br s, 0.04H, residual Si***H***), 3.34 (s, 6H, C***H₃*** of (C***H***$_3$O)$_2$CH$_3$), 0.08 (s, 3H, C***H₃*** of (CH$_3$O)$_2$C***H***$_3$)

^{13}C{^{1}H} NMR (75 MHz, C_6D_6): δ (ppm) = 50.7 ((***C***H$_3$)$_2$OCH$_3$ of SiMe(O***Me***)$_2$), -3.76 (***C***H$_3$ of Si***Me***(OMe)$_2$).

Hexyl(silane-d₃), 2l

For this compound the general procedure was modified. The starting silane $nC_6H_{13}SiH_3$ in C_6D_6 was exposed to deuterium gas D_2 for 6 h instead of 4 h.

Spectroscopic yield: 97%

<1% ($nC_6H_{13}SiH_3$), <1 % ($nC_6H_{13}SiDH_2$) and 1 % ($nC_6H_{13}SiD_2H$) detected.

^{1}H NMR (400 MHz, C_6D_6): δ (ppm) = 3.65–3.59 (m, 0.11H, residual Si***H₃***, Si***H₂***D and Si***H***D$_2$), 1.31–1.14 (m, 8H, C***H₂*** of SiCH$_2$(C***H***$_2$)$_4$CH$_3$), 0.87 (t, 3H, $J_{H,H}$ = 7.0 Hz, SiCH$_2$(CH$_2$)$_4$C***H***$_3$), 0.52 (t, 3H, $J_{H,H}$ = 7.6 Hz, SiC***H***$_2$(CH$_2$)$_4$CH$_3$).

^{13}C{^{1}H} NMR (75 MHz, C_6D_6): δ (ppm) = 32.5 (***C***H$_2$ of $^nC_6H_{13}$Si), 31.8 (***C***H$_2$ of $^nC_6H_{13}$Si), 26.6 (***C***H$_2$ of $^nC_6H_{13}$Si), 22.9 (***C***H$_2$ of $^nC_6H_{13}$Si), 14.3 (***C***H$_3$ of $^nC_6H_{13}$Si), 5.93 (***C***H$_2$ of $^nC_6H_{13}$Si).

Phenyl(silane-d₃), 2m

For this compound the general procedure was modified. The starting silane $PhSiH_3$ in C_6D_6 was exposed to deuterium gas D_2 for 6 h instead of 4 h.

Spectroscopic yield: 57%

<1% ($PhSiH_3$), <1% ($PhSiDH_2$) and 1.85 % ($PhSiD_2H$) detected.

^{1}H NMR (400 MHz, C_6D_6): δ (ppm) = 7.46–7.28 (m 2H, ^{m}Ar***H***), 7.19–6.89 (m, 3H, ^{o,p}Ar***H***), 4.22 (s, 0.01H, residual Si$\underline{\boldsymbol{H_3}}$), 4.21 (s, 0.07H, residual Si$\underline{\boldsymbol{H_2}}$D), 4.21 (s, 0.2H, residual Si***H***D_2).

^{13}C{^{1}H} NMR (75 MHz, C_6D_6): δ (ppm) = 136.1 (***C***H of Si***Ph***), 130.1 ($\underline{\boldsymbol{C_{ipso}}}$ of Si***Ph***), 128.5 (***C***H of Si***Ph***)

DBpin, 2p

Spectroscopic yield: >99%.

<1% HBpin was detected.

^{1}H NMR (400 MHz, C_6D_6): δ (ppm) = 1.00 (s, 12H, C$\underline{\boldsymbol{H}}_3$).

^{13}C{^{1}H} NMR (75 MHz, C_6D_6): δ (ppm) = 83.1 ($\underline{\boldsymbol{C_{ipso}}}$ of B***pin***), 24.9 (***C***H_3 of B***pin***).

Benzo-diazoborolane(borane-d), 2q

Spectroscopic yield: >99%.

<1% borane-H was detected.

^{1}H NMR (400 MHz, C_6D_6): δ (ppm) = 7.01 (m, 2H, Ar***H***), 6.81 (m, 2H, Ar***H***), 5.85 (br s, 2H, N***H***).

^{13}C{^{1}H} NMR (75 MHz, C_6D_6): δ (ppm) = 136.0 ($\underline{\boldsymbol{C_{ipso}}}$ of B($C_6H_6N_2$)), 119.6 (***C***H of B($C_6H_6N_2$)), 111.6 (***C***H of B($C_6H_6N_2$)).

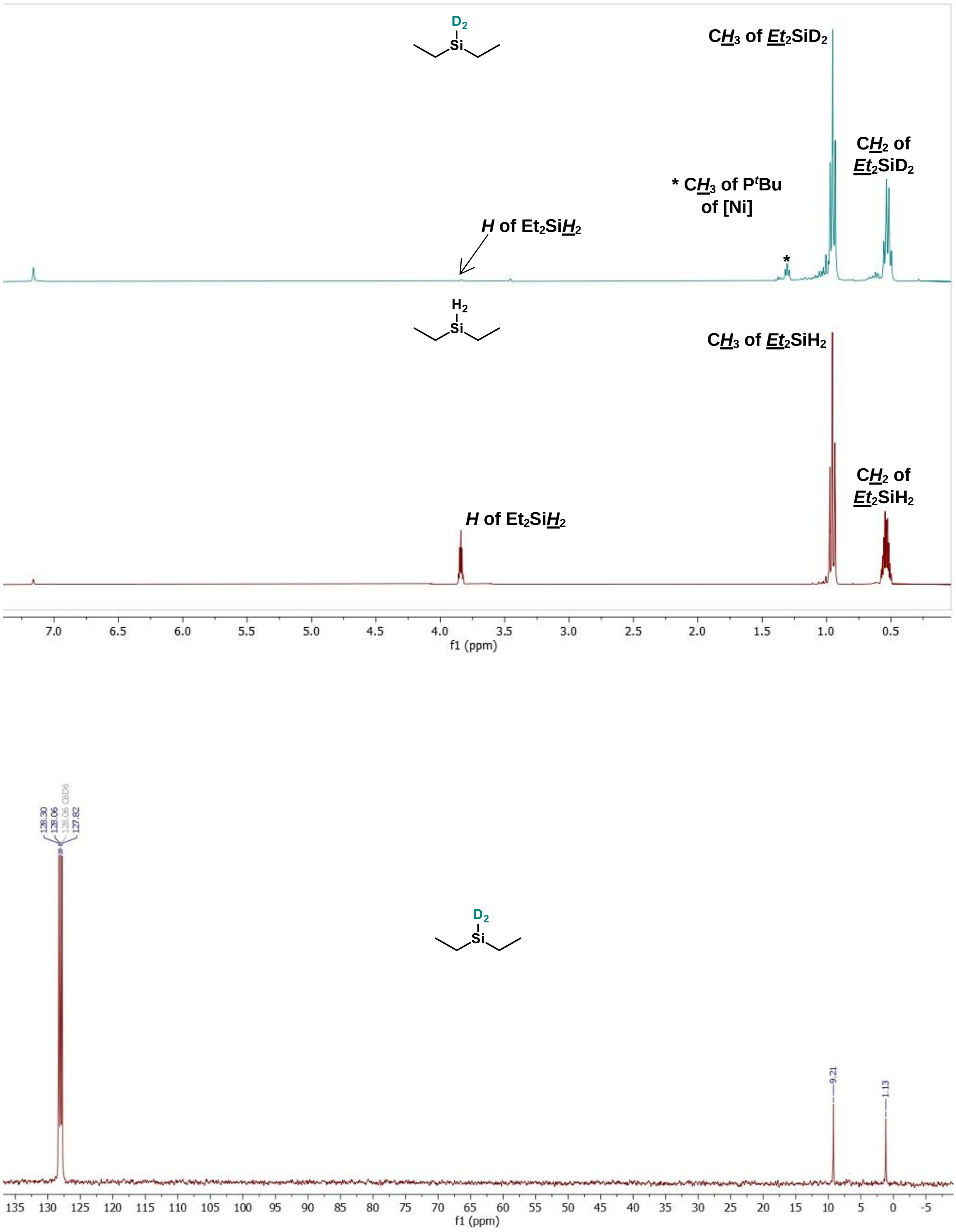

Figure S1. ^{1}H NMR spectrum (400 MHz) (top) and $^{13}C\{^{1}H\}$ NMR spectrum (75 MHz) (below) of Et_2SiD_2 (**2a**) and Et_2SiH_2 (**1a**), (C_6D_6, 298K)

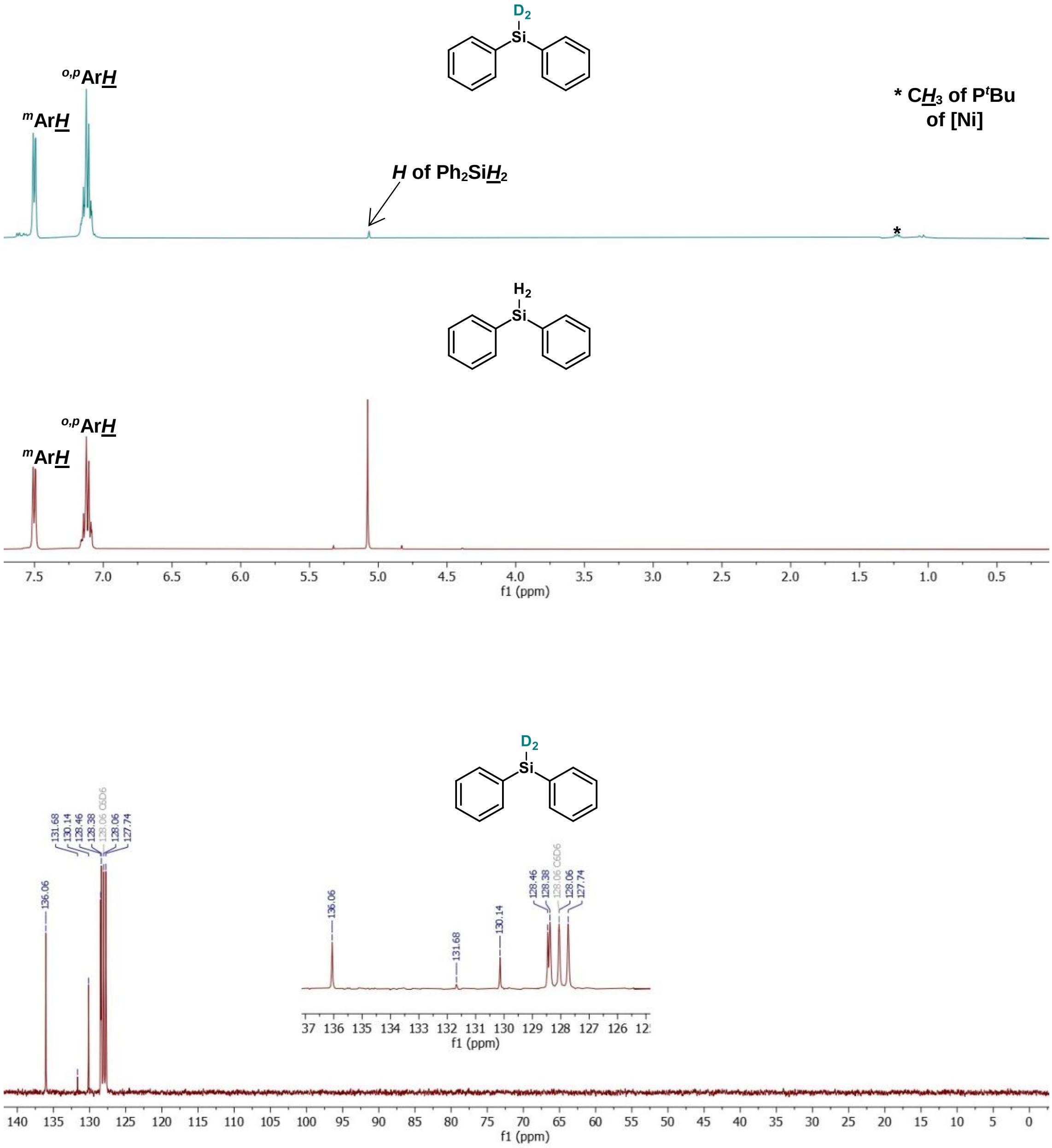

Figure S2. ^{1}H NMR spectrum (400 MHz) (top) and $^{13}C\{^{1}H\}$ NMR spectrum (75 MHz) (below) of Ph_2SiD_2 (**2b**) and Ph_2SiH_2 (**1b**), (C_6D_6, 298K)

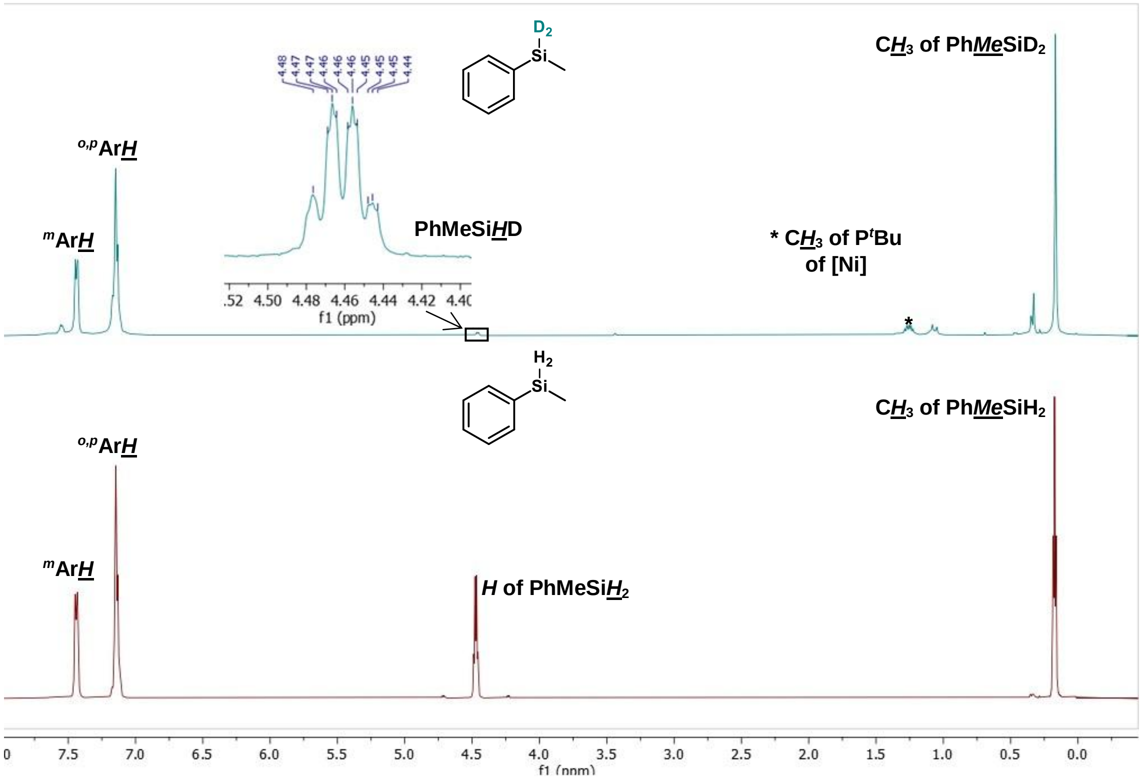

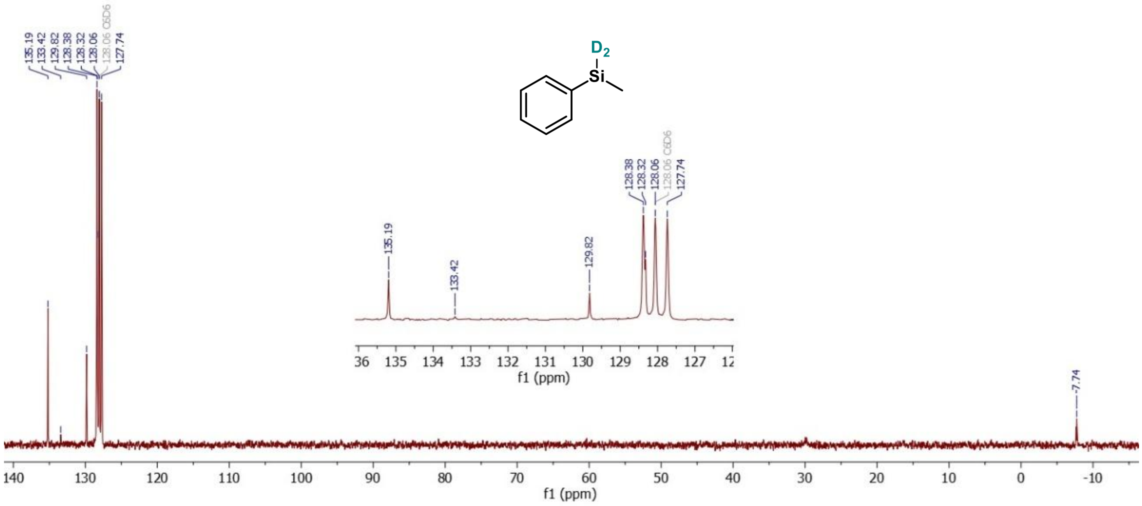

Figure S3. ^{1}H NMR spectrum of $PhMeSiD_2$ (**2c**) and $PhMeSiH_2$ (**1c**) (C_6D_6, 298K) (400 MHz) and $^{13}C\{^{1}H\}$ NMR spectrum (75 MHz) (bottom).

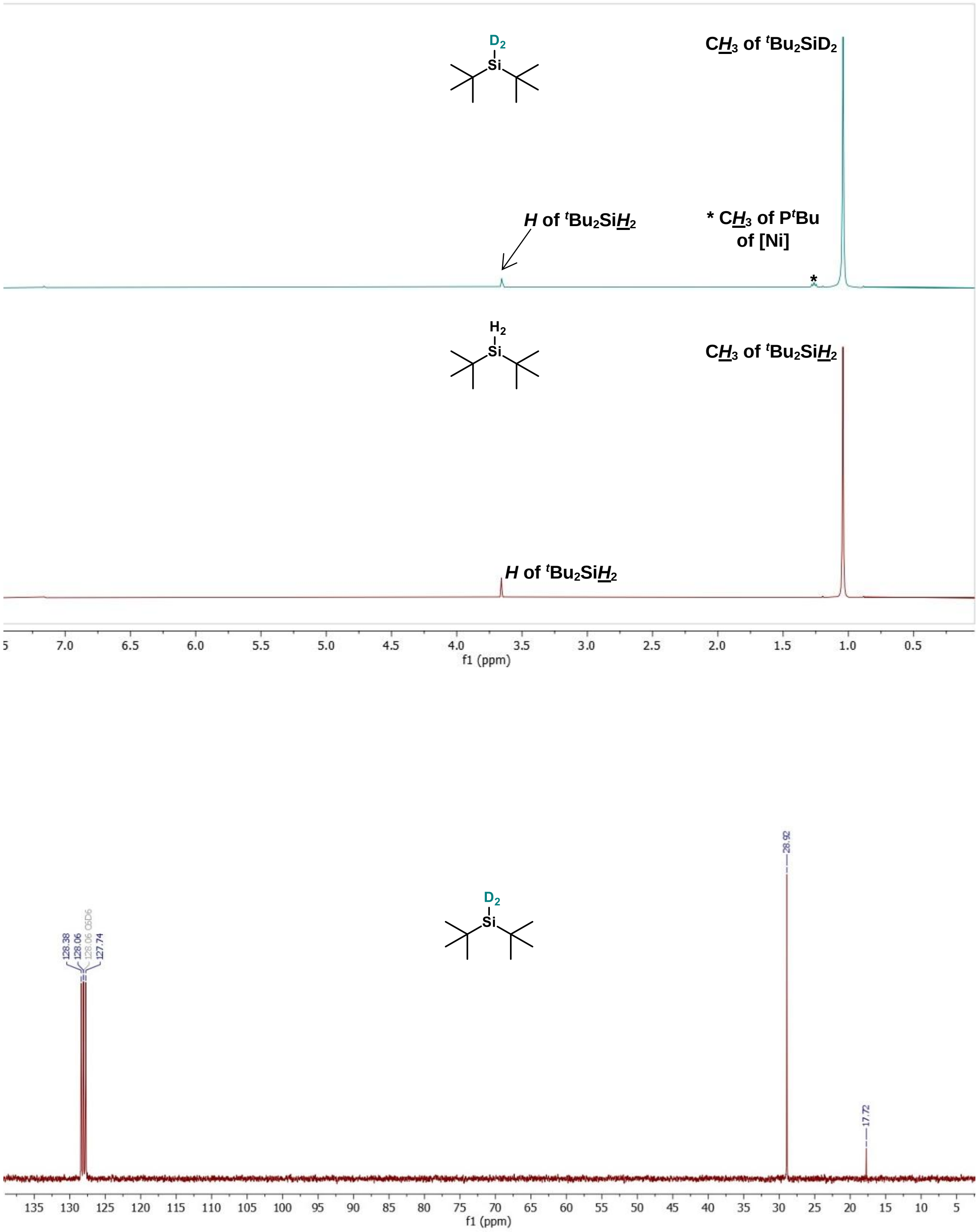

Figure S4. ^{1}H NMR spectrum (400 MHz) (top) and $^{13}C\{^1H\}$ NMR spectrum (75 MHz) (below) of $^t Bu_2SiD_2$ (**2d**) and $^t Bu_2SiH_2$ (**1d**), (C_6D_6, 298K)

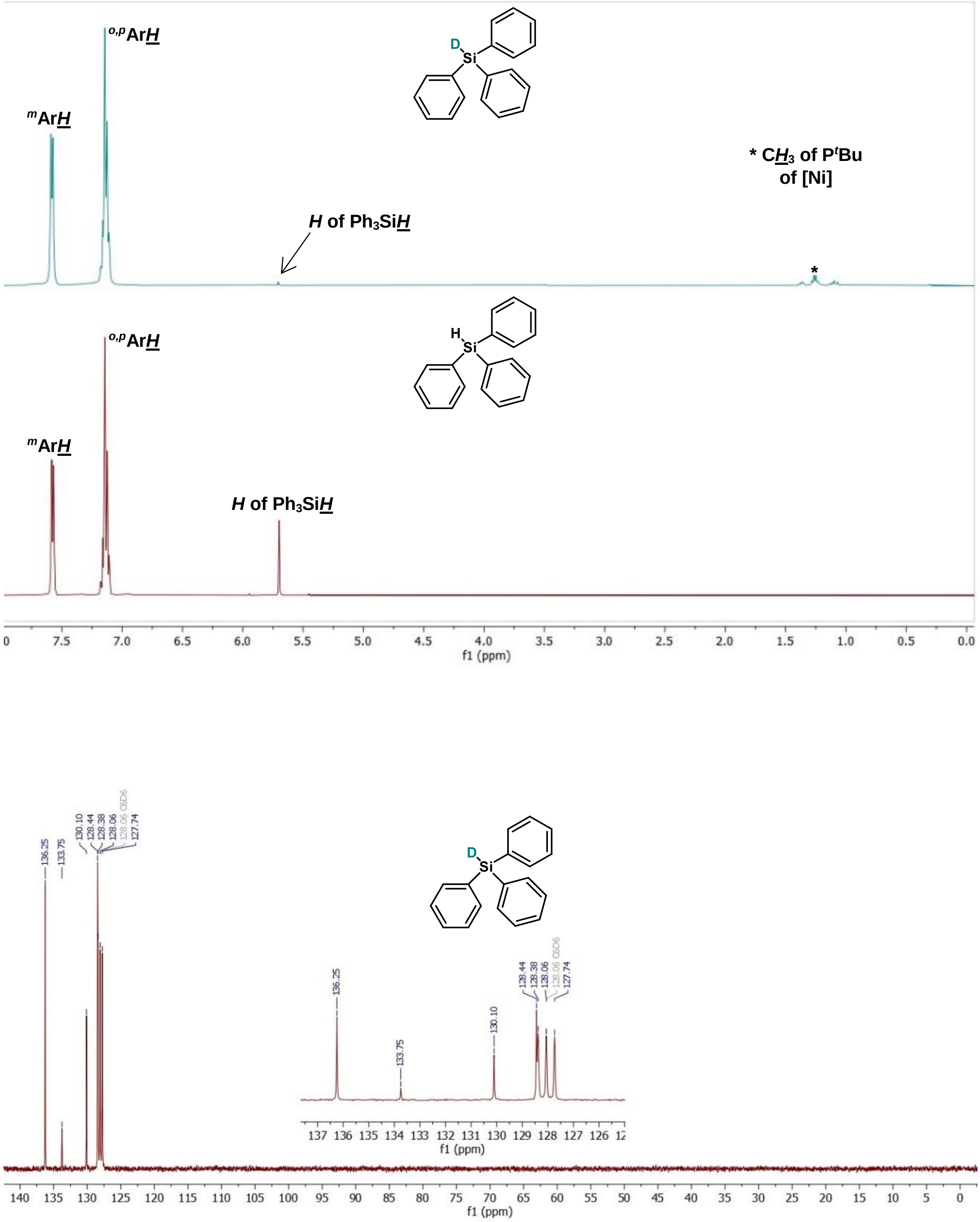

Figure S5. ¹H NMR spectrum (400 MHz) (top) and $^{13}C\{^{1}H\}$ NMR spectrum (75 MHz) (below) of Ph_3SiD (**2e**) and Ph_3SiH (**1e**), (C_6D_6, 298K)

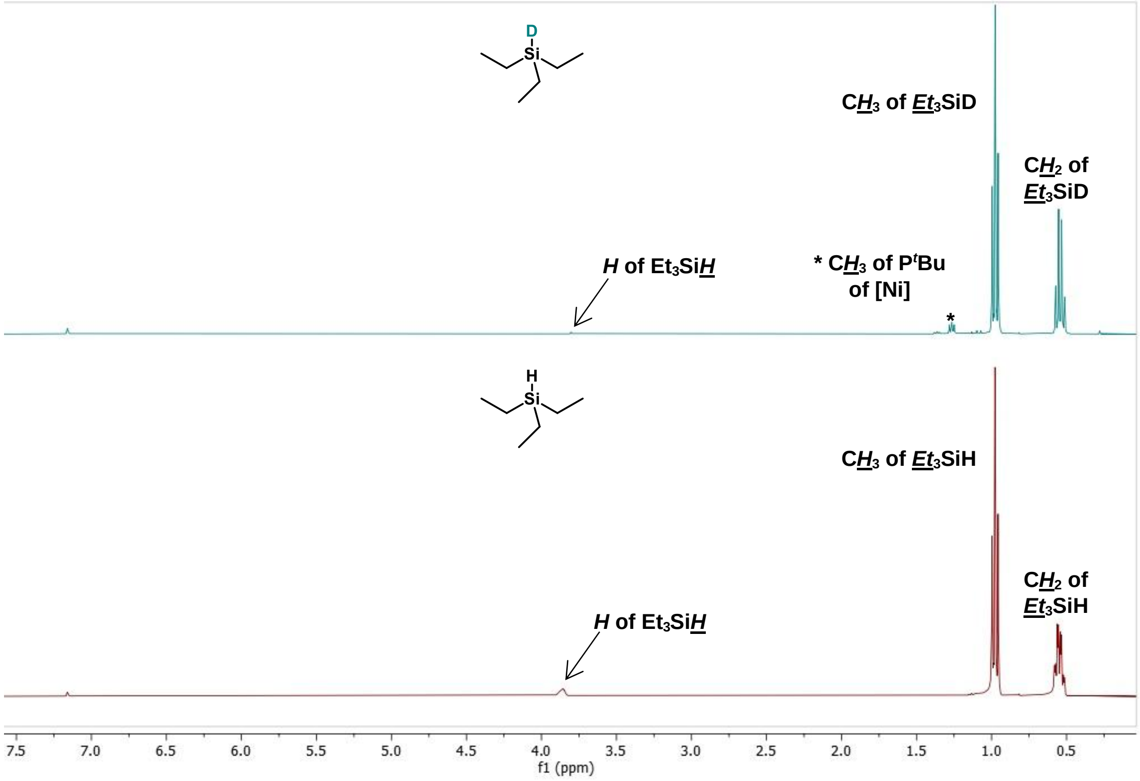

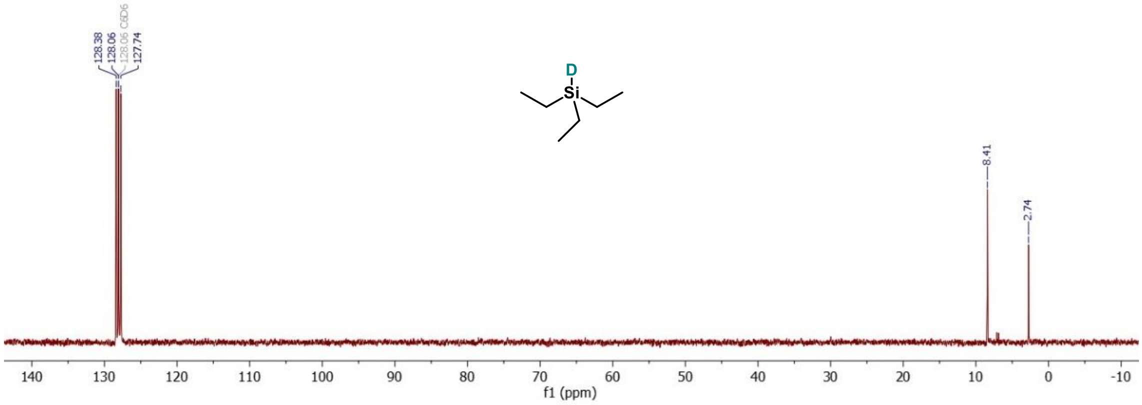

Figure S6. ^{1}H NMR spectrum (400 MHz) (top) and ^{13}C{^{1}H} NMR spectrum (75 MHz) (below) of Et_3SiD (**2f**) and Et_3SiH (**1f**), (C_6D_6, 298K)

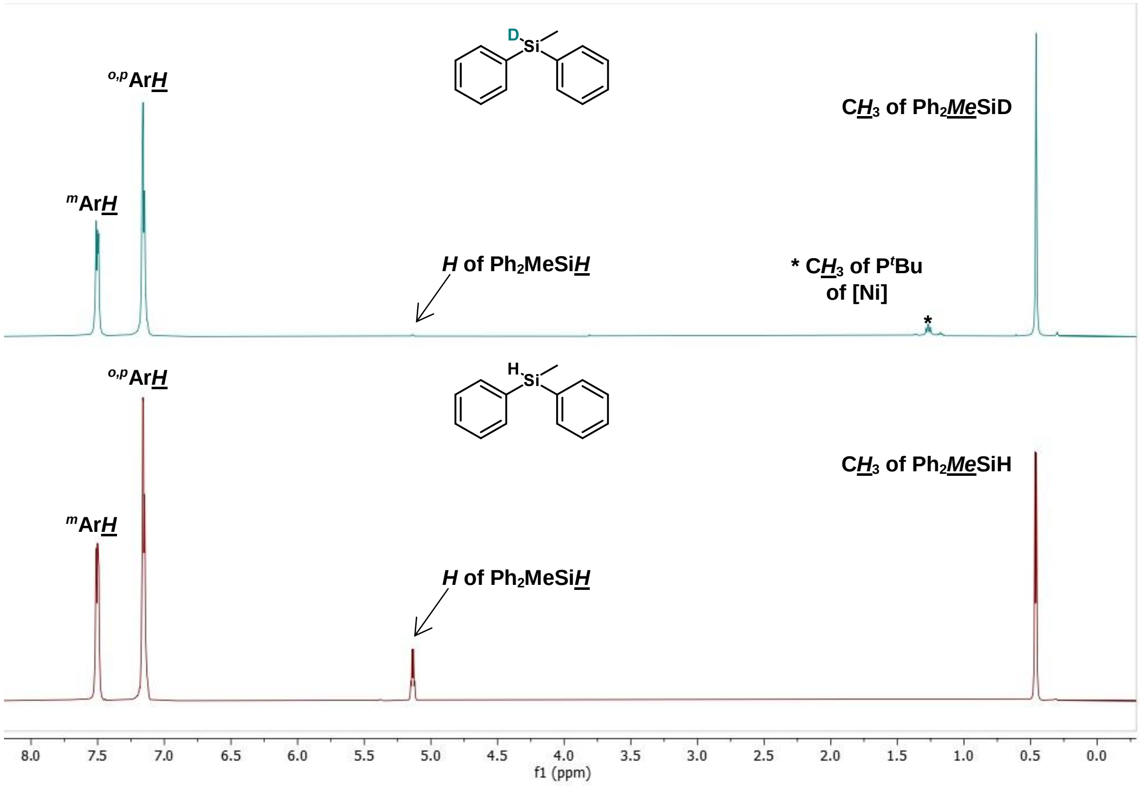

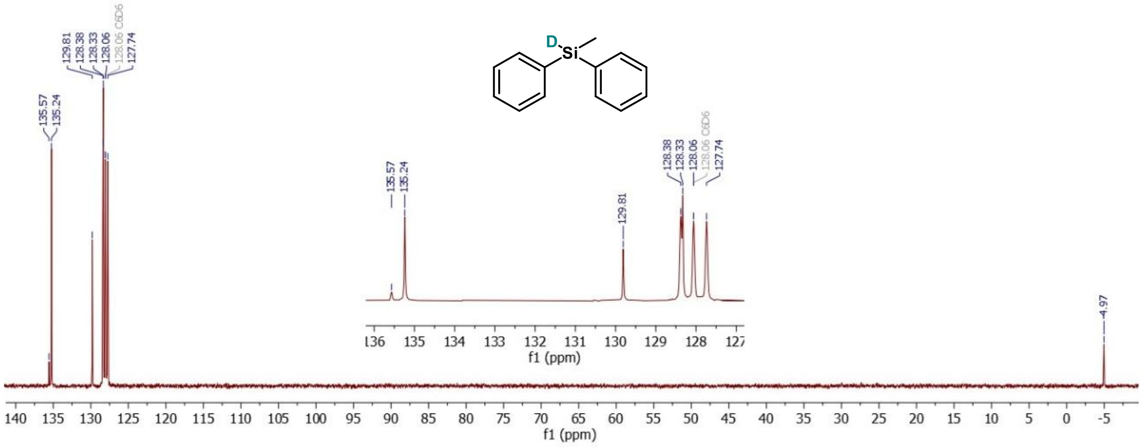

Figure S7. ^{1}H NMR spectrum (400 MHz) (top) and $^{13}C\{^{1}H\}$ NMR spectrum (75 MHz) (below) of Ph_2MeSiD (**2g**) and Ph_2MeSiH (**1g**), (C_6D_6, 298K)

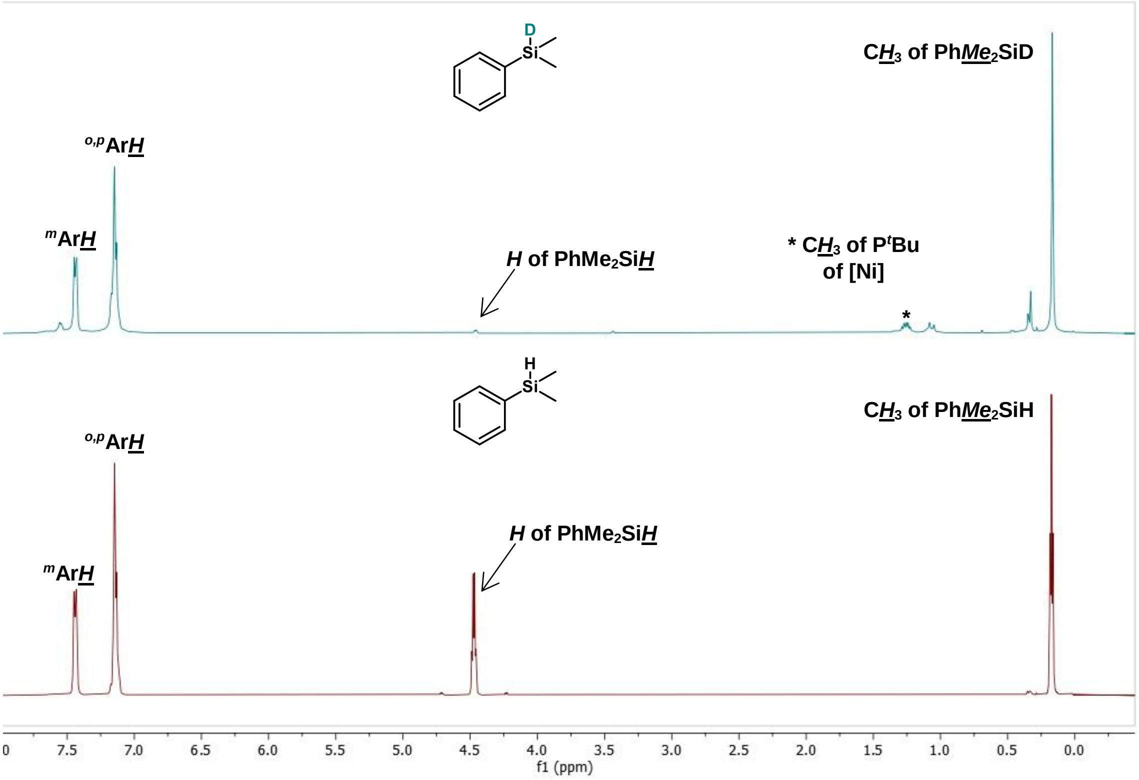

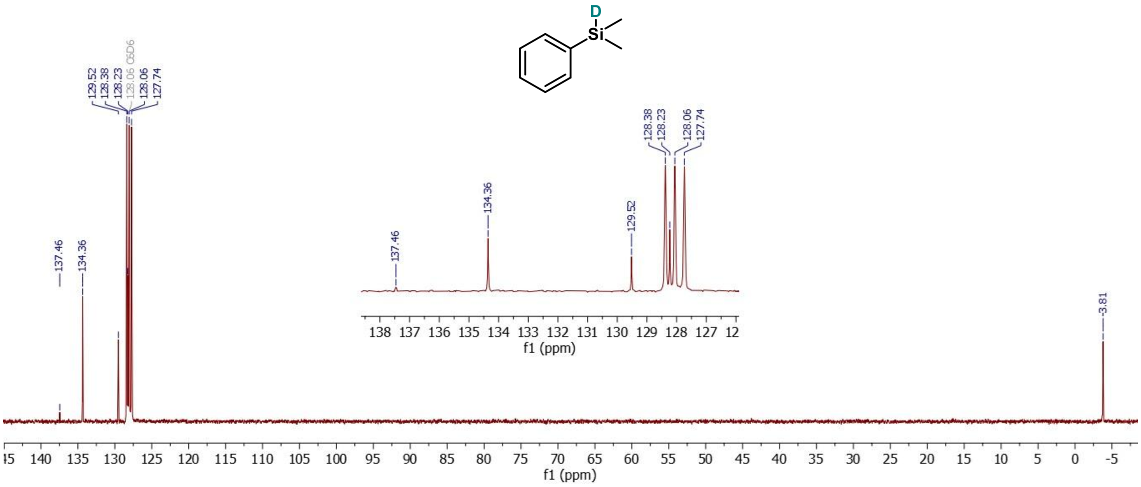

Figure S8. ^{1}H NMR spectrum (400 MHz) (top) and ^{13}C{^{1}H} NMR spectrum (75 MHz) (below) of $PhMe_2SiD$ (**2h**) and $PhMe_2SiH$ (**1h**), (C_6D_6, 298K)

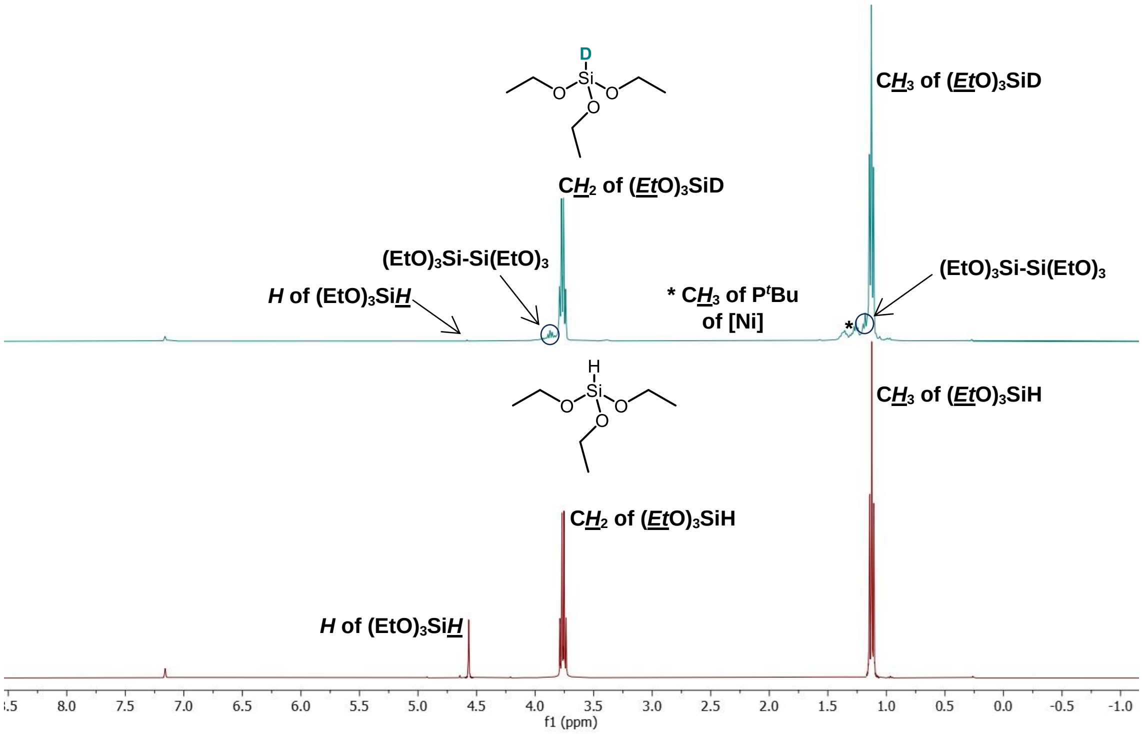

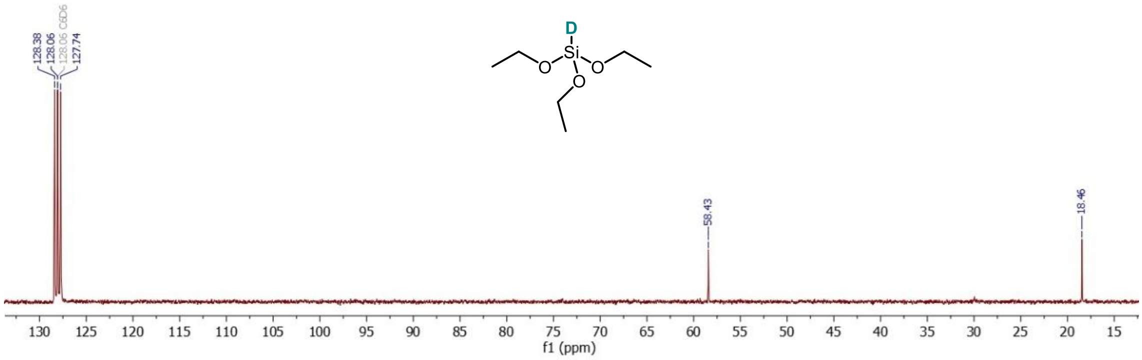

Figure S9. ^{1}H NMR spectrum (400 MHz) (top) and $^{13}C\{^{1}H\}$ NMR spectrum (75 MHz) (below) of $(EtO)_3SiD$ (**2i**) and $(EtO)_3SiH$ (**1i**), (C_6D_6, 298K)

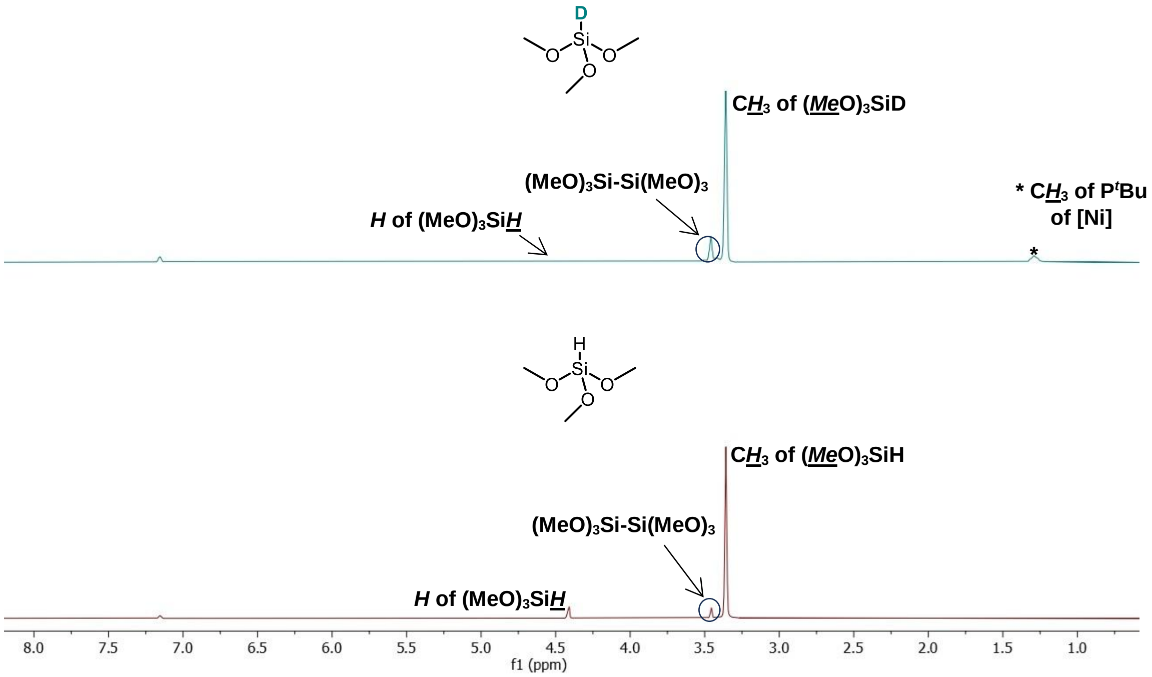

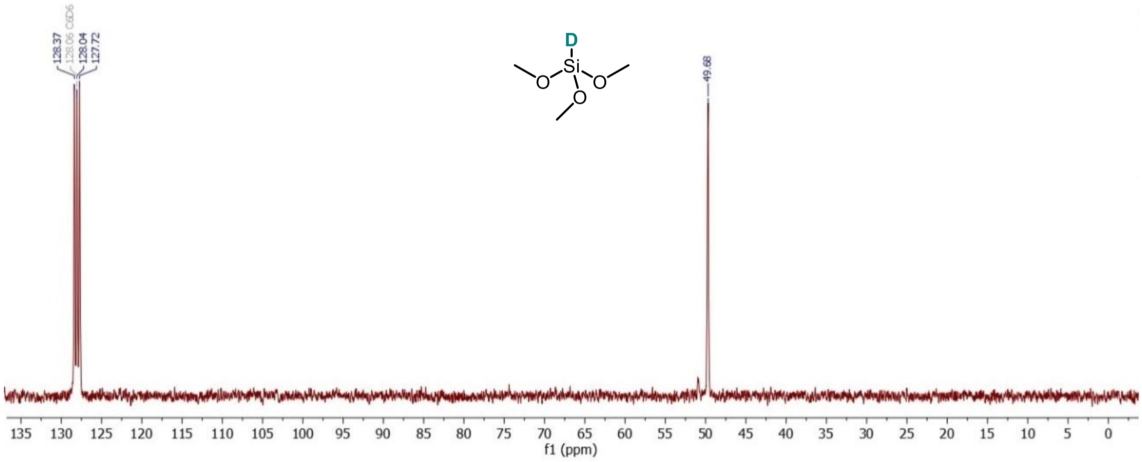

Figure S10. ^{1}H NMR spectrum (400 MHz) (top) and $^{13}C\{^{1}H\}$ NMR spectrum (75 MHz) (below) of $(MeO)_3SiD$ (**2j**) and $(MeO)_3SiH$ (**1j**), (C_6D_6, 298K)

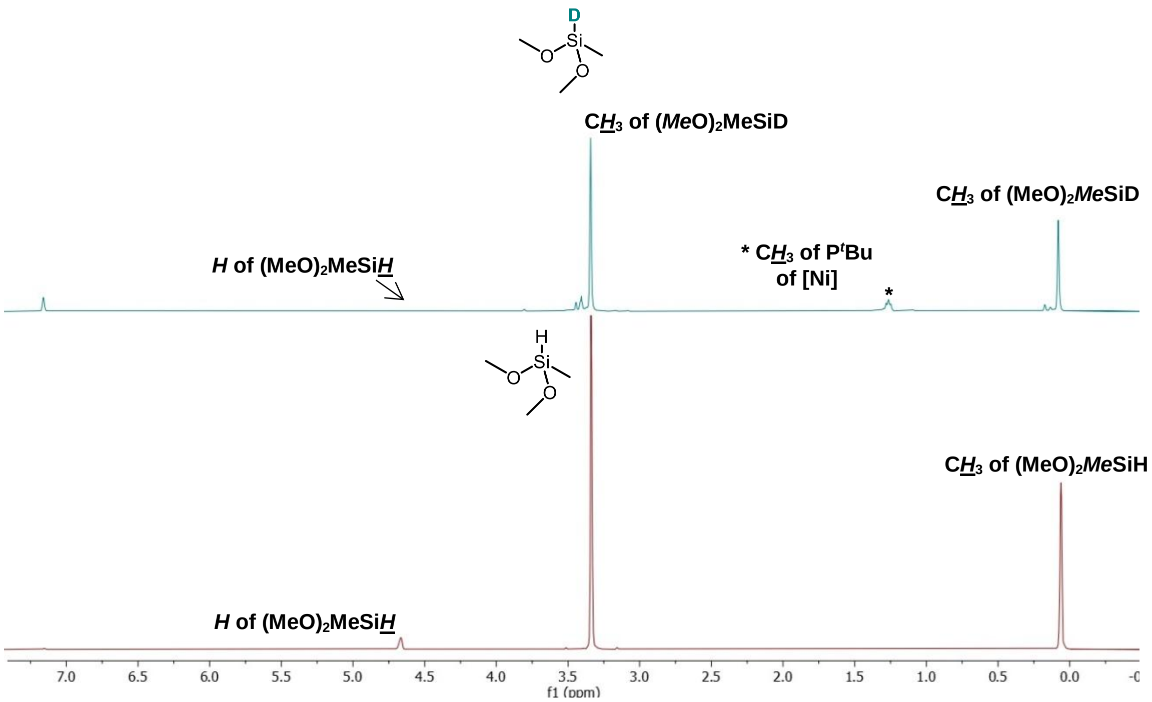

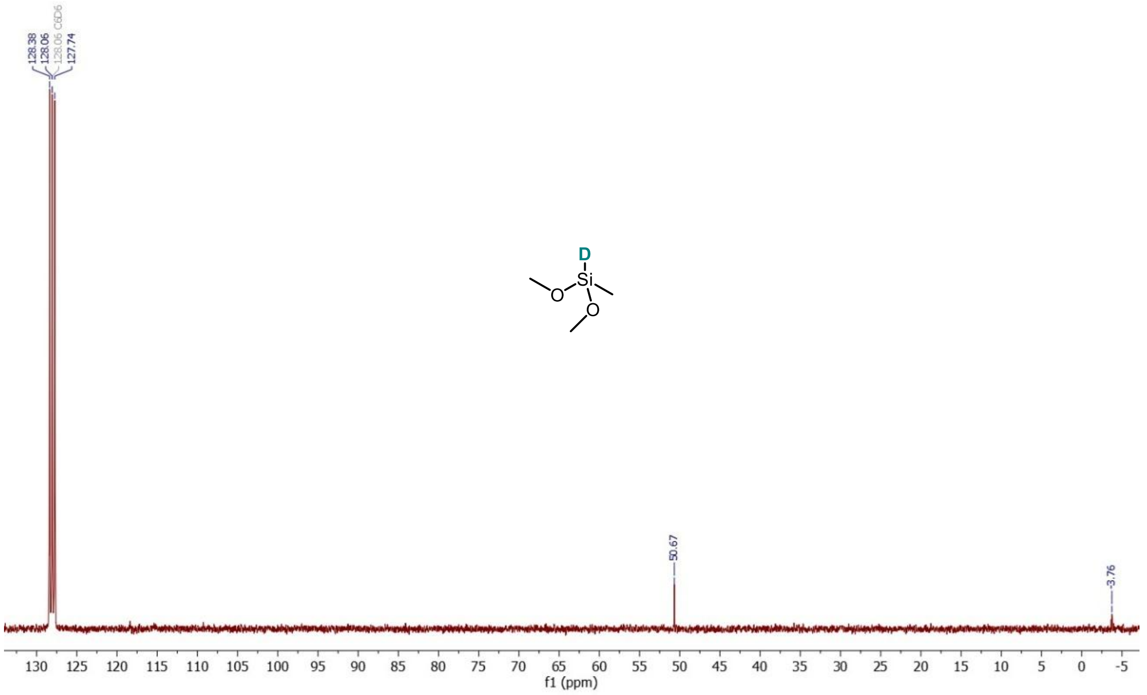

Figure S11. ^{1}H NMR spectrum (400 MHz) (top) and $^{13}C\{^{1}H\}$ NMR spectrum (75 MHz) (below) of $(MeO)_2MeSiD$ (**2k**) and $(MeO)_2MeSiH$ (**1k**), (C_6D_6, 298K)

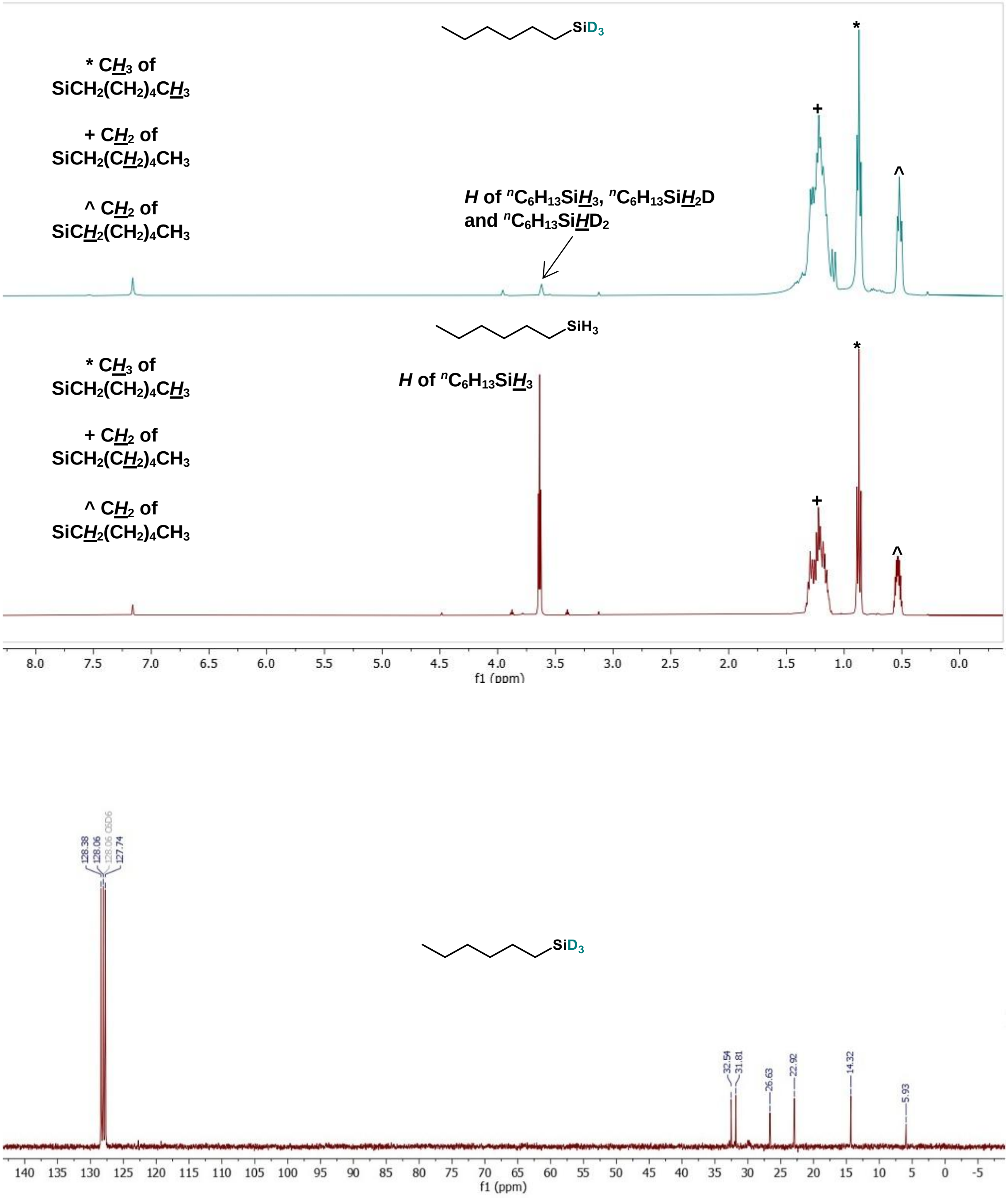

Figure S12. ^{1}H NMR spectrum (400 MHz) (top) and ^{13}C{^{1}H} NMR spectrum (75 MHz) (below) of $^{n}C_6H_{13}SiD_3$ (**2l**) and $^{n}C_6H_{13}SiH_3$ (**1l**), (C_6D_6, 298K)

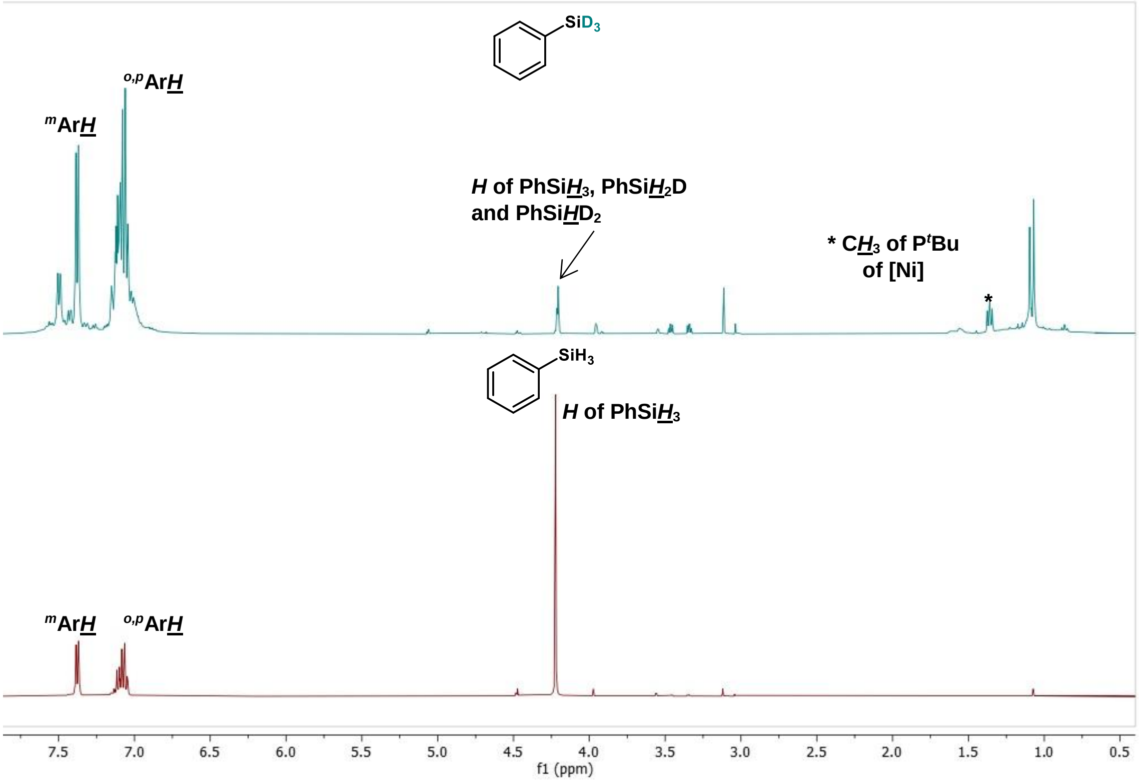

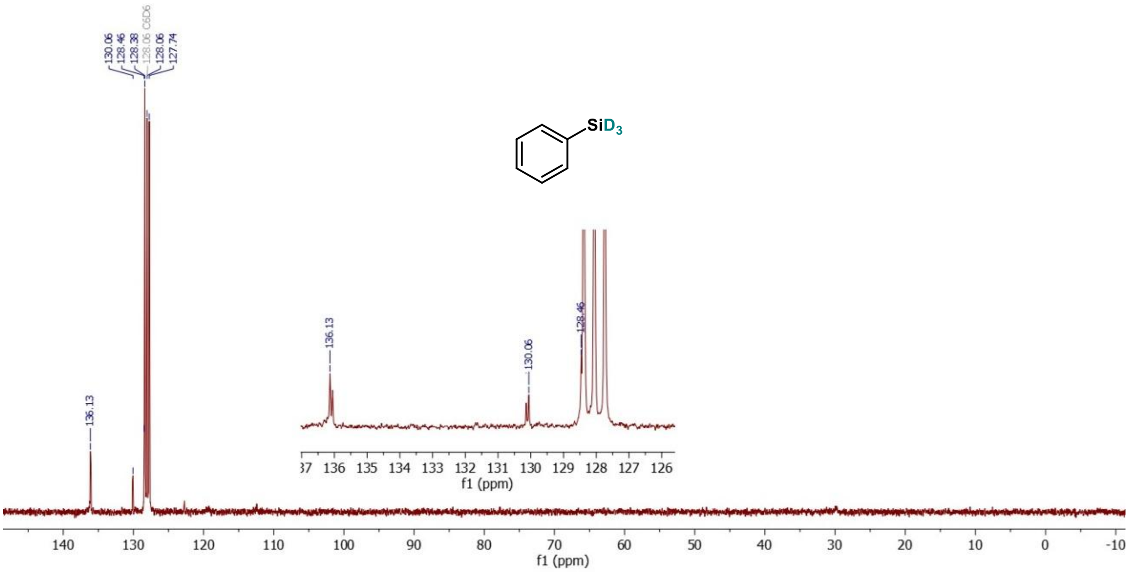

Figure S13. ^{1}H NMR spectrum (400 MHz) (top) and $^{13}C\{^{1}H\}$ NMR spectrum (75 MHz) (below) of $PhSiD_3$ (**2m**) and $PhSiH_3$ (**1m**), (C_6D_6, 298K)

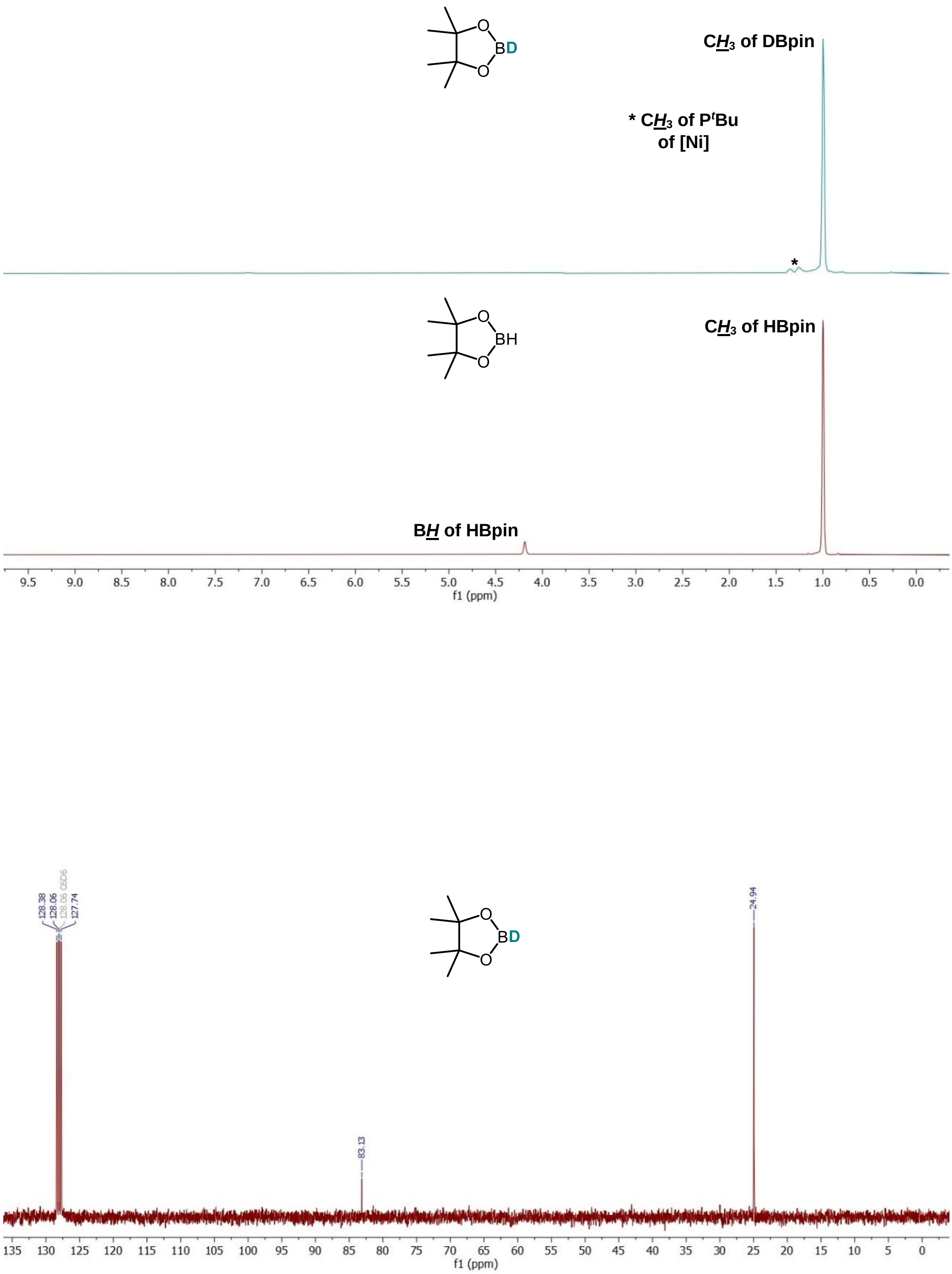

Figure S14. $^{1}H\{^{11}B\}$ NMR spectrum (400 MHz) (top) and $^{13}C\{^{1}H\}$ NMR spectrum (75 MHz) (below) of DBpin (**2p**) and HBpin (**1p**), (C_6D_6, 298K)

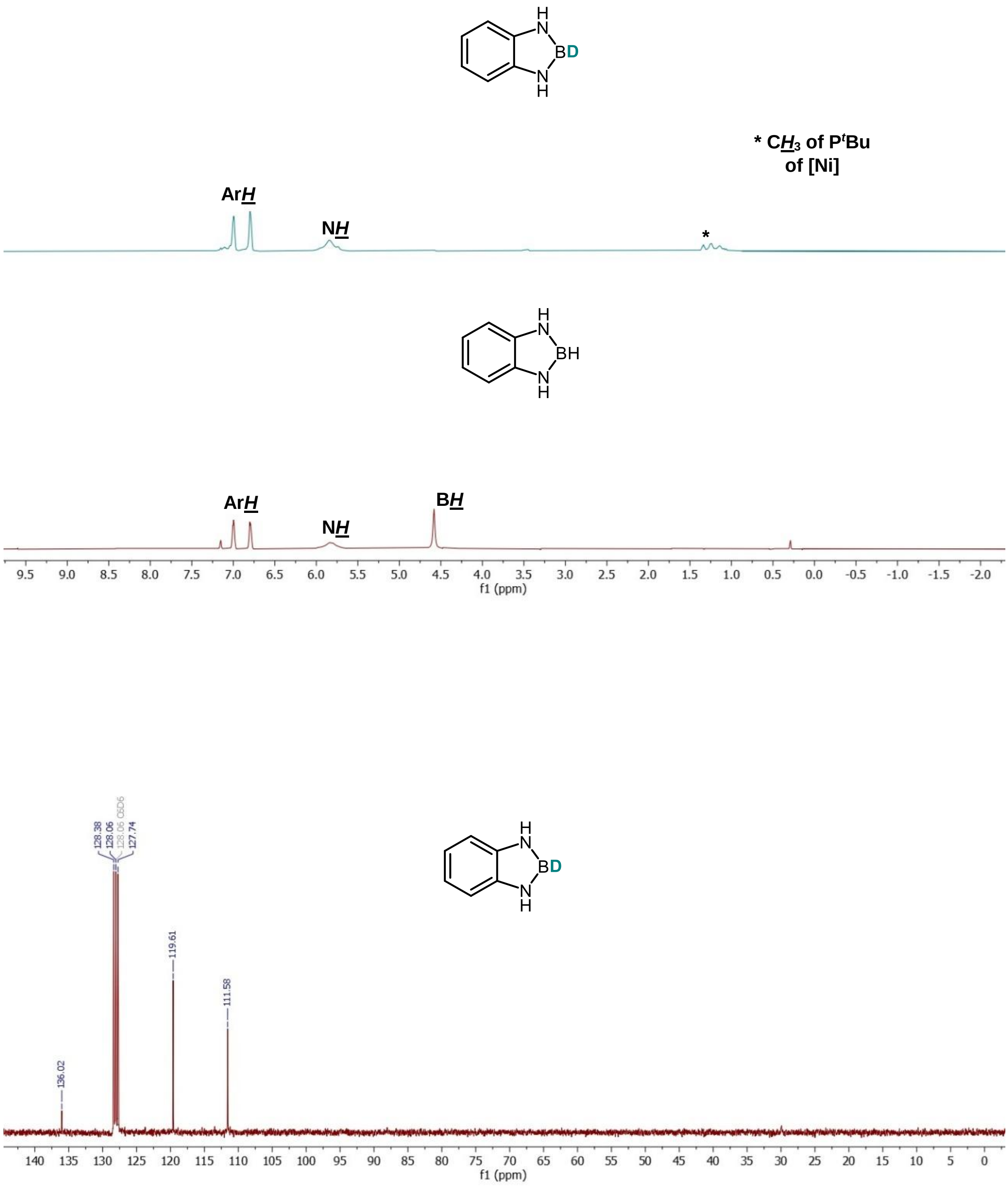

Figure S15. $^{1}H\{^{11}B\}$ NMR spectrum (400 MHz) (top) and $^{13}C\{^{1}H\}$ NMR spectrum (75 MHz) (below) of benzodiazoborane-D (**2q**) and benzodiazoborane-H (**1q**), (C_6D_6, 298K)

4. *In Situ* NMR Studies

NMR scale reactions using complex 5 under deuterium pressure.

The deuteration of Et_2SiH_2, Ph_2SiH_2 and $PhMeSiH_2$ under 2 or 4 bar of deuterium using (^{t}BuPBP)NiH (**5**) was monitored using *in situ* ^{1}H NMR spectroscopy in a J. Young valve NMR tube. The resonances of complex **5** remain observable during the reaction. After 19h at room temperature, almost complete consumption of the Si–H peak is observed in the ^{1}H NMR spectrum under 2 bar of D_2 (Figures **S16** and **S17**). On the other hand, the deuteration of these silanes under 4 bar of D_2 (Figures **S18**, **S19 and S22**) achieve better yields. Nevertheless, second loadings of D_2 didn't lead to full deuterium incorporation (**Figure S20** and **S21**). Finally, optimal deuterium incorporation is achieved when filling a Fischer-Porter vessel which contain complex **5** and the corresponding silane with D_2 (2 bar) using $MePh_2SiH$ or $MePhSiH_2$ as examples (Table S7).

Table S2. Control experiments using **5** (5 mol %) and D_2 (2 bar) at room temperature.

Entry	Time	Et_2SiD_2	Ph_2SiD_2
1	30 min	11%	20%
2	2h	30%	43%
3	4h	49%	57%
4	19h	72%	70%

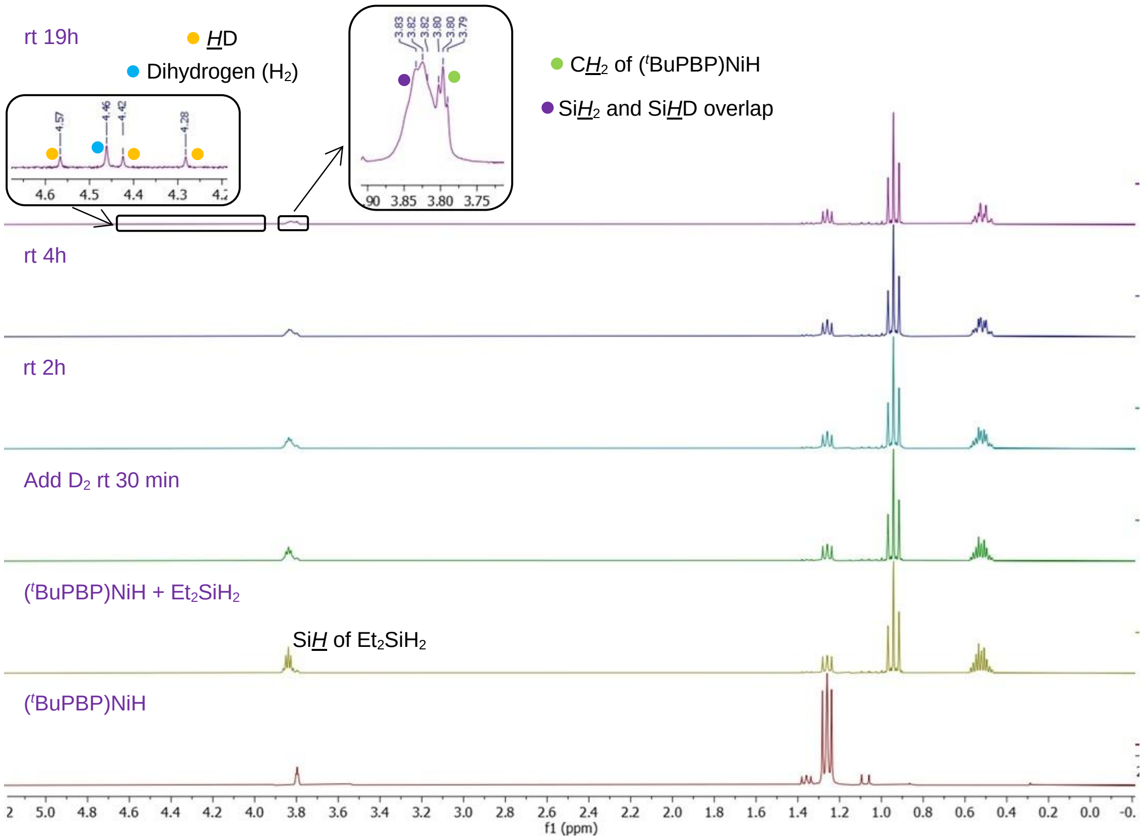

Figure S16. ^{1}H NMR spectrum of deuteration (2 bar) of Et_2SiH_2 using complex **5** in a J. Young NMR tube (300 MHz, C_6D_6, 298K)

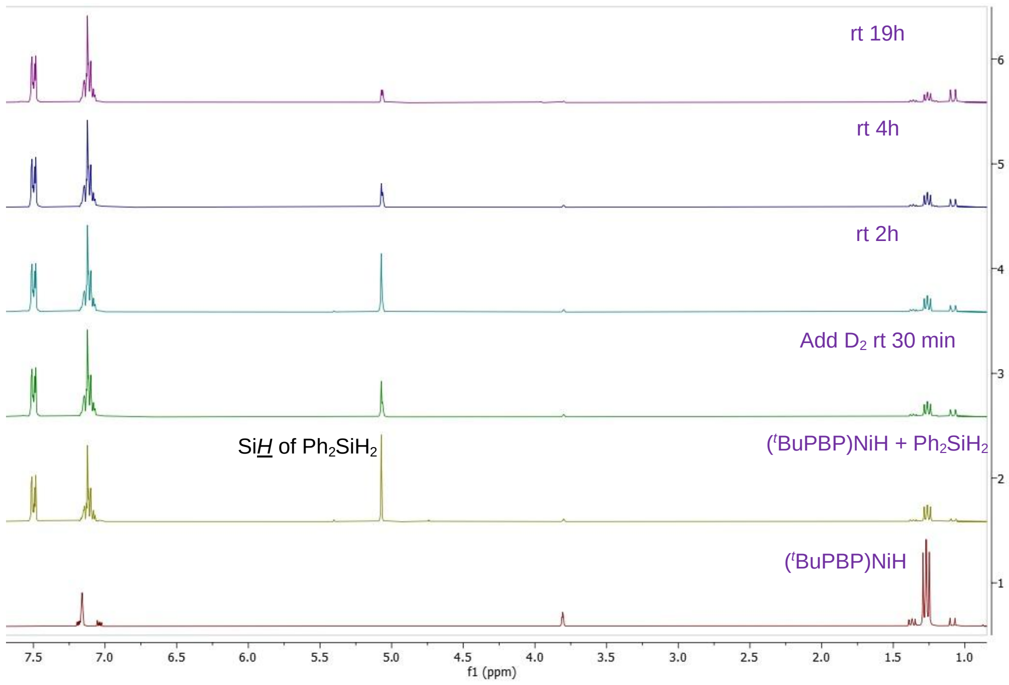

Figure S17. ^{1}H NMR spectrum of deuteration (2 bar) of Ph_2SiH_2 using complex **5** in a J. Young NMR tube (300 MHz, C_6D_6, 298K)

Table S3. Control experiments using **5** (5 mol %) and D_2 (4 bar) at room temperature.

Entry	Time	Et_2SiD_2	Ph_2SiD_2
1	2h	16%	32%
2	4h	47%	49%
3	6h	54%	55%
4	19h	83%	79%

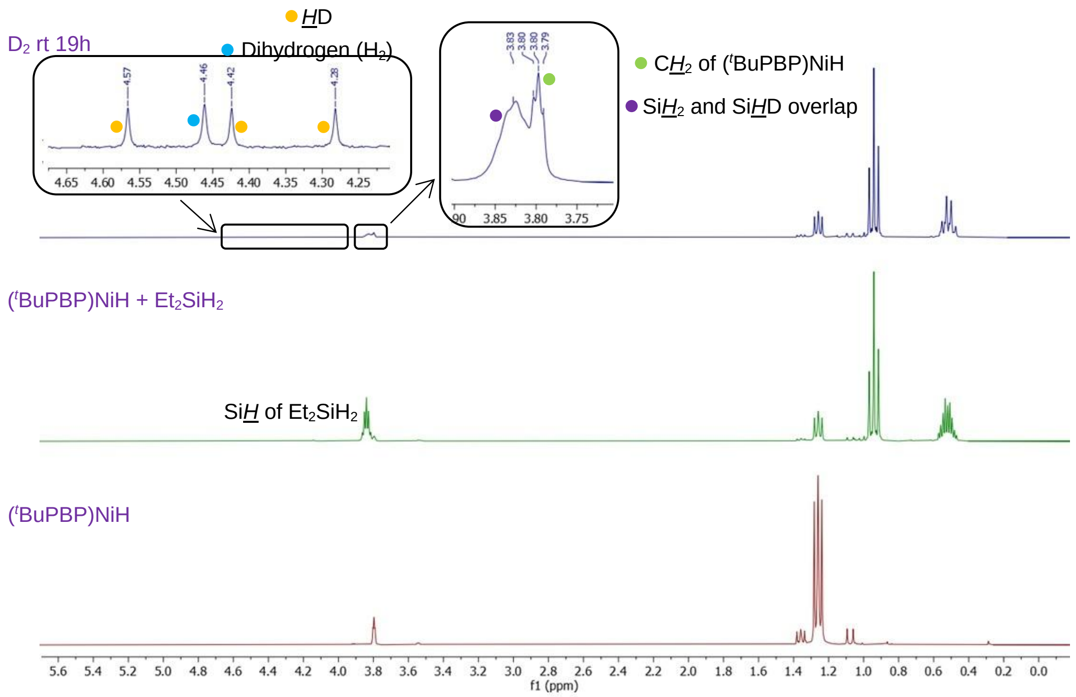

Figure S18. ^{1}H NMR spectrum of deuteration (4 bar) of Et_2SiH_2 using complex **5** in a J. Young NMR tube (300 MHz, C_6D_6, 298K)

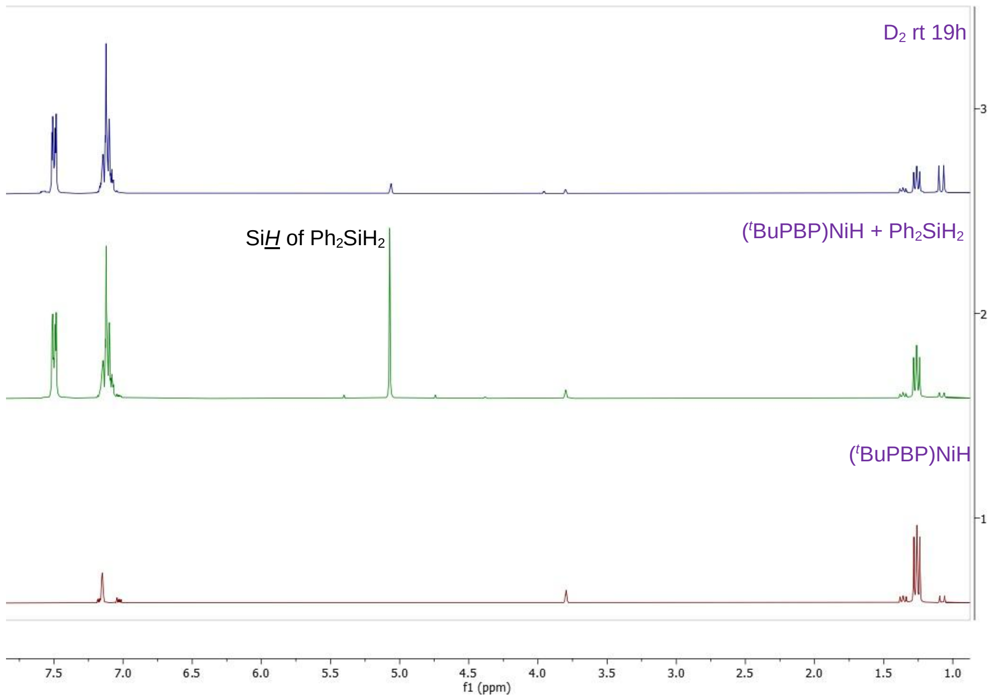

Figure S19. ^{1}H NMR spectrum of deuteration (4 bar) of Ph_2SiH_2 using complex **5** in a J. Young NMR tube (300 MHz, C_6D_6, 298K)

Table S4. Control experiments of deuteration of Et_2SiH_2 using **5** (1 mol %) and D_2 (4 bar) at room temperature.

Entry	D_2	Time[a]	Et_2SiD_2
1	Filled 4 bar	0	50%
2		13h	80%
3		18h	80%
4	Refilled 4 bar	0	84%
5		2h	85%

[a] After 18h, refilled with D_2 (4 bar) and stirred for a further 2h.

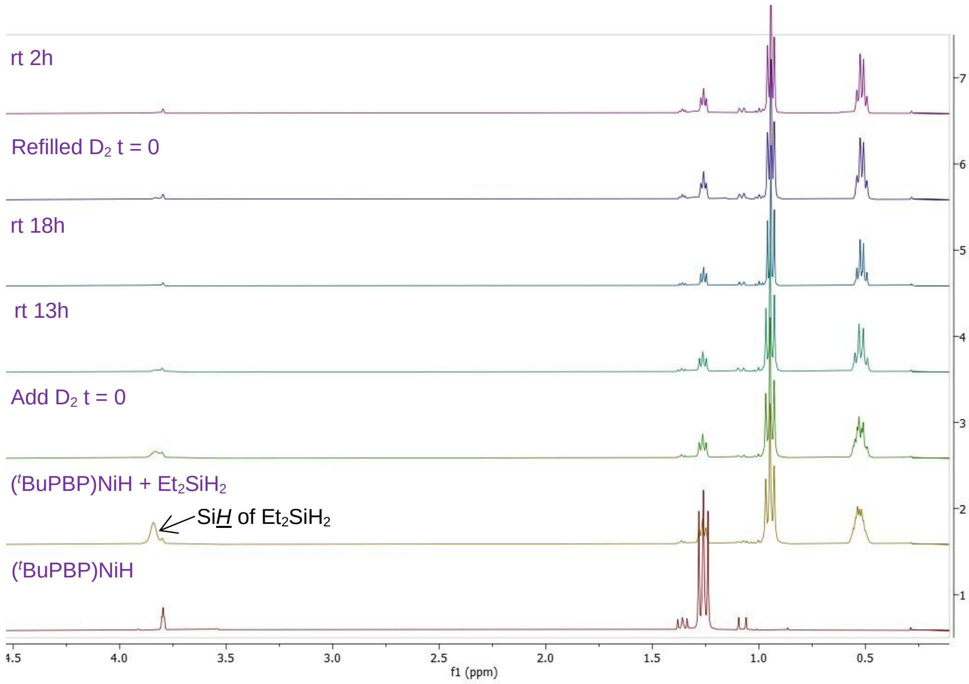

Figure S20. ^{1}H NMR spectrum of deuteration (4 bar) of Et_2SiH_2 using complex **5** (400 MHz, C_6D_6, 298K)

Table S5. Control experiments of deuteration of Ph_2SiH_2 using **5** (1 mol %) and D_2 (4 bar) at room temperature.

Entry	D_2	Time[a]	Ph_2SiD_2
1	Filled 4 bar	0	38%
2		13h	81%
3		18h	81%
4	Refilled 4 bar	0	85%
5		2h	86%

[a] After 18h, refilled with D_2 (4 bar) and stirred for a further 2h

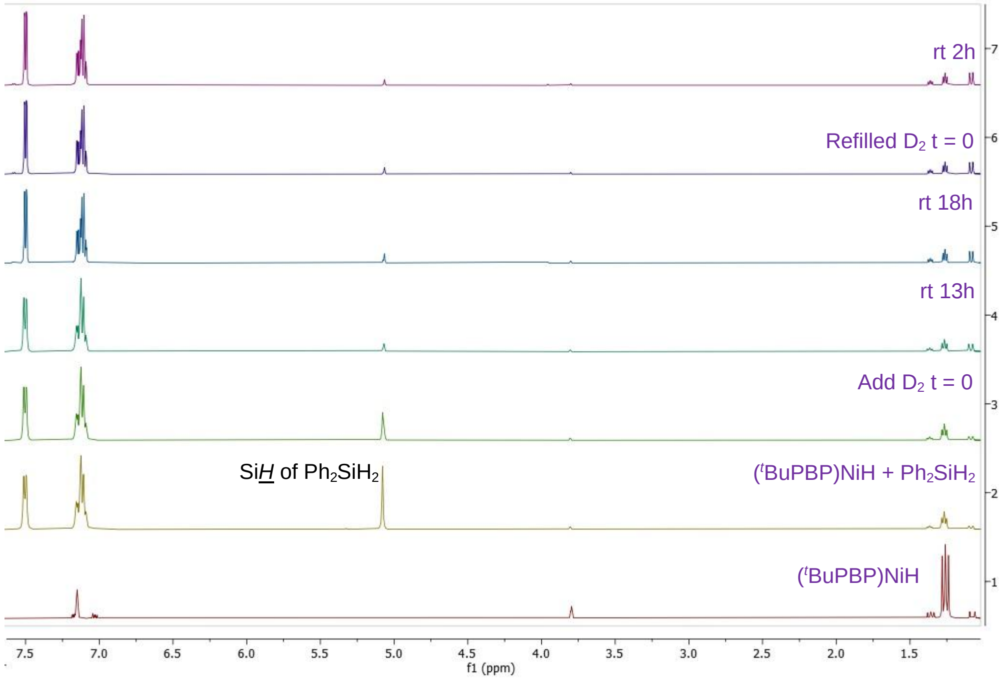

Figure S21. ^{1}H NMR spectrum of deuteration (4 bar) of Ph_2SiH_2 using complex **5** (400 MHz, C_6D_6, 298K)

Table S6. Control experiments of deuteration of $PhMeSiH_2$ using **5** (1 mol %) and D_2 (4 bar) at room temperature.

Entry	D_2	Time[a]	$PhMeSiD_2$
1	Filled 4 bar	0	36%
2		13h	79%
3		18h	80%
4	Refilled 4 bar	0	85%
5		2h	88%

[a] After 18h, refilled with D_2 (4 bar) and stirred for a further 2h

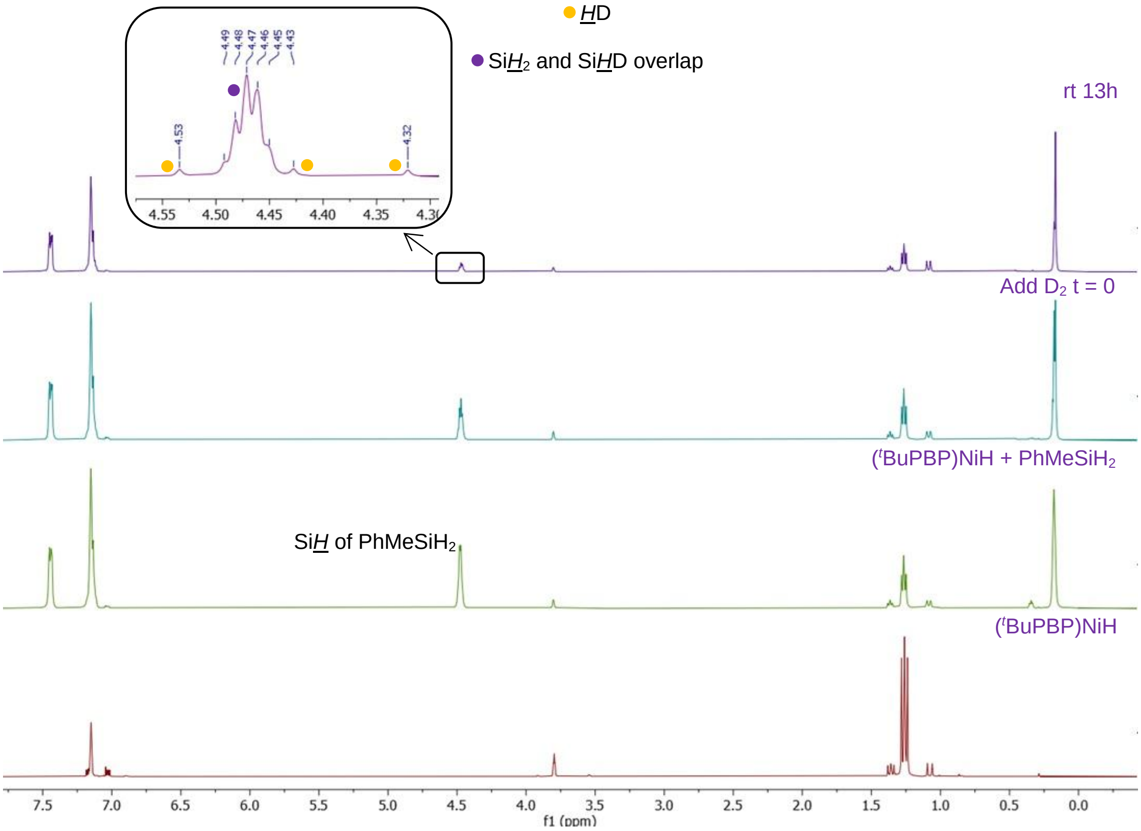

Figure S22. ^{1}H NMR spectrum of deuteration (4 bar) of $PhMeSiH_2$ using complex **5** in a J. Young NMR tube (400 MHz, C_6D_6, 298K)

Table S7. Control experiments of deuteration of $MePh_2SiH$ or $PhMeSiH_2$ using **5** and D_2 at room temperature in a Fischer-Porter vessel.[a]

Entry	[Ni]	D_2	Time	Silane	$MePh_2SiD$/$PhMeSiD_2$
1	5 mol %	3 bar	4h	$MePh_2SiH$	92%
2	2.5 mol %	3 bar	4h	$MePh_2SiH$	93%
3	1 mol %	3 bar	4h	$MePh_2SiH$	92%
4	2.5 mol %	2 bar	4h	$PhMeSiH_2$	85%
5	2 mol %	2 bar	4h	$PhMeSiH_2$	84%
6	1 mol %	2 bar	4h	$PhMeSiH_2$	72%
7	1 mol %	1 bar	4h	$PhMeSiH_2$	50%
8[b]	2 mol %	2 bar	4h	$PhMeSiH_2$	94%

[a] Conditions: 6 mL Fischer-Porter tube containing silane (0.08 mmol), catalyst (n mol %), D_2, C_6D_6 (0.4 mL), rt, 4h. [b] Conditions: 20 mL Fischer-Porter tube containing silane (0.08 mmol), catalyst (2 mol %), D_2 (2 bar), C_6D_6 (0.4 mL), rt, 4h.

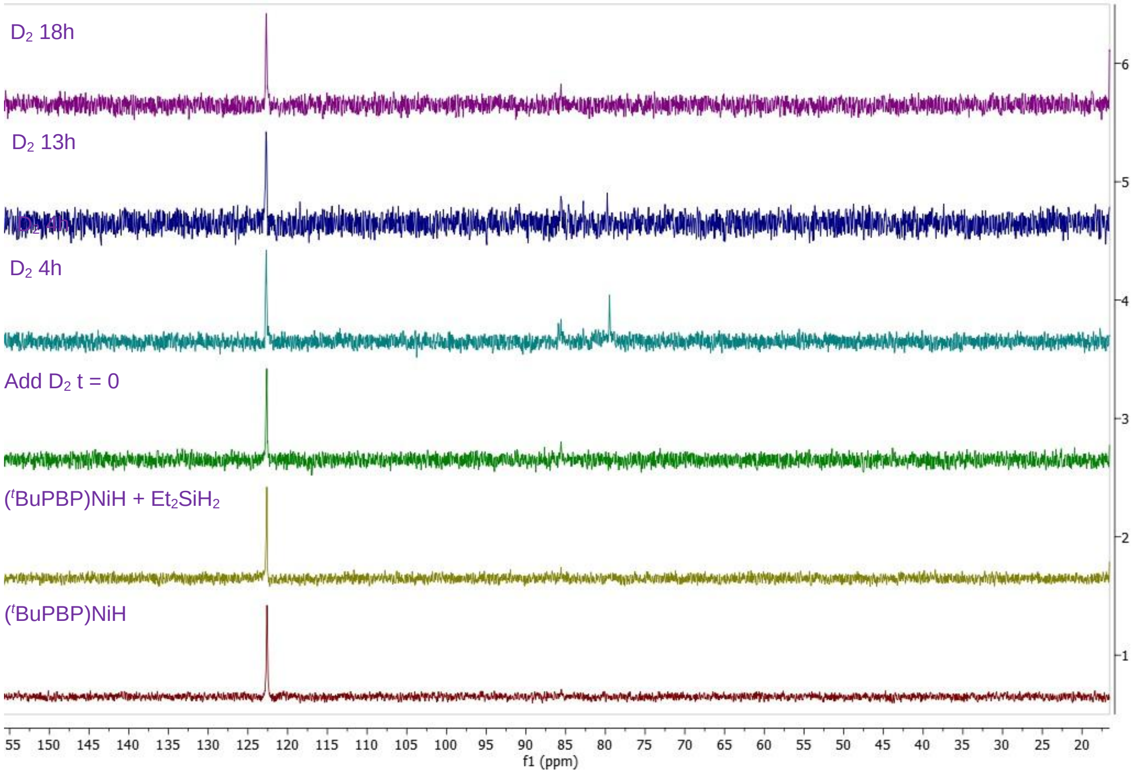

Figure S23. $^{31}P\{^{1}H\}$ NMR spectrum from complex **5** (1 mol %) in a J. Young NMR tube (400 MHz, C_6D_6, 298K) for the H/D exchange in Et_2SiH_2 using 2 bar of D_2.

5. Kinetics studies

5.1- General considerations. Kinetic experiments focused on the catalytic deuteration of $PhMeSiH_2$ in C_6D_6 were performed in NMR tubes equipped with J. Young valves, with an initial D_2 pressure of 2 bar. Data were analyzed considering the following assumptions:

a) the reaction takes place following the stoichiometry of equation 1, *i.e.* converting D_2 and the substrate into methylphenylsilane-*d*$_2$ with liberation of H_2:

$$PhMeSiH_2 + D_2 \xrightleftharpoons{\textbf{5}\text{ (cat.)}} PhMeD_2 + H_2 \qquad (\textbf{1})$$

b) as the reaction rate is calculated form the consumption of $PhMeSiH_2$, the presence of the monodeuterated species (detected in low concentrations in same cases), is not considered relevant in the kinetic study of reaction 1;

c) although reaction 1 is reversible with a corresponding ΔG^0 close to 0 kcal/mol, the kinetic contribution of the hydrogenation reaction (eq. 1, from right to left) can reasonably be omitted within the rate law, provided that data collection and analysis are performed for conversions of $PhMeSiH_2$ up to 30-40 %;[1]

d) since the reaction takes place in a closed system under isothermal conditions, the concentration of D_2 decreases linearly with [$PhMeSiH_2$] (eq. 1), both in solution and in the gas phase;

e) the overall reaction order is assumed to be 2, as suggested by theoretical calculations and qualitative observations, the latter showing significant effects on the reaction rate of both [D_2] and [$PhMeSiH_2$];

f) catalyst concentration, [*cat*] = [**5**], is assumed to be constant in each experiment, at least reaction times up to *ca.* 180 min, so that the observed rate constant (k_{obs}) will depend on [*cat*] as follows (eq. 2):

$$k_{obs} = k' \cdot [cat]^n \qquad (\textbf{2})$$

where n is the reaction order in the catalyst.

Rate law. Assuming that Henry's law is applicable under our experimental conditions and that the hydrogenation reaction rate is negligible (*vide supra*, point c), the concentration of D_2 in solution can be estimated as directly proportional to its pressure and, eventually, to the overall molar amount of D_2 in the system ($n_{D_2}^{tot}$). Hence, with partial orders of one for both reagents of eq. 1, the rate law at a temperature T can be written as follows (eq. 3, [$PhMeSiH_2$] will be denoted as x, from now on):

$$-\frac{dx(t)}{dt} = k(T) \cdot x(t) \cdot [\mathrm{D_2}](t) \approx c(T) \cdot k(T) \cdot x(t) \cdot n_{D_2}^{tot}(t) \qquad (\textbf{3})$$

[1] To corroborate the validity of this approximation, the rate law $-dx/dt = k_1 \cdot x \cdot [D_2] - k_{-1} \cdot (x_o - x) \cdot [H_2]$ was evaluated for $k_1 = k_{-1}$ (x_0 initial concentration of $PhMeSiH_2$), but no match with the experimental data was found. So, we sought a better approximation of the $k_1{:}k_{-1}$ ratio by means of the equilibrium constant of reaction 1, K_{eq}, which was estimated in the range 3-4 from a calculated ΔG^0 of –0.72 kcal/mol. On the ground that $K_{eq} = k_1/k_{-1}$, we concluded that, for conversions up to 30-40%, the hydrogenation contribution to the rate law can be ignored.

where k(T) and c(T) are the rate constant and a constant which expresses the direct proportionality of [D_2] to $n_{D_2}^{tot}$, respectively (both constants depends on the temperature, T). The overall molar amount of D_2, $n_{D_2}^{tot}(t)$ can easily be calculated as a function of $x(t)$ (eq. 4):

$$n_{D_2}^{tot}(t) = n_{D_2}^{0} - n_{PhMeSiH_2}^{0} + x(t) \cdot V \qquad \textbf{(4)}$$

where $n_{\mathrm{PhMeSiH_2}}^{0}$, $n_{\mathrm{D_2}}^{0}$, and V are the initial molar amounts of $PhMeSiH_2$ and D_2, and the volume of the C_6D_6 solution in which the reaction is conducted, respectively.

Combining equations 3 and 4, the rate equation can be written as follows (eq. 5):

$$-\frac{dx(t)}{dt} \approx k_{obs} \cdot x(t) \cdot [x(t) + \alpha] \qquad \textbf{(5)}$$

where $k_{obs} = c\,k\,V$ and $\alpha = (n_{\mathrm{D_2}}^{0} - n_{\mathrm{PhMeSiH_2}}^{0})/V$.

Once the differential equation 5 is integrated, the following rate law is obtained (eq. 6):

$$ln\frac{x(t)}{x(t)+\alpha} = ln\frac{x_0}{x_0+\alpha} - \alpha \cdot k_{obs} \cdot t \qquad \textbf{(6)}$$

For each experiment, $ln\frac{x(t)}{x(t)+\alpha}$ values were plotted as a function of time and examined with linear regression algorithms. The resulting R^2 values permitted to evaluate the goodness-of-fit of the experimental data to the proposed kinetic model, whereas the slopes of the corresponding trendlines were taken as the values of $-\alpha \cdot k_{obs}$ (eq. 6). Moreover, each set of experiments was repeated with different molar amounts of catalyst, to evaluate the reaction order in catalyst concentration ([*cat*]/*x* = 6, 8, 10, 12, and 14%). In particular, if n = 1 (see eq. 2), *i.e.* order one in catalyst concentration, k_{obs} will linearly depend on [*cat*] and plotting k_{obs} *vs* [*cat*] will match with a straight line.

<u>5.2- Experimental procedure</u>.

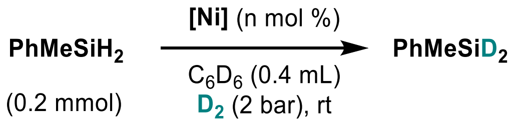

A J. Young tube valve NMR was charged with catalyst **5** (6, 8, 10, 12 or 14 mol %), $PhMeSiH_2$ (0.2 mmol) and mesitylene (0.2 mmol) in C_6D_6 (400 μL) and was degassed via three freeze-pump-thaw cycles. Then, the solution was backfilled with D_2 gas (2 bar) and inserted in an NMR apparatus at 25ºC. The progression of the reaction was monitored by ^{1}H NMR using the following parameters for each spectrum; d1 = 10, ns = 4. All experiments were run in duplicate.

Table S8. Concentration values for each component used to determine the order of (^{tBu}PBP)-NiH in the reaction rate.

Entry	[$PhMeSiH_2$]	D_2	[$^{(tBu)}$PBP-NiH]
1	0.5 mM	2 bar	0.03 mM
2	0.5 mM	2 bar	0.04 mM
3	0.5 mM	2 bar	0.05 mM
4	0.5 mM	2 bar	0.06 mM

5.3- Results.

Figure S23-S27 show the plots of the values of the expression calculated every 300 s from the experimental values of *x* (Table S9). Equations of the trend-lines inferred by linear regression algorithms, along with the corresponding R^2 values, are also shown. Each graph refers to a specific amount of catalyst, as indicated in the respective figure captions. The goodness-of-fit of the kinetic model to our set of observations has proved to be clearly satisfactory, being the R^2 values higher than 99% in all cases. The values of k_{obs} (with the corresponding standard deviations) are listed in table Table S10 and plotted over the corresponding catalyst concentrations in Figure S28. Again, a satisfactory linear dependence is found by applying linear regression algorithms, which provided a R^2 of 99.5%. In summary, these results are in excellent agreement with partial reaction orders of one for [$PhMeSiH_2$], [D_2], and [**5**] for the deuteration reaction shown in equation 1.

Table S9. Residual concentration[a] of [$PhMeSiH_2$] over time.

Time (s)	[$PhMeSiH_2$] (M) ([**5**] = 0.03 M)	[$PhMeSiH_2$] (M) ([**5**] = 0.04 M)	[$PhMeSiH_2$] (M) ([**5**] = 0.05 M)	[$PhMeSiH_2$] (M) ([**5**] = 0.06 M)	[$PhMeSiH_2$] (M) ([**5**] = 0.07 M)
600	0.406	0.372	0.341	0.324	0.298
900	0.396	0.364	0.332	0.315	0.285
1200	0.386	0.355	0.324	0.307	0.278
1500	0.377	0.347	0.316	0.298	0.270
1800	0.368	0.340	0.309	0.290	0.262
2100	0.359	0.332	0.301	0.283	0.255
2400	0.352	0.324	0.294	- [c]	- [c]
2700	0.344	0.317	0.287	-	-

[a] average values
[b] initial concentration of $PhMeSiH_2$ 0.5 M, catalyst load specified at the top of each column.
[c] See p. S20, point (c).

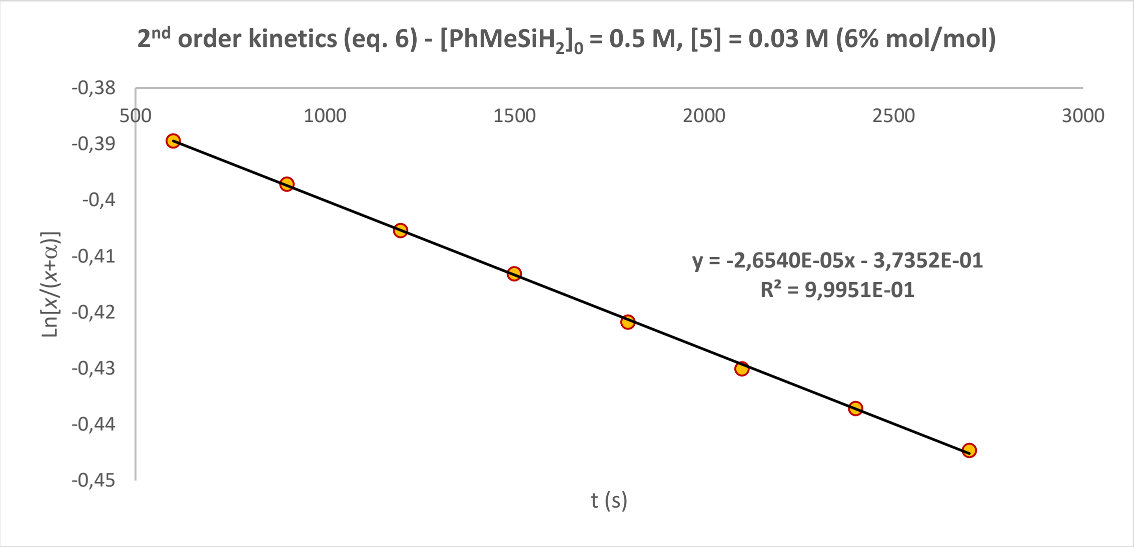

Figure S24. Catalytic deuteration of $PhMeSiH_2$ with a 6% catalyst load and initial concentration of $PhMeSiH_2$ 0.5 M. Variation of *Ln*[*x*/(*x*+α)] over time (α = 0.193 M, $n_{D_2}^0$ = 0.123 mmol).

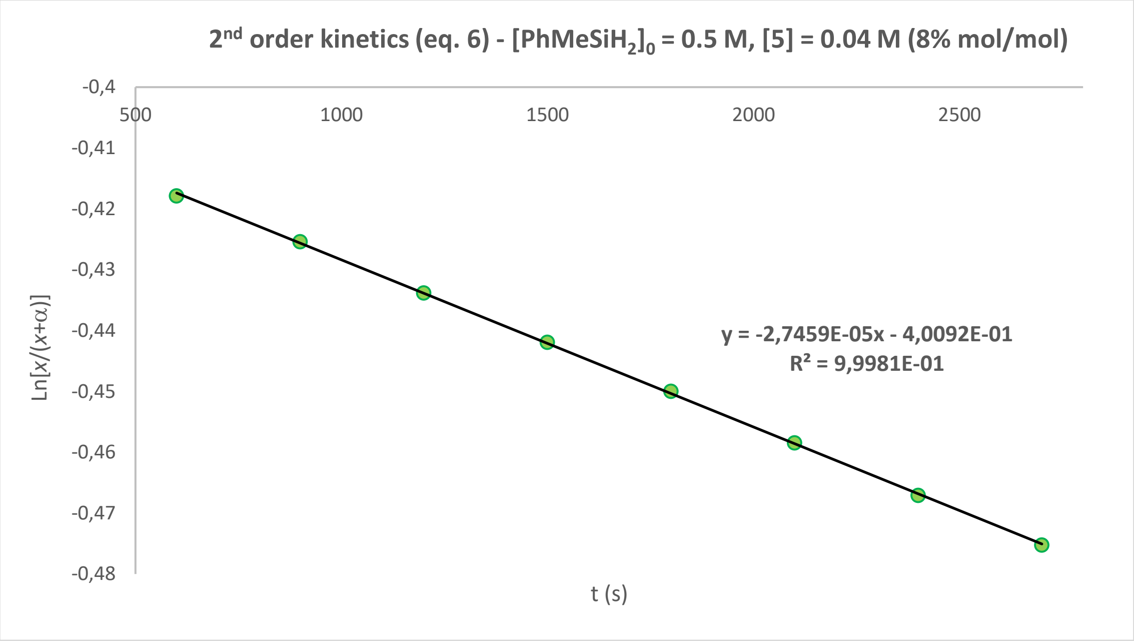

Figure S25. Catalytic deuteration of $PhMeSiH_2$ with an 8% catalyst load and initial concentration of $PhMeSiH_2$ 0.5 M. Variation of *Ln*[*x*/(*x*+α)] over time (α = 0.193 M, $n_{D_2}^0$ = 0.123 mmol).

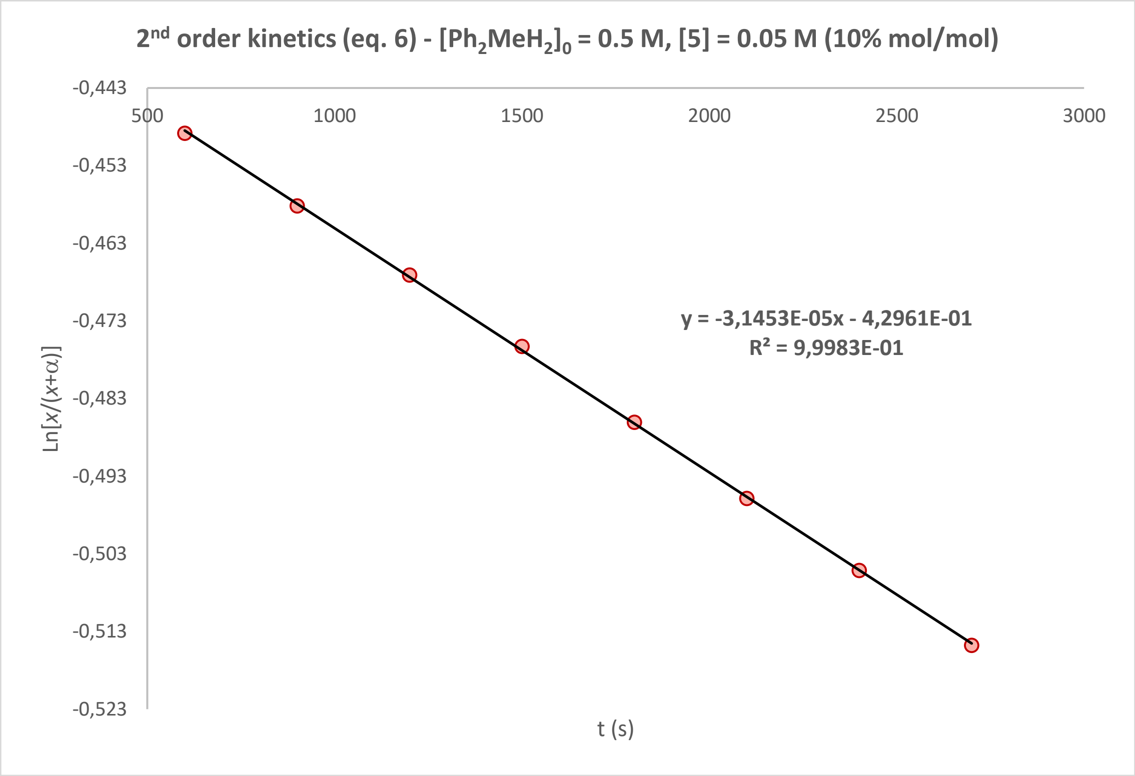

Figure S26. Catalytic deuteration of $PhMeSiH_2$ with a 10% catalyst load and initial concentration of $PhMeSiH_2$ 0.5 M. Variation of *Ln*[*x*/(*x*+α)] over time (α = 0.193 M, $n^0_{D_2}$ = 0.123 mmol).

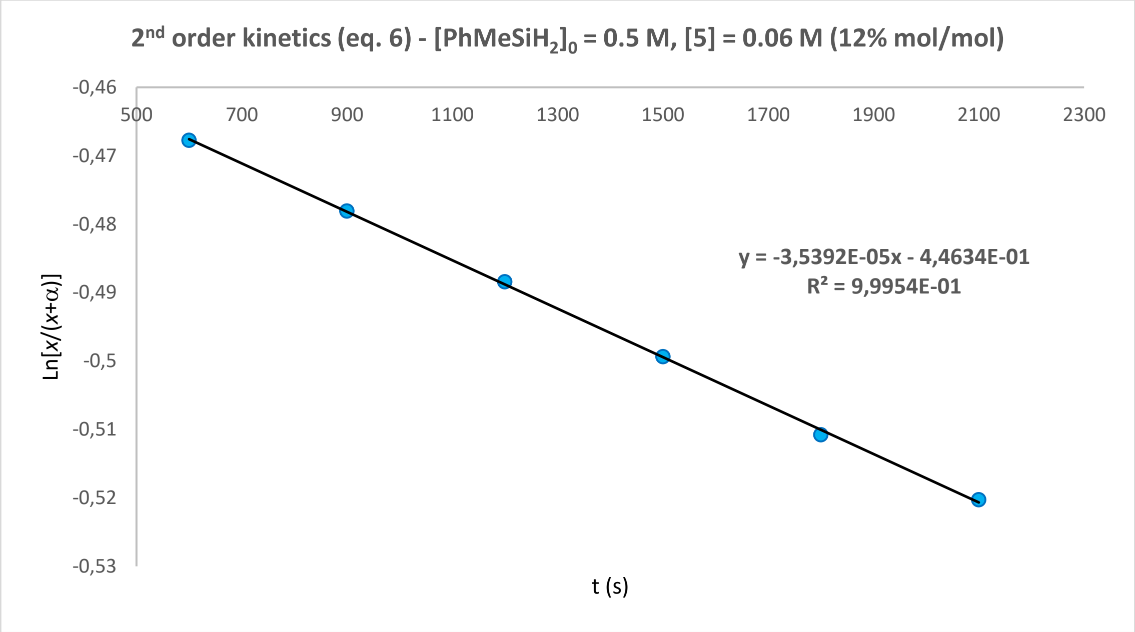

Figure S27. Catalytic deuteration of $PhMeSiH_2$ with a 12% catalyst load and initial concentration of $PhMeSiH_2$ 0.5 M. Variation of *Ln*[*x*/(*x*+α)] over time (α = 0.193 M, $n^0_{D_2}$ = 0.123 mmol).

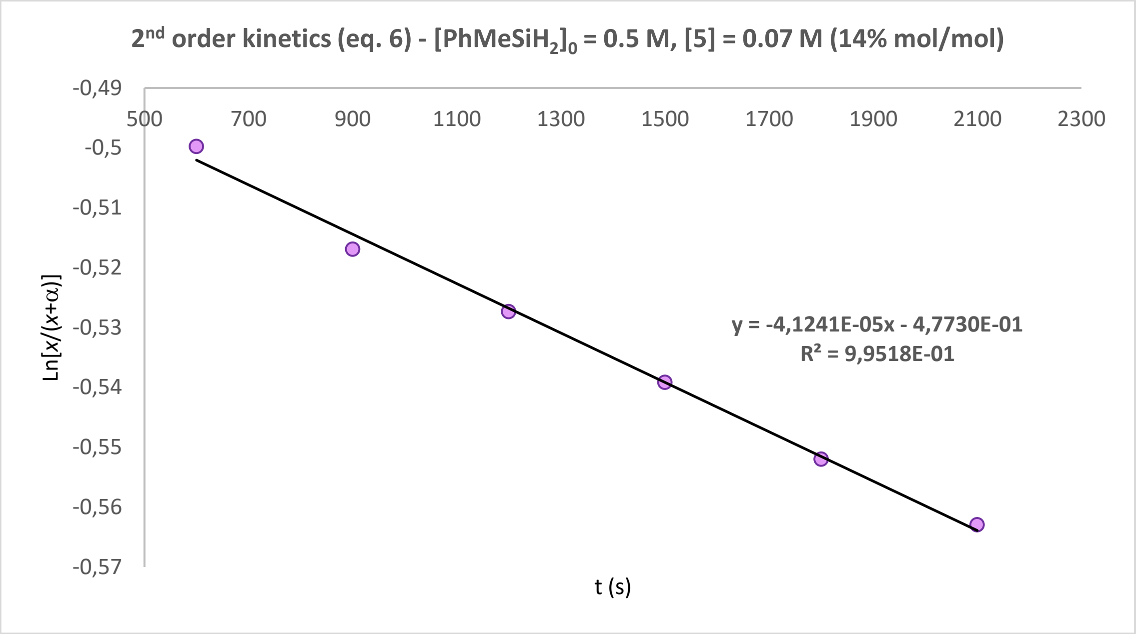

Figure S28. Catalytic deuteration of $PhMeSiH_2$ with a 14% catalyst load and initial concentration of $PhMeSiH_2$ 0.5 M. Variation of *Ln*[*x*/(*x*+α)] over time (α = 0.193 M, $n^0_{D_2}$= 0.123 mmol).

Table S10. k_{obs} values calculated for different catalyst concentrations ([**5**]).

x_0 (M)	[*cat*] (M)	k_{obs} ($M^{-1}s^{-1}$)/10^{-5}
0.5	0.03	12.2 ± 0.2
0.5	0.04	14.2 ± 0.2
0.5	0.05	16.3 ± 0.01
0.5	0.06	18.3 ± 0.2
0.5	0.07	21 ± 1

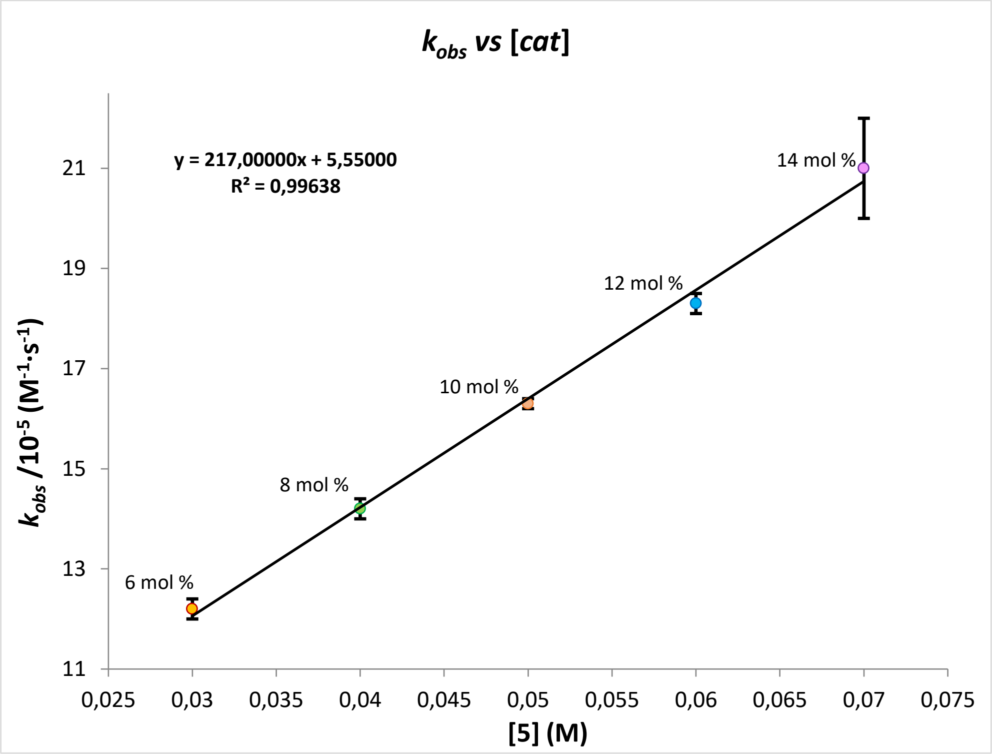

Figure S29. Catalytic deuteration of $PhMeSiH_2$. Variation of k_{obs} *vs* [**5**].

The order in catalyst [**5**] was confirmed visually by Variable Time Normalization Analysis (VTNA) as well.

The concentration plots obtained by NMR (Figure S29) show the concentration of $PhMeSiD_2$ against a normalized time scale to a zeroth- (top left), 0.5th (top right), first- (bottom left) and second- (bottom right) order dependence on the initial catalyst concentration, according to the method developed by Burés.[2] This analysis indicates that the reaction is first-order with respect to the catalyst (bottom left).

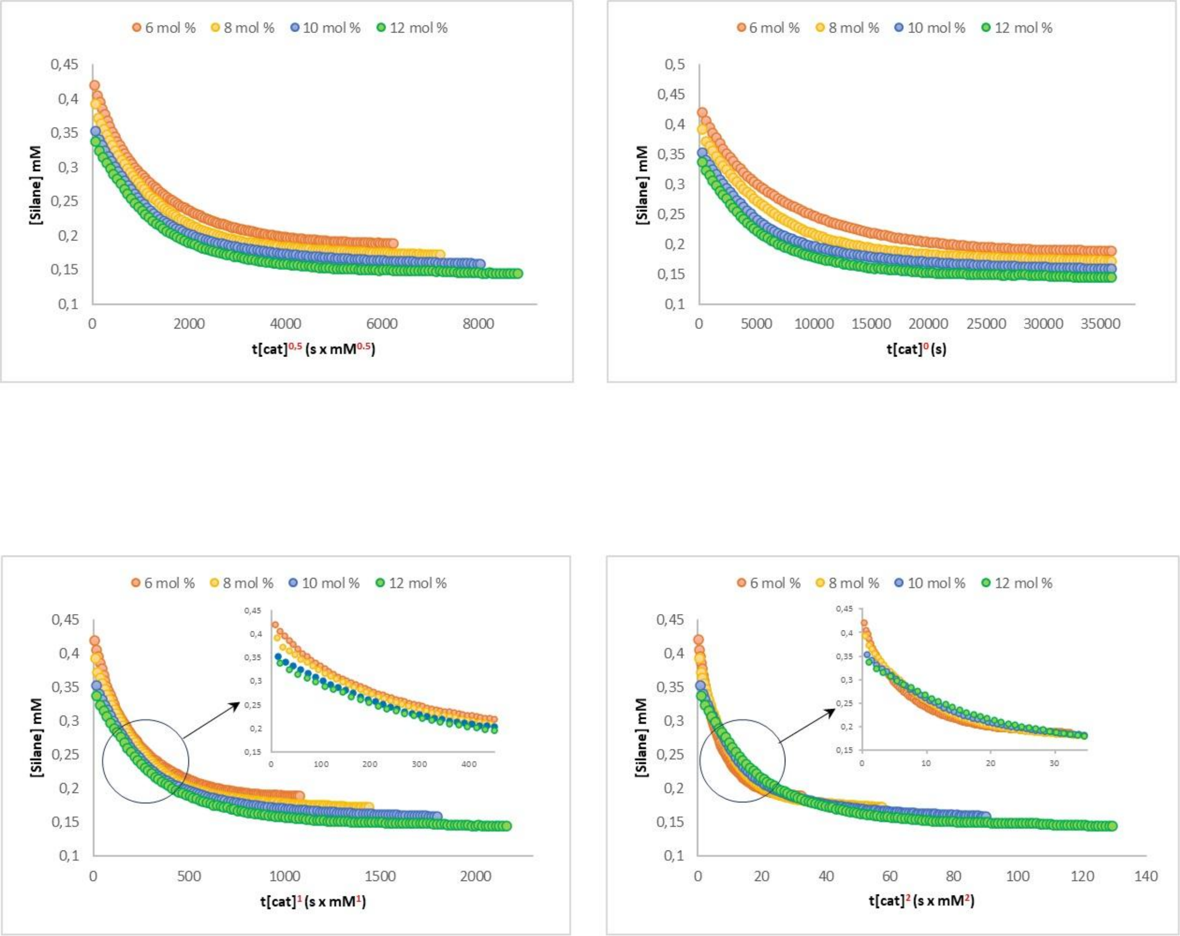

Figure S30. Plots obtained of the NMR data where the initial concentration of [$^{(tBu)}$PBP-NiH] (**5**) is elevated to different exponents.

5.4 Variable temperature ^{1}H NMR study of reaction of [(^{tBu})PBP-NiH] (5) with D_2 in presence of $PhMeSiH_2$ and line-shape data analysis

A J. Young tube valve NMR was charged with catalyst **5** (0.004 mmol), $PhMeSiH_2$ (0.2 mmol) and mesitylene (0.2 mmol) in C_6D_6 (400 µL) and was degassed via three freeze-pump-thaw cycles. Then, the solution was backfilled with D_2 gas (2 bar) and inserted in an NMR apparatus at different temperatures (in the range 298–318 K; Figure S30). The progression of the reaction was monitored by ^{1}H NMR using the following parameters for each spectrum; d1 = 10, ns = 4. The line-shape analysis plot of a variable temperature ^{1}H NMR study provided the data listed in Table S11. The corresponding Eyring plot is (Figure S31) permitted to estimate the following activation energy: $\Delta G^{\#}$ (298.15 K) = (22.9 ± 0.03) kcal·mol^{-1}. All experiments were run in duplicate.

Table S11. k_{obs} values calculated at different temperatures ([**5**]).

T **(K)**	***1/T***	k_{obs} **(s^{-1}M^{-1})/ 10^{-5} (average ± Std. Dev.)**	***k'*** **(s^{-1}M^{-2})/ 10^{-3} (average ± Std. Dev.)**	***Ln(k'/T)***
288	3.47E-03	6.7 ± 0.2	6.7 ± 0.2	-10.7 ± 0.1
298	3.36E-03	9.4 ± 0.2	9.4 ± 0.2	-10.4 ± 0.1
308	3.25E-03	13.0 ± 0.4	13.0 ± 0.4	-10.1 ± 0.1

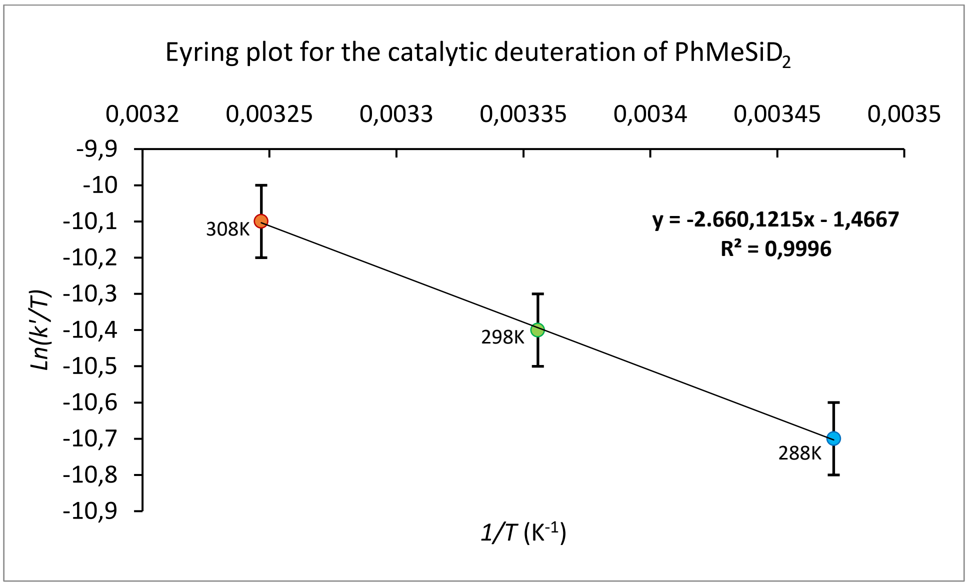

$\Delta H^‡$ = 5.25 (± 0.04) kcal·mol^{-1} $\Delta S^‡$ = –50.2 (± 0.6) cal·mol^{-1} K^{-1} $\Delta G^‡$ (298.15) = 20.2 (± 0.03) kcal·mol^{-1}

Figure S31. Eyring plot and activation parameters for the reaction of catalyst **5** with D_2 (2 bar) and $PhMeSiH_2$. Each data point is the average of two experiments with standard deviation of the mean included as error bars.

6. Reactivity studies of complex 5 with $ClMe_2SiH$ and HBcat

6.1 Experimental procedure and NMR spectrums of the reaction of complex 5 with $ClMe_2SiH$

In a globe box, a J. Young tube valve NMR was charged with catalyst **5** (0.010 mmol) and $ClMe_2SiH$ (0.012 mmol) in C_6D_6 (400 µL) at room temperature. After 30 min at room temperature, complete consumption of **5** is observed in the $^{31}P\{^1H\}$ and ^1H NMR spectrums to lead the complex **6**.

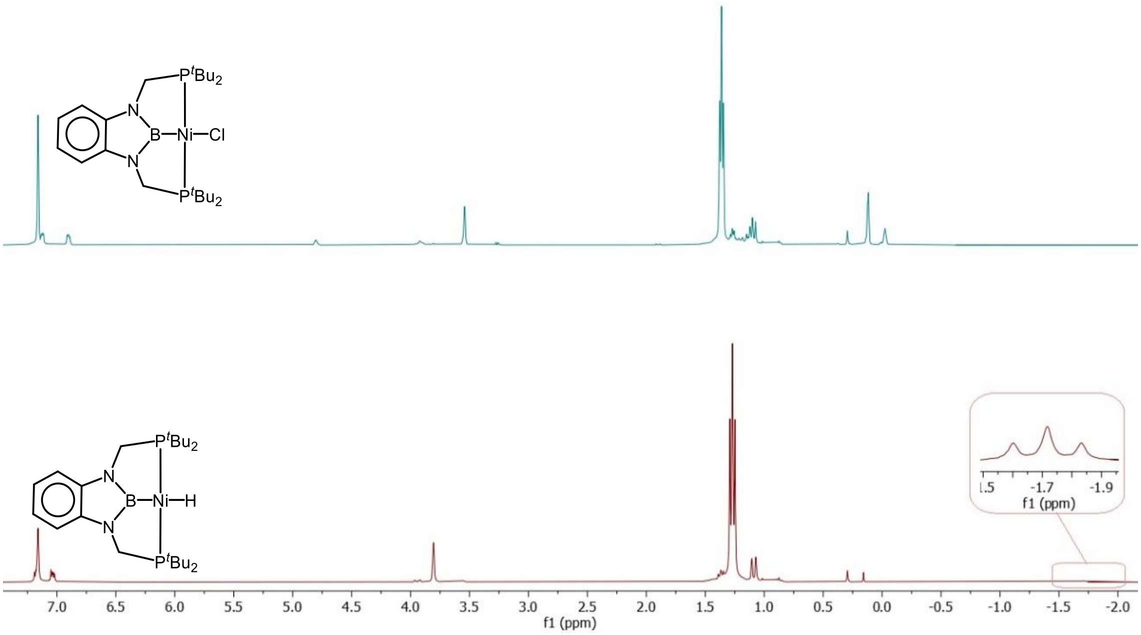

Figure S32. ^{1}H NMR spectra (C_6D_6 at 298 K) of complex **5** (bottom) and ^{1}H NMR spectra after addition of $ClMe_2SiH$ to give complex **6** (top).

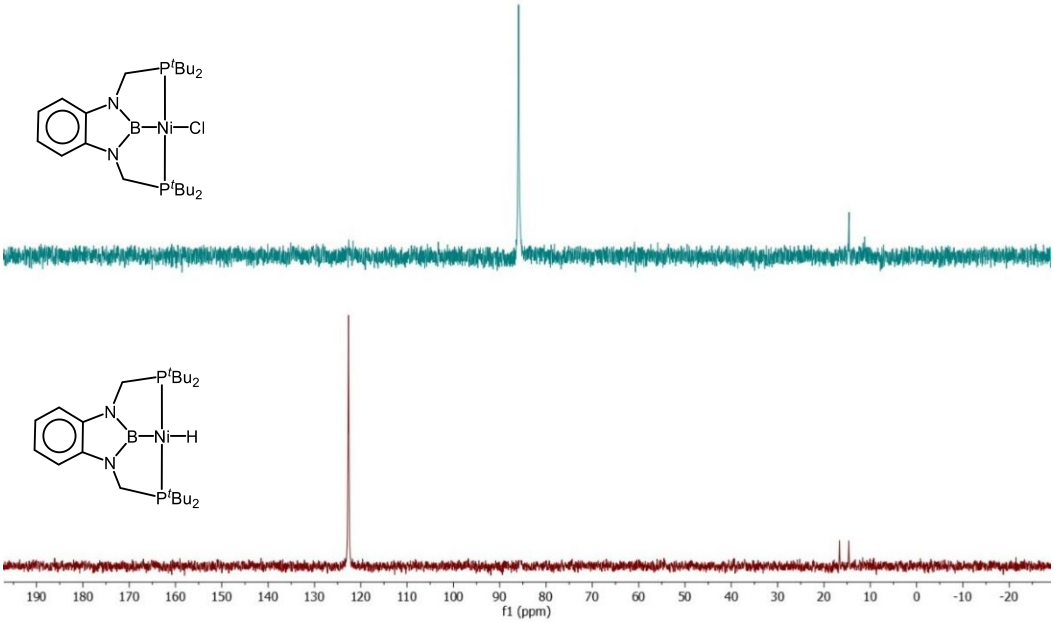

Figure S33. $^{31}P\{^1H\}$ NMR spectra (C_6D_6 at 298 K) of complex **5** (bottom)and $^{31}P\{^1H\}$ NMR spectra after addition of $ClMe_2SiH$ to give complex **6**.

6.2 Experimental procedure and NMR spectrums of the reaction of complex 5 with HBcat

In a globe box, a J. Young tube valve NMR was charged with catalyst **5** (0.010 mmol) and HBcat (0.012 mmol) in C_6D_6 (400 µL) at room temperature. After 15 min at room temperature, complete consumption of **1** is observed in the $^{31}P\{^1H\}$ and ^1H NMR spectrums to lead the complex **8.**

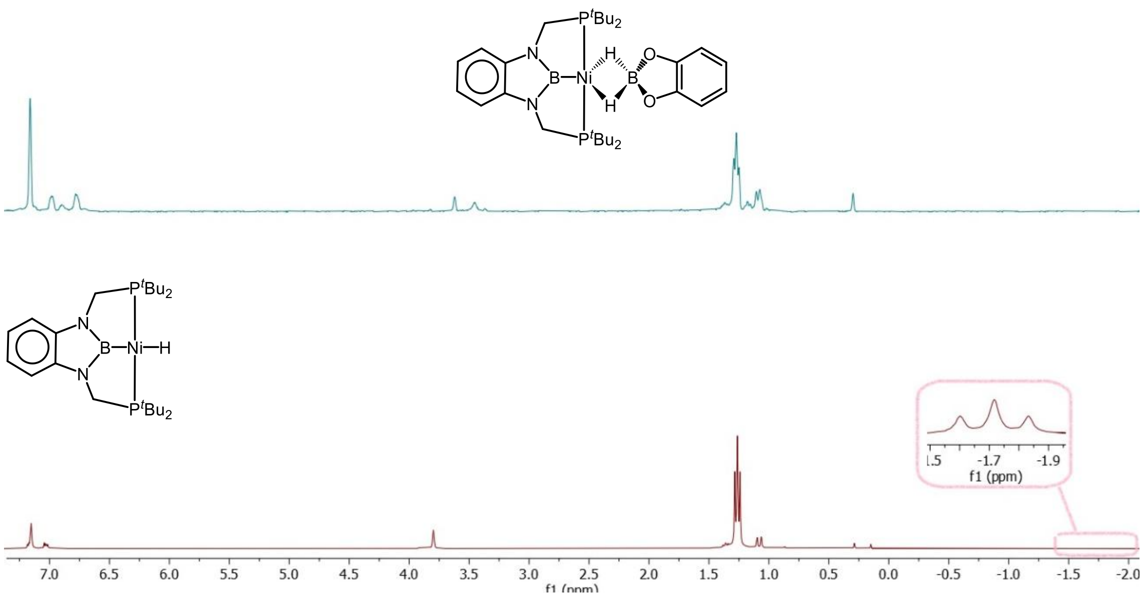

Figure S34. ^{1}H NMR spectrum (C_6D_6 at 298 K) of complex **5** (bottom) and ^{1}H NMR spectrum after addition of HBcat to form **8** (top).

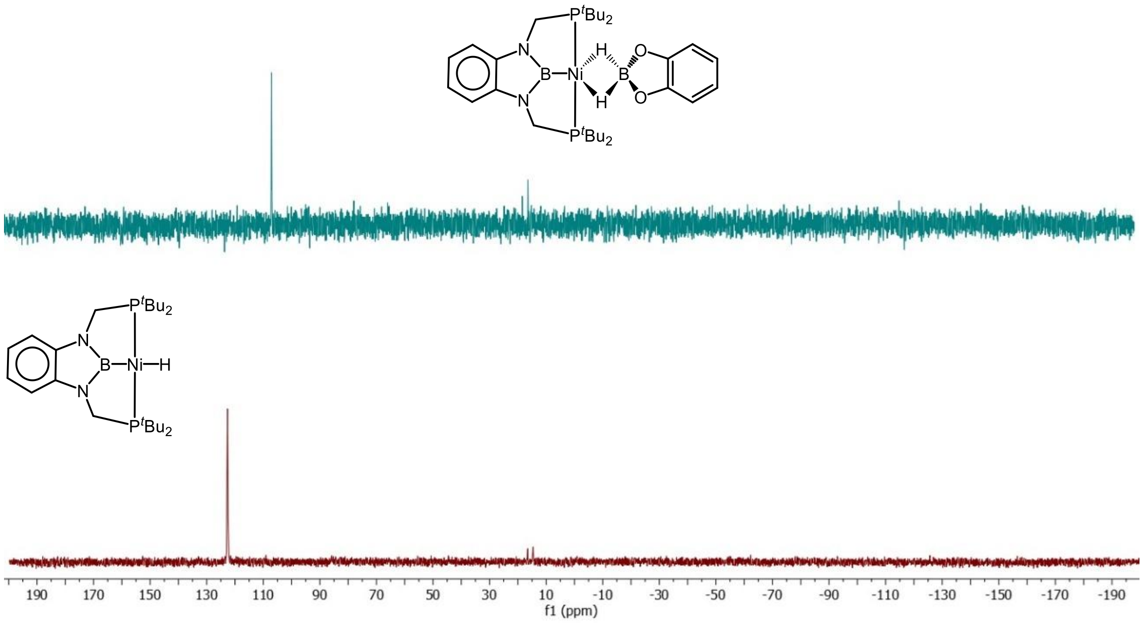

Figure S35. $^{31}P\{^{1}H\}$ NMR spectrum (C_6D_6 at 298 K) of complex **5** (bottom) and $^{31}P\{^{1}H\}$ NMR spectrum after addition of HBcat to form **8** (top).

7. Computational details

Computational Details

Gibbs energy profiles

Unless otherwise stated, calculations were performed using the M06-L functional,[3] as implemented in Gaussian 09[4] along with Grimme's D3 dispersion correction.[5] Geometry optimizations were performed in solution (solvent = benzene, ε = 2.27) using the continuum SMD model[6] and basis set 1 (BS1). BS1 uses the double-ζ 6-31G(d,p)[7] basis set for the H, C, N, O, B, P and Si atoms and the scalar relativistic Stuttgart−Dresden SDD pseudopotential[8] and its associated double-ζ basis set, complemented with a set of polarization functions, for the Ni atom.[9] The nature of the stationary points was confirmed by frequency analysis. Connections between the transition states and the minima were checked by perturbing the transition state geometry along the TS coordinate and optimizing until the corresponding minima. All energies in solution were corrected by single-point calculations with the larger basis set 2 (BS2) including triple-ζ def2TZVP basis set for the H, C, N, O, B, P and Si atoms, and def2QZVP for the Ni atom.[10] The scalar relativistic Stuttgart−Dresden SDD pseudopotential and its associated basis set for Ni was used in the energy profile calculations. Gibbs energies in benzene were calculated at 298.15 K. Gibbs energy corrections were obtained based on vibrational frequencies of the BS1 optimized structures using the quasi-harmonic approximation. Thermal contributions to the Gibbs energies were corrected by employing the approximation described by Grimme, where entropic terms for frequencies below a cut-off of 100 cm-1 were calculated using the free-rotor approximation.[11] The GoodVibes program developed by Paton and Funes-Ardoiz was employed to introduce these corrections.[12] All reported energies in the main text correspond to M06-LD3/BS2 Gibbs energies in benzene solvent (1 M) at 298.15 K in kcal mol^{-1}. Structure visualization was performed with Chemcraft software.[13, 14]

Calculated energy profiles using $PhSiH_3$

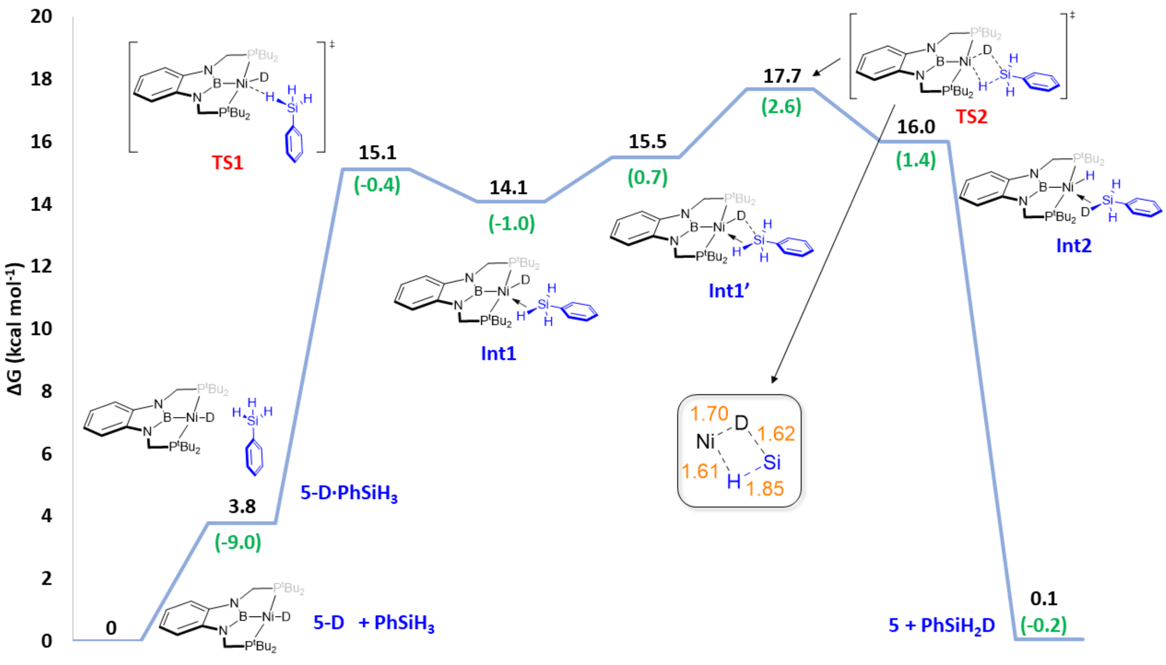

Figure S36. Gibbs energy profile in benzene for the for the H/D exchange in $PhSiH_3$ mediated by complex **5-D** (nucleophilic attack on Si). Gibbs energies at 298 K in kcal mol^{-1}. Enthalpy values highlighted in green.

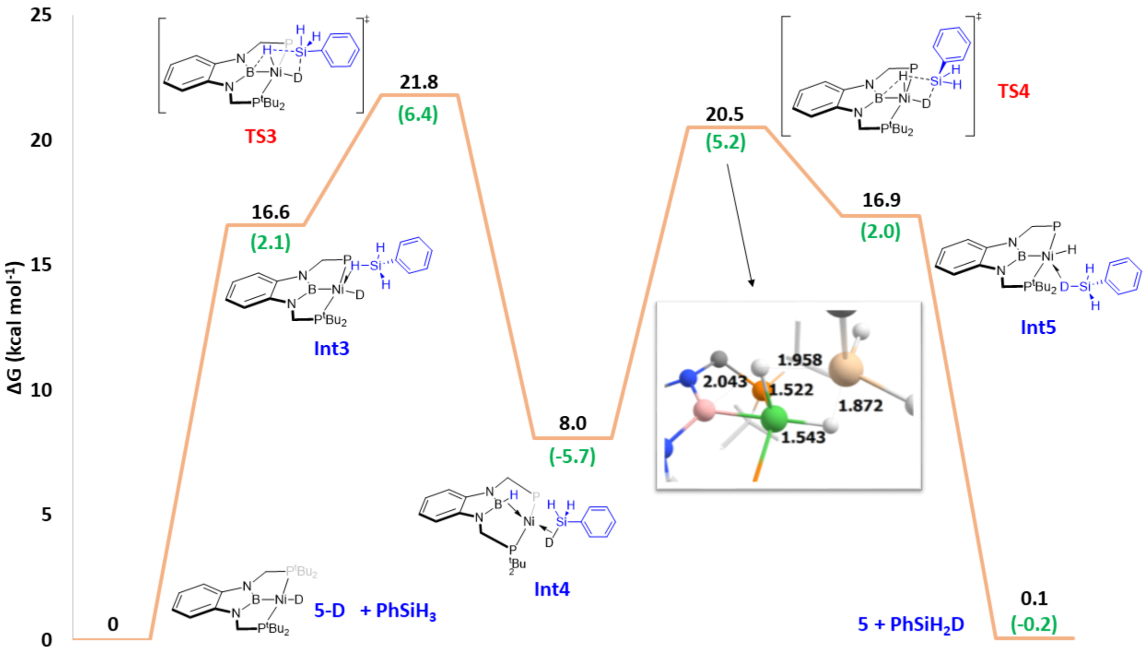

Figure S37. Gibbs energy profile in benzene for the for the H/D exchange in $PhSiH_3$ mediated by complex **5-D** (metal-ligand cooperativity). Gibbs energies at 298 K in kcal mol^{-1}. Enthalpy values highlighted in green.

Alternative mechanisms with high kinetic barriers (Exploratory calculations carried out with PBE0-D3BJ and BS1) [4][13]

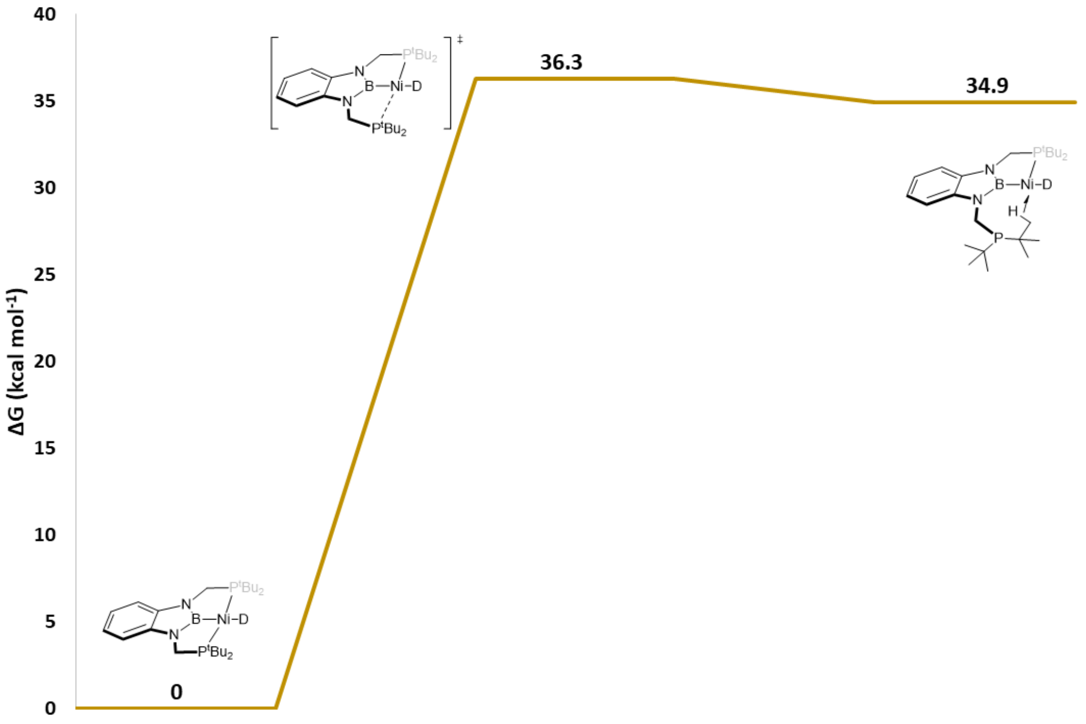

Figure S38. Gibbs energy profile in benzene for the phosphine decoordination process in **5-D**. Gibbs energies at 298 K in kcal mol^{-1}.

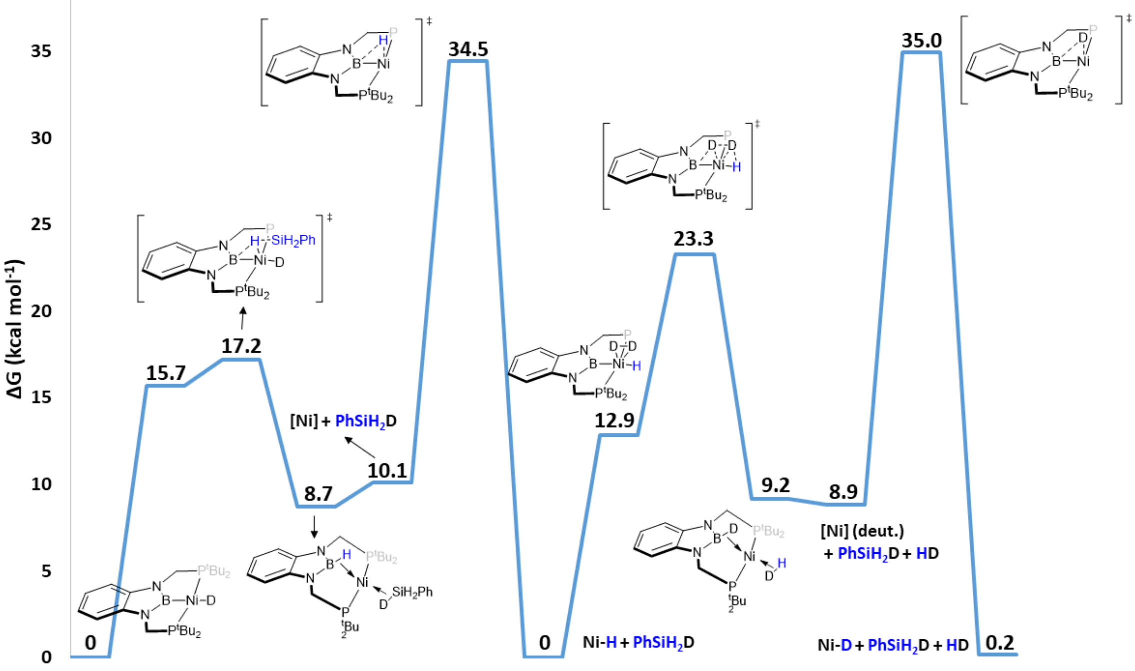

Figure S39. Gibbs energy profile in benzene for an alternative H/D exchange in PhSiH$_3$ mediated by complex **5-D** followed by D$_2$ activation. Gibbs energies at 298 K in kcal mol^{-1}. *NB*: in this mechanism, the oxidative addition steps take place with no hydrosilane occupying the

coordination vacant, which leads to a considerable increase in free energy. If D_2 is bound to Ni during these steps, a 9 kcal mol^{-1} decrease in energy from 35 kcal mol^{-1} to 26.1 kcal mol^{-1} is observed. On the other hand, hydrosilane coordination gives rise to the mechanism depicted in Figure S39.

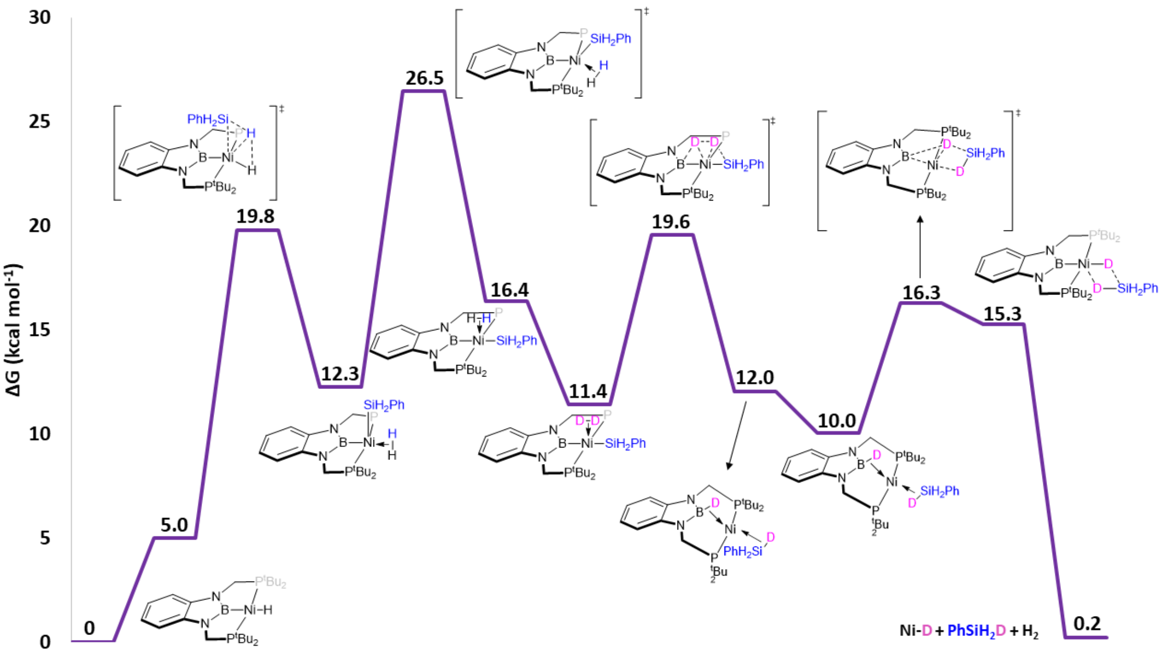

Figure S40. Gibbs energy profile in benzene for the H/D exchange in $PhSiH_3$ mediated by a nickel silyl complex. Gibbs energies at 298 K in kcal mol^{-1}.

8. Cartesian Coordinates

Mechanistic scenarios using $PhMeSiH_2$ (Figures 3 and 4 in the manuscript)

Ligand Cooperativity

D_2 (E = -1.16963004184; G = -1.175695)

H (Iso=2.014)	0.000000000	0.000000000	0.371885000
H (Iso=2.014)	0.000000000	0.000000000	-0.371885000

$PhMeSiH_2$ (E = -562.22687274; G = -562.118892)

Si	-2.342678000	-0.038679000	-0.043280000
H	-2.814225000	0.965815000	-1.031771000
C	-0.464013000	-0.036429000	-0.030785000
C	0.252566000	1.169934000	0.025546000
C	0.272683000	-1.229351000	-0.072262000
C	1.643568000	1.185285000	0.043985000
H	-0.285463000	2.116914000	0.053303000
C	1.665766000	-1.221163000	-0.054122000
H	-0.246886000	-2.185324000	-0.123062000
C	2.353775000	-0.012837000	0.004577000
H	2.175707000	2.132295000	0.087171000
H	2.214257000	-2.159192000	-0.088089000
H	3.440588000	-0.003404000	0.017382000
H	-2.855984000	0.399067000	1.281714000
C	-3.014240000	-1.736559000	-0.455483000
H	-2.729823000	-2.477122000	0.298243000
H	-4.106935000	-1.727976000	-0.502967000
H	-2.647085000	-2.090229000	-1.423584000

Complex 5-D (E = -1929.72946175; G = -1929.138049)

Ni	-0.000070000	-1.050336000	-0.000010000
B	0.000080000	0.867013000	0.000162000
P	-2.120560000	-0.644630000	-0.060382000

P	2.120490000	-0.644990000	0.060310000
N	-1.125188000	1.750359000	-0.185378000
N	1.125503000	1.750130000	0.185861000
C	-2.895763000	-0.847563000	1.656490000
C	-3.111544000	-1.518111000	-1.411432000
C	2.421367000	1.179517000	0.436534000
H	2.735343000	1.315096000	1.481071000
H	3.211392000	1.608249000	-0.197332000
C	-0.686973000	5.461912000	-0.119162000
H	-1.214939000	6.407073000	-0.212216000
C	-2.421129000	1.180004000	-0.436249000
H	-2.735017000	1.315854000	-1.480779000
H	-3.211124000	1.608725000	0.197659000
C	-0.704284000	3.067609000	-0.120798000
C	2.895526000	-0.847697000	-1.656667000
C	-1.398782000	4.265302000	-0.242916000
H	-2.469465000	4.266563000	-0.432878000
C	1.399552000	4.264999000	0.243846000
H	2.470235000	4.266016000	0.433814000
C	0.704827000	3.067462000	0.121501000
C	3.111420000	-1.518883000	1.411142000
C	0.687970000	5.461764000	0.120316000
H	1.216109000	6.406811000	0.213546000
C	2.496921000	-2.212251000	-2.221896000
H	1.408446000	-2.331687000	-2.242421000
H	2.875282000	-2.304858000	-3.248024000
H	2.903814000	-3.046088000	-1.643684000
C	4.414120000	-0.702094000	-1.664515000
H	4.908460000	-1.524965000	-1.138761000
H	4.777656000	-0.716624000	-2.699850000
H	4.749704000	0.241111000	-1.218069000

C	2.273651000	0.233205000	-2.548477000
H	1.179940000	0.159504000	-2.568485000
H	2.534120000	1.249647000	-2.235368000
H	2.639480000	0.104278000	-3.574632000
C	2.208001000	-1.433613000	2.647210000
H	1.249424000	-1.935818000	2.469373000
H	2.697924000	-1.918776000	3.500699000
H	1.995460000	-0.396577000	2.935057000
C	4.461210000	-0.875515000	1.728774000
H	4.929213000	-1.415681000	2.561603000
H	5.159508000	-0.913763000	0.889272000
H	4.367724000	0.168802000	2.042511000
C	3.311199000	-2.987020000	1.044061000
H	3.685877000	-3.535027000	1.917514000
H	2.372268000	-3.455823000	0.729513000
H	4.048314000	-3.112380000	0.243598000
C	-3.311562000	-2.986292000	-1.044649000
H	-3.686329000	-3.534057000	-1.918217000
H	-2.372713000	-3.455323000	-0.730194000
H	-4.048701000	-3.111698000	-0.244213000
C	-2.208063000	-1.432719000	-2.647448000
H	-1.995361000	-0.395655000	-2.935074000
H	-1.249566000	-1.935103000	-2.469681000
H	-2.698026000	-1.917633000	-3.501057000
C	-4.461228000	-0.874481000	-1.728978000
H	-4.929267000	-1.414383000	-2.561958000
H	-5.159571000	-0.912832000	-0.889516000
H	-4.367576000	0.169898000	-2.042456000
C	-2.497345000	-2.212262000	2.221500000
H	-1.408883000	-2.331798000	2.242138000
H	-2.875845000	-2.305042000	3.247561000

H	-2.904235000	-3.045949000	1.643070000
C	-2.273866000	0.233112000	2.548563000
H	-2.639769000	0.103994000	3.574667000
H	-1.180162000	0.159331000	2.568626000
H	-2.534243000	1.249640000	2.235655000
C	-4.414344000	-0.701824000	1.664215000
H	-4.908695000	-1.524555000	1.138254000
H	-4.777988000	-0.716517000	2.699510000
H	-4.749806000	0.241491000	1.217909000
H (Iso=2.014)	-0.000173000	-2.629759000	-0.000132000

Int3 (E = -2491.95995036; G = -2491.22766)

Ni	0.194169000	-0.256387000	0.214660000
B	-1.596784000	0.354066000	0.691701000
P	-0.996128000	-2.095721000	-0.084739000
P	0.364479000	1.954060000	-0.099334000
N	-2.840527000	-0.349394000	0.447408000
N	-2.009279000	1.714966000	0.964251000
C	-1.136121000	-3.302845000	1.416324000
C	-0.678877000	-3.073340000	-1.684352000
C	-1.040704000	2.768236000	0.835559000
H	-0.689775000	3.136448000	1.808570000
H	-1.449589000	3.629836000	0.288661000
C	-6.094310000	1.489964000	0.319542000
H	-7.156703000	1.373803000	0.123532000
C	-2.786079000	-1.585984000	-0.287565000
H	-3.010056000	-1.418352000	-1.351349000
H	-3.495996000	-2.337545000	0.070337000
C	-3.901339000	0.545936000	0.486779000
C	-0.022302000	2.390798000	-1.911526000
C	-5.255779000	0.373422000	0.232780000

H	-5.649513000	-0.603161000	-0.038907000
C	-4.223724000	2.931588000	0.900925000
H	-3.826691000	3.913113000	1.149688000
C	-3.384227000	1.827404000	0.820346000
C	1.824900000	3.009742000	0.521109000
C	-5.587053000	2.747058000	0.647615000
H	-6.258758000	3.599085000	0.705651000
C	0.966732000	1.662816000	-2.823964000
H	0.990023000	0.589918000	-2.607007000
H	0.660246000	1.797006000	-3.868945000
H	1.988484000	2.041941000	-2.730183000
C	-0.007457000	3.883416000	-2.233214000
H	0.995136000	4.317113000	-2.181794000
H	-0.365396000	4.029853000	-3.260141000
H	-0.669077000	4.464541000	-1.581259000
C	-1.427769000	1.850454000	-2.197061000
H	-1.499733000	0.779584000	-1.973629000
H	-2.203432000	2.371323000	-1.624355000
H	-1.656105000	1.990962000	-3.260997000
C	2.229528000	2.433368000	1.881001000
H	2.679052000	1.443508000	1.783300000
H	2.976215000	3.089140000	2.346392000
H	1.382069000	2.350345000	2.571611000
C	1.444492000	4.480356000	0.731640000
H	2.345267000	5.030428000	1.031740000
H	1.058718000	4.962914000	-0.168271000
H	0.712082000	4.617962000	1.532183000
C	3.013730000	2.939549000	-0.434297000
H	3.896431000	3.374049000	0.051726000
H	3.265698000	1.913530000	-0.713383000
H	2.835631000	3.512886000	-1.349693000

C	0.767365000	-3.563832000	-1.747863000
H	0.940348000	-4.033388000	-2.724114000
H	1.466691000	-2.727686000	-1.648911000
H	1.012057000	-4.303201000	-0.982603000
C	-0.864188000	-2.088259000	-2.843686000
H	-1.900705000	-1.763289000	-2.975776000
H	-0.238855000	-1.198622000	-2.706726000
H	-0.558609000	-2.574649000	-3.778010000
C	-1.644057000	-4.245210000	-1.857161000
H	-1.499795000	-4.693571000	-2.848485000
H	-1.478048000	-5.037768000	-1.121129000
H	-2.694111000	-3.936579000	-1.795080000
C	-0.030384000	-4.356915000	1.403762000
H	0.968127000	-3.924465000	1.306301000
H	-0.057507000	-4.906435000	2.353287000
H	-0.165864000	-5.092407000	0.605211000
C	-0.998152000	-2.459771000	2.686105000
H	-1.128477000	-3.108015000	3.562244000
H	-0.018689000	-1.980886000	2.761379000
H	-1.754081000	-1.669188000	2.738634000
C	-2.483750000	-4.029111000	1.493783000
H	-2.764424000	-4.540843000	0.568959000
H	-2.419228000	-4.793543000	2.277788000
H	-3.300173000	-3.358899000	1.777582000
H (Iso=2.014)	1.391294000	-0.680770000	-0.699913000
Si	2.368306000	-1.214724000	1.098752000
H	2.178176000	-2.581767000	0.559917000
H	1.125051000	-0.402459000	1.635507000
C	3.862127000	-0.445031000	0.219356000
C	4.017256000	-0.533162000	-1.173805000
C	4.895120000	0.171817000	0.941360000

C	5.137359000	-0.016445000	-1.817816000
H	3.230324000	-0.999950000	-1.765793000
C	6.011816000	0.710143000	0.304060000
H	4.823757000	0.244061000	2.025786000
C	6.134196000	0.619991000	-1.079575000
H	5.230171000	-0.102135000	-2.897930000
H	6.787812000	1.198332000	0.889026000
H	7.003690000	1.038423000	-1.580613000
C	3.013674000	-1.548208000	2.864398000
H	3.968708000	-2.086315000	2.866541000
H	3.148249000	-0.629651000	3.448547000
H	2.294614000	-2.163118000	3.419509000

TS3 (E = -2491.9539108; G = -2491.222165)

Ni	0.276906000	-0.276480000	0.417191000
B	-1.478005000	0.392609000	0.886204000
P	-0.992318000	-2.095506000	-0.140139000
P	0.392741000	1.930126000	-0.252082000
N	-2.726643000	-0.321795000	0.762759000
N	-1.858196000	1.776271000	1.080699000
C	-0.800798000	-3.626772000	0.995224000
C	-0.967731000	-2.687638000	-1.956243000
C	-0.908304000	2.812821000	0.766423000
H	-0.454827000	3.257461000	1.662774000
H	-1.379045000	3.629673000	0.200666000
C	-5.980214000	1.521407000	0.738493000
H	-7.054038000	1.395194000	0.631989000
C	-2.782437000	-1.653412000	0.221721000
H	-3.397173000	-1.683525000	-0.689287000
H	-3.228209000	-2.365205000	0.926363000
C	-3.781270000	0.581164000	0.827951000

C	-0.184383000	2.250077000	-2.039940000
C	-5.150039000	0.395401000	0.688825000
H	-5.562790000	-0.599029000	0.536720000
C	-4.071813000	2.993091000	1.064896000
H	-3.657447000	3.988571000	1.205645000
C	-3.241231000	1.881378000	1.019985000
C	1.915788000	3.015750000	0.111284000
C	-5.450283000	2.797749000	0.921655000
H	-6.115201000	3.656274000	0.955597000
C	0.620694000	1.313887000	-2.944388000
H	0.664408000	0.298293000	-2.535834000
H	0.168444000	1.267159000	-3.943145000
H	1.652578000	1.657695000	-3.066446000
C	-0.040204000	3.680117000	-2.550868000
H	1.005017000	3.977206000	-2.675767000
H	-0.514183000	3.755096000	-3.538125000
H	-0.536490000	4.411398000	-1.903314000
C	-1.675251000	1.894385000	-2.090168000
H	-1.904920000	0.936148000	-1.615812000
H	-2.289990000	2.658053000	-1.600529000
H	-2.001492000	1.836811000	-3.136082000
C	2.359280000	2.670065000	1.532612000
H	2.728417000	1.649146000	1.597274000
H	3.185954000	3.329360000	1.826620000
H	1.558753000	2.792704000	2.270538000
C	1.632528000	4.521133000	0.084710000
H	2.531119000	5.047621000	0.430732000
H	1.399665000	4.910360000	-0.905583000
H	0.821730000	4.807578000	0.762712000
C	3.043993000	2.669307000	-0.857963000
H	3.984282000	3.118265000	-0.513167000

H	3.207575000	1.587152000	-0.923751000
H	2.851757000	3.050509000	-1.866294000
C	0.495137000	-2.807061000	-2.391534000
H	0.536695000	-3.060586000	-3.458068000
H	1.031385000	-1.862912000	-2.246714000
H	1.038444000	-3.585257000	-1.847607000
C	-1.621504000	-1.601428000	-2.808840000
H	-2.693188000	-1.488795000	-2.614282000
H	-1.145456000	-0.632558000	-2.659539000
H	-1.511452000	-1.863160000	-3.868499000
C	-1.689974000	-4.003549000	-2.229607000
H	-1.719037000	-4.174892000	-3.313355000
H	-1.177490000	-4.860637000	-1.786061000
H	-2.727330000	-3.995864000	-1.877223000
C	0.493138000	-4.368128000	0.653702000
H	1.340186000	-3.693975000	0.502938000
H	0.757504000	-5.046685000	1.474050000
H	0.378551000	-4.981250000	-0.246902000
C	-0.752413000	-3.092543000	2.431570000
H	-0.723257000	-3.940451000	3.127045000
H	0.122684000	-2.472256000	2.624166000
H	-1.641596000	-2.499306000	2.676898000
C	-1.953656000	-4.638503000	0.952998000
H	-2.119749000	-5.090837000	-0.023734000
H	-1.704886000	-5.455704000	1.642175000
H	-2.902949000	-4.223931000	1.303212000
H (Iso=2.014)	1.580526000	-0.848626000	-0.138937000
Si	2.139292000	-0.868801000	1.664974000
H	2.115131000	-2.350837000	1.798744000
H	0.250652000	-0.190332000	1.937796000
C	3.854465000	-0.531274000	0.882804000

C	4.149769000	-1.016598000	-0.401488000
C	4.878424000	0.153455000	1.553593000
C	5.397954000	-0.832486000	-0.988926000
H	3.376756000	-1.547469000	-0.960195000
C	6.128170000	0.359300000	0.969164000
H	4.693569000	0.549645000	2.551861000
C	6.391734000	-0.134041000	-0.305129000
H	5.594392000	-1.226142000	-1.983646000
H	6.896900000	0.905509000	1.511024000
H	7.364469000	0.024229000	-0.763937000
C	2.325143000	-0.308439000	3.475452000
H	3.246908000	-0.702103000	3.920527000
H	2.323810000	0.777205000	3.613329000
H	1.485548000	-0.701625000	4.059810000

Int4 (E = -2491.97258686; G = -2491.239812)

Ni	0.326536000	-0.291409000	0.460478000
B	-1.456560000	0.407566000	1.176067000
P	-1.023617000	-2.152934000	-0.027339000
P	0.412241000	1.925441000	-0.225021000
N	-2.700601000	-0.320076000	0.969271000
N	-1.849862000	1.816377000	1.158270000
C	-0.715881000	-3.746739000	0.989122000
C	-1.284382000	-2.658059000	-1.847540000
C	-0.876994000	2.821035000	0.804115000
H	-0.397486000	3.263252000	1.687537000
H	-1.343453000	3.643890000	0.245552000
C	-5.878873000	1.503646000	0.233989000
H	-6.927197000	1.361024000	-0.013819000
C	-2.738190000	-1.711111000	0.604438000
H	-3.506559000	-1.897089000	-0.158757000

H	-2.989033000	-2.345282000	1.462429000
C	-3.731163000	0.585045000	0.768725000
C	-0.163394000	2.250805000	-2.012835000
C	-5.067447000	0.382183000	0.447456000
H	-5.471235000	-0.624030000	0.362699000
C	-4.016960000	3.005676000	0.663915000
H	-3.612104000	4.012095000	0.743896000
C	-3.205694000	1.899912000	0.880545000
C	1.947192000	2.994847000	0.132112000
C	-5.362771000	2.793308000	0.339802000
H	-6.011196000	3.648935000	0.172213000
C	0.580111000	1.254339000	-2.904436000
H	0.523789000	0.237321000	-2.499947000
H	0.149178000	1.249807000	-3.913922000
H	1.641894000	1.505188000	-2.999750000
C	0.044127000	3.661923000	-2.552275000
H	1.099852000	3.913046000	-2.688814000
H	-0.433923000	3.741052000	-3.537561000
H	-0.416368000	4.424023000	-1.913780000
C	-1.670520000	1.970943000	-2.035885000
H	-1.951075000	1.072721000	-1.478756000
H	-2.240752000	2.805164000	-1.612923000
H	-2.007713000	1.840911000	-3.071975000
C	2.381526000	2.640738000	1.554874000
H	2.630442000	1.584149000	1.641465000
H	3.278677000	3.214528000	1.819879000
H	1.610963000	2.868047000	2.300450000
C	1.698270000	4.505290000	0.095788000
H	2.593606000	5.015798000	0.473595000
H	1.511316000	4.894917000	-0.904667000
H	0.867554000	4.810826000	0.740770000

C	3.069888000	2.620559000	-0.832627000
H	4.010024000	3.089349000	-0.514562000
H	3.241760000	1.538582000	-0.857894000
H	2.867827000	2.958509000	-1.854147000
C	0.094144000	-2.812531000	-2.496616000
H	-0.019851000	-2.920791000	-3.582530000
H	0.722642000	-1.932766000	-2.312481000
H	0.637233000	-3.690226000	-2.136726000
C	-1.987947000	-1.486697000	-2.531699000
H	-2.973369000	-1.261473000	-2.107936000
H	-1.383136000	-0.581611000	-2.475087000
H	-2.135357000	-1.721309000	-3.593251000
C	-2.109244000	-3.920425000	-2.077130000
H	-2.314835000	-4.026703000	-3.150216000
H	-1.583746000	-4.826457000	-1.763474000
H	-3.078545000	-3.888346000	-1.566309000
C	0.496745000	-4.492142000	0.432364000
H	1.341862000	-3.829512000	0.223217000
H	0.840300000	-5.238090000	1.160210000
H	0.248738000	-5.031453000	-0.487804000
C	-0.427157000	-3.273559000	2.417158000
H	-0.268997000	-4.143806000	3.066281000
H	0.466119000	-2.650590000	2.471240000
H	-1.259515000	-2.697818000	2.840440000
C	-1.890708000	-4.728121000	1.074317000
H	-2.166678000	-5.170651000	0.117653000
H	-1.597353000	-5.555089000	1.734038000
H	-2.789704000	-4.287582000	1.514992000
H (Iso=2.014)	1.672341000	-0.812130000	-0.074922000
Si	2.348553000	-1.052474000	1.455854000
H	2.455297000	-2.540054000	1.458403000

H	-0.487995000	-0.009952000	1.909813000
C	4.066517000	-0.499228000	0.873154000
C	4.492217000	-0.817726000	-0.427597000
C	4.956649000	0.221930000	1.682101000
C	5.742853000	-0.433237000	-0.900794000
H	3.820843000	-1.368879000	-1.089547000
C	6.208057000	0.619348000	1.215220000
H	4.662170000	0.493556000	2.695679000
C	6.603841000	0.293247000	-0.079164000
H	6.045546000	-0.692792000	-1.912532000
H	6.874134000	1.185861000	1.861863000
H	7.578030000	0.604192000	-0.448001000
C	2.176600000	-0.577291000	3.286768000
H	3.033153000	-0.961482000	3.855697000
H	2.115550000	0.501638000	3.454031000
H	1.270403000	-1.018283000	3.712356000

TS4 (E = -2491.95401451; G = -2491.222765)

Ni	0.242800000	0.488638000	-0.527367000
B	-0.635434000	-0.481599000	0.896971000
P	-1.804800000	1.544908000	-0.353101000
P	0.860082000	-1.624834000	-1.045516000
N	-1.644427000	0.050446000	1.786312000
N	-0.714029000	-1.916871000	1.038317000
C	-3.315541000	0.706103000	-1.177083000
C	-1.885363000	3.426214000	-0.664777000
C	-0.242907000	-2.754888000	-0.036159000
H	-1.089425000	-3.101311000	-0.650798000
H	0.287930000	-3.650638000	0.313951000
C	-3.924320000	-2.261048000	3.642346000
H	-4.774464000	-2.290319000	4.318245000

C	-2.176614000	1.361946000	1.479457000
H	-1.667353000	2.148081000	2.048446000
H	-3.246341000	1.423416000	1.712228000
C	-2.322248000	-1.007234000	2.380225000
C	2.566055000	-2.162035000	-0.359664000
C	-3.410520000	-1.022371000	3.243208000
H	-3.852163000	-0.092632000	3.594094000
C	-2.273153000	-3.448096000	2.309978000
H	-1.847352000	-4.377229000	1.939081000
C	-1.751081000	-2.224116000	1.915365000
C	0.677912000	-2.284611000	-2.824962000
C	-3.366581000	-3.453581000	3.183134000
H	-3.786097000	-4.402677000	3.504923000
C	3.683294000	-1.273348000	-0.896243000
H	3.496633000	-0.225150000	-0.667699000
H	4.624426000	-1.541062000	-0.399286000
H	3.840899000	-1.369848000	-1.972329000
C	2.922724000	-3.619346000	-0.649061000
H	3.163998000	-3.787434000	-1.703413000
H	3.817673000	-3.885526000	-0.072555000
H	2.136818000	-4.325301000	-0.360801000
C	2.503834000	-1.953540000	1.158249000
H	2.226748000	-0.925742000	1.415261000
H	1.799686000	-2.629562000	1.652508000
H	3.498073000	-2.140077000	1.583378000
C	-0.569798000	-1.621576000	-3.410281000
H	-0.510628000	-0.530674000	-3.336148000
H	-0.664467000	-1.893473000	-4.469096000
H	-1.486925000	-1.946892000	-2.909025000
C	0.505801000	-3.800623000	-2.932279000
H	0.331947000	-4.063996000	-3.983528000

H	1.381761000	-4.361899000	-2.603682000
H	-0.360823000	-4.164460000	-2.371068000
C	1.879072000	-1.839909000	-3.658104000
H	1.672341000	-2.023960000	-4.719497000
H	2.082804000	-0.768093000	-3.546037000
H	2.787051000	-2.394873000	-3.406260000
C	-1.139410000	3.733012000	-1.965423000
H	-1.146590000	4.815669000	-2.142786000
H	-0.097246000	3.405181000	-1.925381000
H	-1.604210000	3.254681000	-2.833804000
C	-1.177415000	4.120422000	0.506990000
H	-1.825620000	4.181685000	1.387014000
H	-0.246828000	3.629487000	0.801429000
H	-0.925528000	5.147825000	0.217905000
C	-3.293109000	4.013535000	-0.748874000
H	-3.213570000	5.107114000	-0.799403000
H	-3.837509000	3.692707000	-1.641188000
H	-3.898523000	3.775740000	0.131785000
C	-3.224632000	0.987874000	-2.676840000
H	-2.226769000	0.762525000	-3.069362000
H	-3.944485000	0.360767000	-3.217680000
H	-3.453855000	2.030021000	-2.919534000
C	-3.171372000	-0.801118000	-0.948353000
H	-3.888422000	-1.333316000	-1.586801000
H	-2.166780000	-1.152945000	-1.194405000
H	-3.386384000	-1.084411000	0.086779000
C	-4.695965000	1.102376000	-0.651187000
H	-4.976562000	2.130900000	-0.876873000
H	-5.446330000	0.455066000	-1.122606000
H	-4.793178000	0.946835000	0.428842000
Si	2.023277000	1.962205000	-0.398684000

H	0.770498000	1.016876000	0.794396000
H (Iso=2.014)	0.848666000	1.293195000	-1.693186000
H	1.471192000	3.333615000	-0.224402000
C	3.187565000	1.730956000	1.106097000
C	2.668191000	1.750639000	2.410516000
C	4.571238000	1.543808000	0.974983000
C	3.481476000	1.595139000	3.527850000
H	1.592521000	1.872697000	2.555005000
C	5.394061000	1.362124000	2.086197000
H	5.018090000	1.520034000	-0.018802000
C	4.850942000	1.388674000	3.367269000
H	3.047074000	1.620831000	4.524617000
H	6.461264000	1.201439000	1.950093000
H	5.488835000	1.246945000	4.236175000
C	3.203369000	2.182583000	-1.875595000
H	4.003120000	2.894757000	-1.642058000
H	3.673306000	1.257884000	-2.224526000
H	2.640890000	2.588848000	-2.724167000

Int5 (E = -2491.95584099; G = -2491.225184)

Ni	0.258072000	0.264994000	-0.535549000
B	-0.982403000	-0.347044000	0.839645000
P	-1.316363000	1.855595000	-0.460648000
P	0.415200000	-1.958662000	-0.746068000
N	-1.853922000	0.480015000	1.646483000
N	-1.475511000	-1.689652000	1.021910000
C	-2.915285000	1.434493000	-1.435432000
C	-0.833205000	3.667901000	-0.799512000
C	-1.092071000	-2.703510000	0.077317000
H	-1.887201000	-2.868480000	-0.668292000
H	-0.875086000	-3.674700000	0.543971000

C	-4.806180000	-1.014972000	3.391334000
H	-5.672850000	-0.776278000	4.001358000
C	-1.921885000	1.885196000	1.311749000
H	-1.259779000	2.490835000	1.941854000
H	-2.938199000	2.281329000	1.415379000
C	-2.841992000	-0.312716000	2.216107000
C	1.785512000	-2.801664000	0.292115000
C	-3.944058000	0.013438000	2.997791000
H	-4.131587000	1.044238000	3.289304000
C	-3.468074000	-2.675928000	2.223968000
H	-3.293276000	-3.703868000	1.915591000
C	-2.606123000	-1.662155000	1.827477000
C	0.360891000	-2.730310000	-2.485601000
C	-4.571887000	-2.337007000	3.012914000
H	-5.258252000	-3.116636000	3.331487000
C	3.157793000	-2.218026000	-0.027311000
H	3.208155000	-1.154137000	0.213961000
H	3.912367000	-2.715395000	0.594787000
H	3.452456000	-2.355968000	-1.070439000
C	1.847999000	-4.316677000	0.107759000
H	2.258462000	-4.595009000	-0.867515000
H	2.518358000	-4.738695000	0.866913000
H	0.877894000	-4.810768000	0.226698000
C	1.487067000	-2.490988000	1.762900000
H	1.406163000	-1.413414000	1.941770000
H	0.568472000	-2.964082000	2.122849000
H	2.314803000	-2.868153000	2.375907000
C	-0.652068000	-1.910250000	-3.289164000
H	-0.430046000	-0.838226000	-3.249077000
H	-0.628357000	-2.233168000	-4.337377000
H	-1.675787000	-2.061943000	-2.929610000

C	-0.092209000	-4.192466000	-2.520872000
H	-0.258690000	-4.484820000	-3.565466000
H	0.642291000	-4.886377000	-2.110677000
H	-1.039256000	-4.350631000	-1.994220000
C	1.730330000	-2.590405000	-3.145279000
H	1.658012000	-2.861419000	-4.205672000
H	2.101868000	-1.560320000	-3.093165000
H	2.477286000	-3.250935000	-2.693712000
C	-0.081216000	3.774432000	-2.128684000
H	0.385679000	4.764275000	-2.202209000
H	0.706669000	3.024566000	-2.224508000
H	-0.749001000	3.667386000	-2.987096000
C	0.085070000	4.086550000	0.361145000
H	-0.492790000	4.363238000	1.248610000
H	0.796824000	3.310059000	0.652501000
H	0.667528000	4.967113000	0.066403000
C	-2.012630000	4.639196000	-0.823218000
H	-1.625324000	5.665680000	-0.859341000
H	-2.646732000	4.506460000	-1.704422000
H	-2.638755000	4.562932000	0.072724000
C	-2.660519000	1.757646000	-2.908453000
H	-1.707834000	1.343293000	-3.259694000
H	-3.457841000	1.319319000	-3.521145000
H	-2.658593000	2.834008000	-3.104117000
C	-3.172849000	-0.068468000	-1.312166000
H	-3.978550000	-0.349039000	-2.003107000
H	-2.283922000	-0.649911000	-1.567512000
H	-3.494902000	-0.351617000	-0.305520000
C	-4.181294000	2.144397000	-0.952957000
H	-4.120268000	3.232843000	-0.978528000
H	-5.014384000	1.853550000	-1.605240000

H -4.463074000 1.839230000 0.060382000

Si 2.477430000 1.260691000 -0.599006000

H 1.363164000 0.769589000 0.510594000

H (Iso=2.014) 0.928848000 0.731601000 -1.857376000

H 2.245876000 2.704138000 -0.854124000

C 3.719429000 1.271607000 0.873312000

C 3.240779000 1.470445000 2.178940000

C 5.104370000 1.091160000 0.729494000

C 4.090144000 1.508937000 3.280875000

H 2.165924000 1.582971000 2.339145000

C 5.964589000 1.114267000 1.826341000

H 5.527154000 0.915640000 -0.258530000

C 5.460452000 1.327182000 3.106524000

H 3.683026000 1.668259000 4.276777000

H 7.031967000 0.962520000 1.680954000

H 6.129189000 1.343351000 3.963500000

C 3.524817000 0.657359000 -2.058713000

H 4.432516000 1.267385000 -2.138095000

H 3.840948000 -0.386482000 -1.982174000

H 2.969715000 0.771847000 -2.994432000

Complex 5 (E = -1929.72946175; G = -1929.13599)

Ni -0.000070000 -1.050336000 -0.000010000

B 0.000080000 0.867013000 0.000162000

P -2.120560000 -0.644630000 -0.060382000

P 2.120490000 -0.644990000 0.060310000

N -1.125188000 1.750359000 -0.185378000

N 1.125503000 1.750130000 0.185861000

C -2.895763000 -0.847563000 1.656490000

C -3.111544000 -1.518111000 -1.411432000

C 2.421367000 1.179517000 0.436534000

H	2.735343000	1.315096000	1.481071000
H	3.211392000	1.608249000	-0.197332000
C	-0.686973000	5.461912000	-0.119162000
H	-1.214939000	6.407073000	-0.212216000
C	-2.421129000	1.180004000	-0.436249000
H	-2.735017000	1.315854000	-1.480779000
H	-3.211124000	1.608725000	0.197659000
C	-0.704284000	3.067609000	-0.120798000
C	2.895526000	-0.847697000	-1.656667000
C	-1.398782000	4.265302000	-0.242916000
H	-2.469465000	4.266563000	-0.432878000
C	1.399552000	4.264999000	0.243846000
H	2.470235000	4.266016000	0.433814000
C	0.704827000	3.067462000	0.121501000
C	3.111420000	-1.518883000	1.411142000
C	0.687970000	5.461764000	0.120316000
H	1.216109000	6.406811000	0.213546000
C	2.496921000	-2.212251000	-2.221896000
H	1.408446000	-2.331687000	-2.242421000
H	2.875282000	-2.304858000	-3.248024000
H	2.903814000	-3.046088000	-1.643684000
C	4.414120000	-0.702094000	-1.664515000
H	4.908460000	-1.524965000	-1.138761000
H	4.777656000	-0.716624000	-2.699850000
H	4.749704000	0.241111000	-1.218069000
C	2.273651000	0.233205000	-2.548477000
H	1.179940000	0.159504000	-2.568485000
H	2.534120000	1.249647000	-2.235368000
H	2.639480000	0.104278000	-3.574632000
C	2.208001000	-1.433613000	2.647210000
H	1.249424000	-1.935818000	2.469373000

H	2.697924000	-1.918776000	3.500699000
H	1.995460000	-0.396577000	2.935057000
C	4.461210000	-0.875515000	1.728774000
H	4.929213000	-1.415681000	2.561603000
H	5.159508000	-0.913763000	0.889272000
H	4.367724000	0.168802000	2.042511000
C	3.311199000	-2.987020000	1.044061000
H	3.685877000	-3.535027000	1.917514000
H	2.372268000	-3.455823000	0.729513000
H	4.048314000	-3.112380000	0.243598000
C	-3.311562000	-2.986292000	-1.044649000
H	-3.686329000	-3.534057000	-1.918217000
H	-2.372713000	-3.455323000	-0.730194000
H	-4.048701000	-3.111698000	-0.244213000
C	-2.208063000	-1.432719000	-2.647448000
H	-1.995361000	-0.395655000	-2.935074000
H	-1.249566000	-1.935103000	-2.469681000
H	-2.698026000	-1.917633000	-3.501057000
C	-4.461228000	-0.874481000	-1.728978000
H	-4.929267000	-1.414383000	-2.561958000
H	-5.159571000	-0.912832000	-0.889516000
H	-4.367576000	0.169898000	-2.042456000
C	-2.497345000	-2.212262000	2.221500000
H	-1.408883000	-2.331798000	2.242138000
H	-2.875845000	-2.305042000	3.247561000
H	-2.904235000	-3.045949000	1.643070000
C	-2.273866000	0.233112000	2.548563000
H	-2.639769000	0.103994000	3.574667000
H	-1.180162000	0.159331000	2.568626000
H	-2.534243000	1.249640000	2.235655000
C	-4.414344000	-0.701824000	1.664215000

H	-4.908695000	-1.524555000	1.138254000
H	-4.777988000	-0.716517000	2.699510000
H	-4.749806000	0.241491000	1.217909000
H	-0.000173000	-2.629759000	-0.000132000

PhMeSiHD (E = -562.226903096; G = -562.118892)

H (Iso=2.014)	1.326375000	-0.329472000	0.703843000
Si	2.352779000	-1.009283000	1.536096000
H	2.071916000	-2.465985000	1.444807000
C	4.057763000	-0.654521000	0.830727000
C	4.368602000	-0.964459000	-0.503545000
C	5.064106000	-0.070819000	1.614248000
C	5.628645000	-0.704027000	-1.032232000
H	3.612583000	-1.417768000	-1.143769000
C	6.328160000	0.192808000	1.091077000
H	4.861072000	0.184549000	2.653227000
C	6.612536000	-0.123531000	-0.234128000
H	5.844690000	-0.953190000	-2.068001000
H	7.091152000	0.645983000	1.719033000
H	7.597790000	0.080442000	-0.645272000
C	2.230343000	-0.436866000	3.314053000
H	2.407068000	0.639239000	3.404081000
H	1.237232000	-0.638073000	3.725966000
H	2.956288000	-0.947583000	3.954096000

Nucleophilic attack on Si

Complex 5-D·PhMeSiH$_2$ (E = -2491.97191397; G = -2491.24694)

Ni	-0.154703000	0.164555000	-0.066203000
B	-2.015326000	-0.294469000	0.027370000
P	-1.042601000	2.110066000	0.192149000
P	-0.055457000	-1.976376000	-0.306656000

N	-3.123533000	0.566982000	0.358180000
N	-2.619677000	-1.576795000	-0.239231000
C	-1.146469000	3.015194000	-1.466300000
C	-0.328698000	3.195749000	1.560478000
C	-1.777390000	-2.669463000	-0.647167000
H	-1.897379000	-2.903328000	-1.714624000
H	-1.971548000	-3.596417000	-0.087612000
C	-6.627664000	-0.738789000	0.384009000
H	-7.662740000	-0.462775000	0.566005000
C	-2.854472000	1.941390000	0.689542000
H	-2.983063000	2.137654000	1.763694000
H	-3.500964000	2.649564000	0.150399000
C	-4.307932000	-0.147807000	0.298707000
C	0.422239000	-2.811929000	1.319624000
C	-5.626004000	0.224979000	0.535308000
H	-5.867898000	1.242867000	0.831541000
C	-4.998698000	-2.436018000	-0.233967000
H	-4.758711000	-3.454191000	-0.530771000
C	-3.992417000	-1.489060000	-0.084494000
C	0.958087000	-2.606353000	-1.769371000
C	-6.319522000	-2.046339000	0.005769000
H	-7.117446000	-2.775207000	-0.106481000
C	1.671498000	-2.118701000	1.868648000
H	1.499512000	-1.043275000	1.986987000
H	1.924375000	-2.541002000	2.850259000
H	2.547789000	-2.233224000	1.224273000
C	0.661884000	-4.312725000	1.190882000
H	1.549757000	-4.536432000	0.591530000
H	0.827270000	-4.744273000	2.186272000
H	-0.191799000	-4.839330000	0.748372000
C	-0.723989000	-2.560972000	2.305894000

H	-0.919118000	-1.489460000	2.431919000
H	-1.662236000	-3.040483000	2.008028000
H	-0.445883000	-2.969406000	3.285521000
C	0.602486000	-1.640658000	-2.906232000
H	0.856666000	-0.608363000	-2.634608000
H	1.164296000	-1.906736000	-3.810606000
H	-0.464757000	-1.671122000	-3.159456000
C	0.644140000	-4.037440000	-2.202788000
H	1.240913000	-4.283706000	-3.090320000
H	0.888932000	-4.776655000	-1.436070000
H	-0.406111000	-4.173394000	-2.480442000
C	2.444483000	-2.464280000	-1.453252000
H	3.031579000	-2.581241000	-2.373783000
H	2.666420000	-1.476258000	-1.035611000
H	2.792625000	-3.224869000	-0.745174000
C	1.014879000	3.767716000	1.115117000
H	1.541239000	4.191247000	1.979956000
H	1.653433000	2.993332000	0.674866000
H	0.897171000	4.572678000	0.381319000
C	-0.092391000	2.225068000	2.724427000
H	-1.021607000	1.751123000	3.064695000
H	0.605292000	1.427384000	2.439987000
H	0.332285000	2.766183000	3.579496000
C	-1.242525000	4.327581000	2.032003000
H	-0.755977000	4.858306000	2.860176000
H	-1.443888000	5.066661000	1.252752000
H	-2.203435000	3.965609000	2.410937000
C	0.233589000	3.000507000	-2.128418000
H	0.620010000	1.979194000	-2.223570000
H	0.158176000	3.439105000	-3.131760000
H	0.976319000	3.577615000	-1.570848000

C	-2.101761000	2.203865000	-2.349459000
H	-2.125431000	2.647353000	-3.352554000
H	-1.771641000	1.163218000	-2.447893000
H	-3.130421000	2.193987000	-1.973522000
C	-1.656855000	4.448376000	-1.352886000
H	-0.947943000	5.099066000	-0.831175000
H	-1.799944000	4.867443000	-2.356966000
H	-2.622252000	4.510257000	-0.837333000
H (Iso=2.014)	1.369657000	0.545798000	-0.160658000
Si	3.924486000	1.350438000	-1.396996000
H	3.183363000	2.551447000	-0.940895000
C	4.438047000	0.356102000	0.111848000
C	4.020407000	0.721343000	1.400041000
C	5.269464000	-0.768404000	-0.008234000
C	4.416126000	-0.001632000	2.522753000
H	3.361250000	1.580310000	1.522336000
C	5.661367000	-1.503207000	1.108323000
H	5.610250000	-1.087092000	-0.993897000
C	5.236354000	-1.117981000	2.378245000
H	4.073901000	0.300350000	3.510137000
H	6.300674000	-2.374711000	0.988905000
H	5.541094000	-1.688633000	3.251941000
H	3.061934000	0.550003000	-2.305082000
C	5.461996000	1.868578000	-2.348539000
H	6.035601000	0.997630000	-2.682233000
H	5.209485000	2.450775000	-3.240071000
H	6.128217000	2.479295000	-1.731610000

TS1 (E = -2491.96061396; G = -2491.227992)

Ni	0.080381000	0.333883000	-0.117895000
B	-1.637684000	-0.427855000	-0.645826000

P	-1.279868000	2.016654000	0.267983000
P	0.514780000	-1.855039000	-0.048091000
N	-2.938117000	0.096813000	-0.278018000
N	-1.911675000	-1.801785000	-1.020869000
C	-1.611565000	3.236285000	-1.173400000
C	-1.043563000	2.958009000	1.904727000
C	-0.825353000	-2.743457000	-1.013369000
H	-0.471006000	-2.986563000	-2.024174000
H	-1.116535000	-3.688814000	-0.533721000
C	-5.959959000	-2.101776000	-0.193079000
H	-7.017771000	-2.125050000	0.053664000
C	-2.999866000	1.309861000	0.487084000
H	-3.188053000	1.099204000	1.551569000
H	-3.783524000	2.004264000	0.149953000
C	-3.895254000	-0.902374000	-0.359245000
C	0.221436000	-2.471741000	1.731654000
C	-5.247223000	-0.908213000	-0.038964000
H	-5.732225000	-0.009257000	0.334373000
C	-3.971702000	-3.265228000	-0.974032000
H	-3.481745000	-4.170196000	-1.326164000
C	-3.255761000	-2.083265000	-0.828630000
C	2.056679000	-2.708409000	-0.772539000
C	-5.332988000	-3.260448000	-0.651800000
H	-5.908377000	-4.175903000	-0.759378000
C	1.146182000	-1.716272000	2.687691000
H	1.024705000	-0.634668000	2.581483000
H	0.898915000	-1.993546000	3.720289000
H	2.203232000	-1.947967000	2.529892000
C	0.416235000	-3.973821000	1.924242000
H	1.459787000	-4.280284000	1.807942000
H	0.116871000	-4.243812000	2.944958000

H	-0.197627000	-4.574973000	1.244503000
C	-1.225690000	-2.118947000	2.090413000
H	-1.420088000	-1.048340000	1.952821000
H	-1.957181000	-2.678604000	1.496584000
H	-1.402882000	-2.364026000	3.145060000
C	2.356180000	-1.986223000	-2.088653000
H	2.664619000	-0.953478000	-1.917131000
H	3.177340000	-2.495727000	-2.608894000
H	1.492660000	-1.970816000	-2.764216000
C	1.832049000	-4.190469000	-1.094527000
H	2.771260000	-4.605677000	-1.481871000
H	1.552490000	-4.788845000	-0.225717000
H	1.076999000	-4.345493000	-1.870529000
C	3.253890000	-2.577749000	0.164375000
H	4.168397000	-2.870915000	-0.366318000
H	3.393046000	-1.553717000	0.520487000
H	3.161987000	-3.234300000	1.035277000
C	0.137696000	3.916104000	1.778211000
H	0.371253000	4.347065000	2.759837000
H	1.034818000	3.395907000	1.422732000
H	-0.070195000	4.750844000	1.099476000
C	-0.695982000	1.893313000	2.949912000
H	-1.495353000	1.152214000	3.073561000
H	0.220066000	1.359998000	2.680775000
H	-0.547784000	2.377517000	3.923233000
C	-2.281801000	3.710952000	2.397654000
H	-2.079788000	4.092801000	3.406400000
H	-2.544317000	4.570312000	1.780607000
H	-3.164120000	3.067901000	2.477843000
C	-0.302812000	3.761660000	-1.754521000
H	0.277223000	2.951007000	-2.203059000

H	-0.526662000	4.480146000	-2.553041000
H	0.326653000	4.268468000	-1.018281000
C	-2.323880000	2.456152000	-2.283890000
H	-2.436930000	3.114491000	-3.154364000
H	-1.749951000	1.579380000	-2.599860000
H	-3.324629000	2.117736000	-1.999472000
C	-2.482432000	4.423842000	-0.771370000
H	-1.950735000	5.121741000	-0.118003000
H	-2.762456000	4.980356000	-1.674687000
H	-3.412495000	4.127392000	-0.275027000
H (Iso=2.014)	1.249360000	0.837142000	0.804235000
Si	2.424445000	1.698055000	-1.173164000
H	2.068053000	2.924179000	-0.431305000
C	3.881191000	0.887409000	-0.293442000
C	4.004713000	0.922877000	1.105408000
C	4.927690000	0.298394000	-1.019689000
C	5.106162000	0.368980000	1.750815000
H	3.207773000	1.373900000	1.694939000
C	6.030151000	-0.265978000	-0.380781000
H	4.879365000	0.269975000	-2.107782000
C	6.118065000	-0.238006000	1.008161000
H	5.173824000	0.405592000	2.835460000
H	6.819762000	-0.729113000	-0.967727000
H	6.974279000	-0.681100000	1.510711000
H	1.208853000	0.856394000	-1.545637000
C	3.066740000	2.215349000	-2.877063000
H	3.283497000	1.346886000	-3.509695000
H	2.325965000	2.816691000	-3.415122000
H	3.984418000	2.809165000	-2.807299000

Int1 (E = -2491.96163281; G = -2491.228552)

Ni	0.114736000	0.369324000	-0.260364000
B	-1.616980000	-0.460575000	-0.612129000
P	-1.286139000	2.009420000	0.234642000
P	0.578821000	-1.834592000	-0.061012000
N	-2.910932000	0.044763000	-0.207209000
N	-1.872219000	-1.842599000	-0.958505000
C	-1.716055000	3.204956000	-1.199872000
C	-0.980596000	2.967366000	1.849547000
C	-0.766106000	-2.759442000	-0.977897000
H	-0.435271000	-2.998170000	-1.997408000
H	-1.019221000	-3.708947000	-0.484913000
C	-5.889974000	-2.205084000	-0.016962000
H	-6.940874000	-2.243275000	0.255823000
C	-2.973954000	1.266877000	0.541236000
H	-3.113680000	1.067917000	1.615307000
H	-3.787758000	1.935054000	0.224653000
C	-3.851914000	-0.973295000	-0.249200000
C	0.346701000	-2.407158000	1.739669000
C	-5.194900000	-0.997358000	0.104872000
H	-5.686312000	-0.101993000	0.478311000
C	-3.901786000	-3.343563000	-0.834693000
H	-3.405470000	-4.243760000	-1.189931000
C	-3.204534000	-2.147307000	-0.722264000
C	2.116644000	-2.691234000	-0.799310000
C	-5.254394000	-3.358167000	-0.477297000
H	-5.816056000	-4.284617000	-0.559413000
C	1.281326000	-1.587180000	2.630248000
H	1.141095000	-0.515607000	2.455595000
H	1.057226000	-1.796718000	3.683627000
H	2.337999000	-1.815977000	2.466237000
C	0.581383000	-3.896991000	1.973904000

H	1.623484000	-4.190913000	1.822001000
H	0.329356000	-4.139406000	3.014010000
H	-0.050400000	-4.528508000	1.339430000
C	-1.096697000	-2.080189000	2.134211000
H	-1.326254000	-1.020848000	1.970740000
H	-1.831430000	-2.679375000	1.584624000
H	-1.231998000	-2.294906000	3.201591000
C	2.399848000	-1.981238000	-2.124950000
H	2.726420000	-0.952150000	-1.965258000
H	3.205386000	-2.503588000	-2.656461000
H	1.524884000	-1.956155000	-2.785142000
C	1.877572000	-4.175639000	-1.101929000
H	2.814433000	-4.605628000	-1.478232000
H	1.587299000	-4.758370000	-0.225624000
H	1.125133000	-4.333638000	-1.879536000
C	3.333842000	-2.566129000	0.113802000
H	4.232266000	-2.872476000	-0.436647000
H	3.496789000	-1.543481000	0.460226000
H	3.256038000	-3.217272000	0.989803000
C	0.158154000	3.964635000	1.656451000
H	0.434219000	4.399045000	2.625344000
H	1.050708000	3.482208000	1.242648000
H	-0.121493000	4.793731000	0.997771000
C	-0.544637000	1.923053000	2.881365000
H	-1.295174000	1.136719000	3.028544000
H	0.390818000	1.441422000	2.584466000
H	-0.391266000	2.413605000	3.850627000
C	-2.215110000	3.685948000	2.400776000
H	-1.963535000	4.106688000	3.382440000
H	-2.551292000	4.514722000	1.777539000
H	-3.063169000	3.011837000	2.557677000

C	-0.459471000	3.790723000	-1.837123000
H	0.150873000	3.011244000	-2.299217000
H	-0.758468000	4.484784000	-2.632611000
H	0.169833000	4.344127000	-1.134999000
C	-2.427272000	2.374816000	-2.274175000
H	-2.603395000	3.012803000	-3.149017000
H	-1.816877000	1.524805000	-2.597074000
H	-3.398080000	1.989113000	-1.948841000
C	-2.627745000	4.353413000	-0.773258000
H	-2.104036000	5.085296000	-0.150936000
H	-2.970415000	4.884142000	-1.670357000
H	-3.522028000	4.020329000	-0.236320000
H (Iso=2.014)	1.310220000	0.972073000	0.559103000
Si	2.152308000	1.545195000	-1.127523000
H	1.914918000	2.921282000	-0.626062000
C	3.727919000	0.906430000	-0.265152000
C	3.857073000	0.945032000	1.133072000
C	4.836109000	0.451139000	-0.995646000
C	5.023709000	0.537003000	1.772705000
H	3.013022000	1.286434000	1.733117000
C	6.002902000	0.022239000	-0.364595000
H	4.785560000	0.414446000	-2.082754000
C	6.098933000	0.061316000	1.023293000
H	5.092441000	0.580575000	2.857290000
H	6.838142000	-0.342078000	-0.958453000
H	7.006876000	-0.272709000	1.519550000
H	0.878669000	0.760615000	-1.718714000
C	2.774312000	1.855730000	-2.912562000
H	2.983174000	0.924457000	-3.454245000
H	2.007249000	2.380943000	-3.494128000
H	3.683532000	2.467427000	-2.943682000

Int1' (E = -2491.95411277; G = -2491.220632)

Ni	-0.079214000	0.229463000	0.529865000
B	1.788440000	-0.323394000	0.406315000
P	0.951471000	2.122486000	-0.016020000
P	-0.228681000	-2.026264000	0.309215000
N	2.902431000	0.443717000	-0.107932000
N	2.328357000	-1.657897000	0.538536000
C	1.368657000	3.256755000	1.460900000
C	0.229056000	3.087499000	-1.488105000
C	1.436363000	-2.764048000	0.711623000
H	1.451360000	-3.163364000	1.734113000
H	1.674531000	-3.594284000	0.030364000
C	6.160757000	-1.178501000	-0.949307000
H	7.150846000	-0.994895000	-1.357157000
C	2.668872000	1.752059000	-0.638626000
H	2.693809000	1.750656000	-1.738475000
H	3.399867000	2.498899000	-0.297529000
C	3.998399000	-0.378207000	-0.315545000
C	-0.449476000	-2.490327000	-1.521679000
C	5.257669000	-0.115946000	-0.838891000
H	5.527958000	0.886960000	-1.160586000
C	4.542325000	-2.740802000	-0.021908000
H	4.268187000	-3.746485000	0.287808000
C	3.640508000	-1.690520000	0.095176000
C	-1.326633000	-3.120538000	1.416165000
C	5.808628000	-2.467675000	-0.549704000
H	6.528490000	-3.275264000	-0.649191000
C	-1.685382000	-1.770603000	-2.057526000
H	-1.592044000	-0.688056000	-1.928802000
H	-1.796733000	-1.978149000	-3.129183000

H	-2.611710000	-2.080690000	-1.563115000
C	-0.575083000	-3.988040000	-1.785863000
H	-1.520515000	-4.396499000	-1.417506000
H	-0.553599000	-4.161162000	-2.869045000
H	0.246697000	-4.569330000	-1.352815000
C	0.780729000	-1.959729000	-2.264816000
H	0.925910000	-0.886285000	-2.091981000
H	1.702826000	-2.477402000	-1.977934000
H	0.640058000	-2.112663000	-3.341837000
C	-1.149930000	-2.580173000	2.839483000
H	-1.422746000	-1.527572000	2.927544000
H	-1.784470000	-3.156414000	3.523847000
H	-0.116246000	-2.685707000	3.187951000
C	-0.903572000	-4.594759000	1.433314000
H	-1.552169000	-5.122524000	2.144012000
H	-1.016855000	-5.093841000	0.470340000
H	0.123036000	-4.745380000	1.778983000
C	-2.789343000	-3.047428000	0.985565000
H	-3.421852000	-3.502316000	1.758263000
H	-3.146078000	-2.026608000	0.835352000
H	-2.964078000	-3.601270000	0.056734000
C	-1.093078000	3.736987000	-1.095069000
H	-1.572119000	4.168518000	-1.982476000
H	-1.789195000	3.006800000	-0.670627000
H	-0.958031000	4.548308000	-0.371599000
C	-0.030049000	2.046857000	-2.583268000
H	0.896908000	1.597314000	-2.957163000
H	-0.675847000	1.236195000	-2.231723000
H	-0.520622000	2.534856000	-3.434436000
C	1.171516000	4.149795000	-2.062104000
H	0.750548000	4.512828000	-3.007865000

H	1.284995000	5.018096000	-1.412282000
H	2.169530000	3.762138000	-2.291269000
C	0.121406000	3.544759000	2.285906000
H	-0.319828000	2.618646000	2.660189000
H	0.395692000	4.162452000	3.149944000
H	-0.643960000	4.088870000	1.725405000
C	2.340661000	2.467119000	2.345212000
H	2.538359000	3.050245000	3.252759000
H	1.916636000	1.504883000	2.651109000
H	3.304772000	2.275722000	1.864327000
C	2.013126000	4.580387000	1.055232000
H	1.289067000	5.267224000	0.607994000
H	2.403459000	5.072272000	1.954914000
H	2.853664000	4.466083000	0.362492000
H (Iso=2.014)	-1.437558000	0.775298000	-0.148757000
Si	-2.382197000	0.681308000	1.284183000
H	-2.906100000	-0.297355000	2.300191000
C	-3.821468000	0.378061000	0.012302000
C	-3.784626000	0.820189000	-1.316227000
C	-5.008841000	-0.231090000	0.444106000
C	-4.869061000	0.664160000	-2.177073000
H	-2.871969000	1.285301000	-1.696996000
C	-6.100888000	-0.401414000	-0.404841000
H	-5.069895000	-0.593438000	1.472758000
C	-6.032299000	0.046803000	-1.722254000
H	-4.806409000	1.016902000	-3.204538000
H	-7.004523000	-0.887024000	-0.041942000
H	-6.878607000	-0.087205000	-2.391744000
H	-0.840638000	0.576341000	2.021397000
C	-2.916056000	2.375018000	1.947027000
H	-4.005048000	2.339297000	2.064508000

H	-2.485080000	2.607727000	2.924589000
H	-2.700812000	3.212081000	1.276923000

TS2 (E = -2491.95014654; G = -2491.21683)

Ni	-0.045567000	0.122851000	0.443472000
B	1.764493000	-0.223804000	-0.145549000
P	0.770020000	2.169067000	0.112647000
P	0.015135000	-2.133987000	0.347976000
N	2.769023000	0.705170000	-0.605697000
N	2.476224000	-1.476970000	-0.070815000
C	1.233556000	3.066692000	1.728615000
C	-0.160397000	3.271899000	-1.130865000
C	1.809192000	-2.644165000	0.411877000
H	2.101287000	-2.882335000	1.443615000
H	2.002717000	-3.535711000	-0.202255000
C	6.284150000	-0.379127000	-1.258000000
H	7.262466000	-0.031839000	-1.578504000
C	2.447834000	2.092711000	-0.719070000
H	2.399080000	2.428153000	-1.763804000
H	3.177684000	2.736430000	-0.209213000
C	3.982896000	0.062612000	-0.777935000
C	-0.515233000	-2.783138000	-1.365855000
C	5.223998000	0.530998000	-1.188797000
H	5.363432000	1.577015000	-1.451422000
C	4.858627000	-2.202606000	-0.507038000
H	4.720596000	-3.247501000	-0.239570000
C	3.799686000	-1.306618000	-0.435368000
C	-0.711552000	-3.264412000	1.711837000
C	6.104194000	-1.721670000	-0.924714000
H	6.944735000	-2.407351000	-0.986579000
C	-1.931705000	-2.300447000	-1.672850000

H	-1.987686000	-1.208364000	-1.682792000
H	-2.228061000	-2.654630000	-2.668082000
H	-2.678940000	-2.666801000	-0.962745000
C	-0.448200000	-4.304377000	-1.497245000
H	-1.168651000	-4.822323000	-0.859769000
H	-0.683305000	-4.579185000	-2.532961000
H	0.548998000	-4.704329000	-1.284169000
C	0.434185000	-2.177588000	-2.404342000
H	0.436668000	-1.082589000	-2.367662000
H	1.466370000	-2.521870000	-2.279308000
H	0.102377000	-2.482551000	-3.404461000
C	-0.545043000	-2.496920000	3.025624000
H	-1.148269000	-1.586151000	3.046155000
H	-0.850049000	-3.137660000	3.862482000
H	0.497357000	-2.200945000	3.196603000
C	0.058176000	-4.587201000	1.832984000
H	-0.452897000	-5.213504000	2.574586000
H	0.094433000	-5.158449000	0.902611000
H	1.081928000	-4.452569000	2.191578000
C	-2.187812000	-3.608007000	1.498224000
H	-2.557049000	-4.121142000	2.395086000
H	-2.818828000	-2.731512000	1.344742000
H	-2.340546000	-4.290336000	0.657290000
C	-1.549863000	3.643851000	-0.626704000
H	-2.069975000	4.228911000	-1.394964000
H	-2.164219000	2.761057000	-0.428512000
H	-1.517018000	4.256294000	0.280196000
C	-0.313910000	2.427278000	-2.402799000
H	0.646919000	2.169651000	-2.861639000
H	-0.848927000	1.492231000	-2.209392000
H	-0.885151000	3.000288000	-3.143508000

C	0.597960000	4.554438000	-1.485080000
H	0.110134000	5.020330000	-2.350277000
H	0.581440000	5.288482000	-0.677679000
H	1.642038000	4.382506000	-1.764918000
C	0.026283000	3.188932000	2.650334000
H	-0.405679000	2.209168000	2.866999000
H	0.343473000	3.639948000	3.598665000
H	-0.758436000	3.827329000	2.233649000
C	2.257799000	2.149050000	2.409800000
H	2.488484000	2.553214000	3.402807000
H	1.860030000	1.135818000	2.539636000
H	3.203369000	2.078081000	1.861198000
C	1.844199000	4.453026000	1.525895000
H	1.087913000	5.199548000	1.268372000
H	2.301564000	4.778159000	2.468449000
H	2.629255000	4.484102000	0.762868000
H (Iso=2.014)	-1.524563000	0.471999000	-0.320990000
Si	-2.457886000	0.364465000	0.993011000
H	-2.731303000	-0.891971000	1.735540000
C	-3.979878000	0.281362000	-0.209504000
C	-3.939348000	0.730079000	-1.535685000
C	-5.202554000	-0.237598000	0.244746000
C	-5.056815000	0.684305000	-2.368139000
H	-2.998454000	1.117329000	-1.934246000
C	-6.327887000	-0.295764000	-0.574452000
H	-5.274132000	-0.612624000	1.268012000
C	-6.258147000	0.170539000	-1.886155000
H	-4.990668000	1.043619000	-3.393100000
H	-7.260399000	-0.707158000	-0.193611000
H	-7.133252000	0.127431000	-2.529914000
H	-0.808141000	0.371658000	1.868017000

C	-3.004505000	1.794084000	2.112451000
H	-4.094133000	1.749262000	2.221714000
H	-2.558805000	1.716948000	3.108539000
H	-2.766772000	2.786223000	1.720502000

Int2 (E = -2491.95418606; G = -2491.221671)

Ni	-0.068504000	-0.025810000	0.395908000
B	1.624202000	0.014846000	-0.566650000
P	0.362337000	2.167023000	0.336241000
P	0.484950000	-2.179255000	0.391303000
N	2.397887000	1.173973000	-0.961718000
N	2.549442000	-1.086731000	-0.747818000
C	0.798511000	2.887358000	2.040797000
C	-0.809117000	3.274878000	-0.685803000
C	2.274803000	-2.345080000	-0.121474000
H	2.912610000	-2.491296000	0.761679000
H	2.437658000	-3.207559000	-0.782712000
C	5.996402000	0.883397000	-1.920987000
H	6.870865000	1.455035000	-2.218911000
C	1.954137000	2.488088000	-0.606572000
H	1.788830000	3.134408000	-1.478002000
H	2.694267000	2.996395000	0.027114000
C	3.687828000	0.797397000	-1.300006000
C	-0.336731000	-3.231727000	-0.978855000
C	4.790905000	1.551019000	-1.680432000
H	4.716586000	2.631087000	-1.782074000
C	4.987138000	-1.267006000	-1.395184000
H	5.066025000	-2.344686000	-1.273960000
C	3.784993000	-0.612527000	-1.159188000
C	0.526491000	-3.072137000	2.071918000
C	6.092838000	-0.501130000	-1.779907000

H	7.042098000	-0.995172000	-1.968260000
C	-1.860813000	-3.172925000	-0.939014000
H	-2.239418000	-2.166075000	-1.133380000
H	-2.254650000	-3.820903000	-1.731948000
H	-2.283736000	-3.518912000	0.007693000
C	0.082924000	-4.700996000	-0.933903000
H	-0.402581000	-5.235182000	-0.112037000
H	-0.236716000	-5.188335000	-1.863359000
H	1.163978000	-4.849483000	-0.848089000
C	0.110670000	-2.609218000	-2.307139000
H	-0.163264000	-1.549371000	-2.363300000
H	1.188105000	-2.687338000	-2.481564000
H	-0.394473000	-3.132125000	-3.128175000
C	1.212453000	-2.092511000	3.031441000
H	0.646674000	-1.159382000	3.116626000
H	1.280353000	-2.550182000	4.026112000
H	2.232340000	-1.839333000	2.718599000
C	1.322835000	-4.381015000	2.065929000
H	1.436507000	-4.721040000	3.102767000
H	0.821978000	-5.183528000	1.523042000
H	2.332995000	-4.273792000	1.658677000
C	-0.881695000	-3.346645000	2.588790000
H	-0.818839000	-3.805832000	3.583211000
H	-1.462332000	-2.425160000	2.684590000
H	-1.438099000	-4.038064000	1.946323000
C	-2.237254000	3.252147000	-0.156985000
H	-2.862117000	3.899805000	-0.783875000
H	-2.678812000	2.253460000	-0.202088000
H	-2.313525000	3.623523000	0.869270000
C	-0.815070000	2.663451000	-2.093702000
H	0.140211000	2.778982000	-2.614668000

H	-1.054557000	1.595194000	-2.068983000
H	-1.579336000	3.167129000	-2.697979000
C	-0.350245000	4.732656000	-0.769505000
H	-0.900886000	5.229090000	-1.578164000
H	-0.566748000	5.285515000	0.147816000
H	0.715420000	4.849478000	-0.989688000
C	-0.385398000	2.762583000	2.994498000
H	-0.735839000	1.728250000	3.062378000
H	-0.076851000	3.082722000	3.997390000
H	-1.229336000	3.394872000	2.699702000
C	1.923051000	1.982774000	2.562085000
H	2.184671000	2.287795000	3.582643000
H	1.603992000	0.934487000	2.588118000
H	2.837112000	2.042485000	1.960905000
C	1.278316000	4.337853000	2.023465000
H	0.457846000	5.042742000	1.871203000
H	1.725781000	4.575615000	2.996509000
H	2.041362000	4.537901000	1.264751000
H (Iso=2.014)	-1.575508000	-0.004234000	-0.462405000
Si	-2.577337000	-0.230771000	0.754212000
H	-2.674807000	-1.633511000	1.216189000
C	-4.047908000	-0.144712000	-0.501218000
C	-3.843526000	0.091586000	-1.867783000
C	-5.375260000	-0.346543000	-0.087299000
C	-4.898978000	0.140411000	-2.777530000
H	-2.823270000	0.237223000	-2.230912000
C	-6.439586000	-0.308505000	-0.984014000
H	-5.585287000	-0.546897000	0.964138000
C	-6.203850000	-0.058288000	-2.334988000
H	-4.703615000	0.331675000	-3.830554000
H	-7.455609000	-0.472673000	-0.631172000

H	-7.032897000	-0.021855000	-3.037564000
H	-0.900824000	-0.089446000	1.758128000
C	-3.219018000	0.858606000	2.167056000
H	-4.314178000	0.835683000	2.201706000
H	-2.837876000	0.487610000	3.124511000
H	-2.922144000	1.905808000	2.087539000

Phosphine decoordination

Complex 5-D (E = -1928.25615974; G = -1927.660463)

Ni	-0.000065000	-1.029171000	0.000027000
B	0.000067000	0.890119000	0.000130000
P	-2.123327000	-0.639440000	-0.055329000
P	2.123250000	-0.639758000	0.055331000
N	-1.125923000	1.765389000	-0.173297000
N	1.126190000	1.765193000	0.173693000
C	-2.889017000	-0.874424000	1.657228000
C	-3.094618000	-1.496214000	-1.427178000
C	2.425039000	1.196011000	0.406811000
H	2.753211000	1.338927000	1.443675000
H	3.199551000	1.615544000	-0.247626000
C	-0.688136000	5.474847000	-0.112763000
H	-1.217412000	6.419551000	-0.200781000
C	-2.424862000	1.196435000	-0.406502000
H	-2.753020000	1.339604000	-1.443337000
H	-3.199303000	1.615944000	0.248033000
C	-0.705234000	3.080898000	-0.113284000
C	2.888701000	-0.874535000	-1.657382000
C	-1.400807000	4.277676000	-0.229551000
H	-2.472688000	4.278767000	-0.408179000
C	1.401446000	4.277425000	0.230392000
H	2.473328000	4.278323000	0.409019000

C	0.705694000	3.080774000	0.113901000
C	3.094599000	-1.496919000	1.426896000
C	0.688954000	5.474725000	0.113829000
H	1.218370000	6.419334000	0.202019000
C	2.515273000	-2.264646000	-2.184654000
H	1.433052000	-2.422648000	-2.150526000
H	2.852249000	-2.354979000	-3.225375000
H	2.982694000	-3.069926000	-1.613769000
C	4.405874000	-0.695005000	-1.691713000
H	4.920666000	-1.492530000	-1.148346000
H	4.751255000	-0.734773000	-2.732619000
H	4.725987000	0.267726000	-1.279054000
C	2.222526000	0.167684000	-2.568122000
H	1.132574000	0.060444000	-2.563672000
H	2.459085000	1.197721000	-2.284759000
H	2.576621000	0.018671000	-3.595899000
C	2.184943000	-1.365007000	2.657443000
H	1.218744000	-1.849811000	2.479207000
H	2.661356000	-1.847462000	3.520421000
H	1.996705000	-0.318890000	2.925218000
C	4.459788000	-0.876871000	1.735674000
H	4.912535000	-1.416495000	2.577344000
H	5.154299000	-0.944294000	0.895623000
H	4.386595000	0.173583000	2.032666000
C	3.260355000	-2.980805000	1.095692000
H	3.612454000	-3.512987000	1.988358000
H	2.310278000	-3.426720000	0.784353000
H	4.001616000	-3.142135000	0.306668000
C	-3.260664000	-2.980125000	-1.096212000
H	-3.612784000	-3.512107000	-1.988987000
H	-2.310693000	-3.426225000	-0.784828000

H	-4.002017000	-3.141441000	-0.307274000
C	-2.184739000	-1.364259000	-2.657560000
H	-1.996390000	-0.318130000	-2.925213000
H	-1.218613000	-1.849165000	-2.479198000
H	-2.661045000	-1.846607000	-3.520655000
C	-4.459666000	-0.875923000	-1.736053000
H	-4.912150000	-1.415011000	-2.578208000
H	-5.154445000	-0.943889000	-0.896263000
H	-4.386391000	0.174721000	-2.032347000
C	-2.516172000	-2.264856000	2.184073000
H	-1.434032000	-2.423376000	2.149751000
H	-2.853061000	-2.355330000	3.224810000
H	-2.984077000	-3.069733000	1.613007000
C	-2.222450000	0.167249000	2.568313000
H	-2.576646000	0.018071000	3.596029000
H	-1.132542000	0.059567000	2.563878000
H	-2.458577000	1.197470000	2.285255000
C	-4.406118000	-0.694282000	1.691524000
H	-4.921224000	-1.491463000	1.147952000
H	-4.751559000	-0.734151000	2.732405000
H	-4.725831000	0.268669000	1.279062000
H (Iso=2.014)	-0.000255000	-2.588728000	-0.000053000

TS P decoordination (E = -1928.19967647; G = -1927.602686)

Ni	0.503849000	0.025102000	-0.201743000
B	-1.244035000	0.923535000	-0.531520000
N	-2.601270000	0.519269000	-0.843418000
N	-1.314393000	2.372211000	-0.558402000
C	-3.390891000	1.644493000	-1.044778000
C	-4.736375000	1.778376000	-1.372876000
H	-5.365155000	0.908415000	-1.540119000

C	-5.269640000	3.064921000	-1.497593000
H	-6.319445000	3.180118000	-1.752757000
C	-4.477568000	4.197601000	-1.308166000
H	-4.915773000	5.185957000	-1.414937000
C	-3.122393000	4.076320000	-0.989648000
H	-2.498154000	4.954761000	-0.849983000
C	-2.588500000	2.800240000	-0.860711000
P	1.059791000	1.952430000	0.392904000
C	-0.174434000	3.191124000	-0.271417000
H	-0.394753000	3.978657000	0.461426000
H	0.220408000	3.678075000	-1.172033000
C	2.707114000	2.591786000	-0.265012000
C	2.732726000	2.146624000	-1.734689000
H	1.901720000	2.568299000	-2.311498000
H	2.682551000	1.055366000	-1.811925000
H	3.665857000	2.486192000	-2.201379000
C	2.842164000	4.117691000	-0.199535000
H	3.798624000	4.402837000	-0.654958000
H	2.842629000	4.500497000	0.822919000
H	2.057052000	4.635574000	-0.758265000
C	3.879515000	1.940094000	0.469239000
H	3.779612000	0.850784000	0.489674000
H	3.975454000	2.305055000	1.496103000
H	4.811153000	2.187546000	-0.054472000
C	0.896313000	2.099483000	2.268400000
C	1.426723000	3.426564000	2.812566000
H	0.957111000	4.292545000	2.334047000
H	2.510908000	3.514012000	2.701271000
H	1.203551000	3.486259000	3.884942000
C	-0.596127000	1.970810000	2.607519000
H	-0.707519000	1.993577000	3.698708000

H	-1.012883000	1.027302000	2.241018000
H	-1.198711000	2.787627000	2.198839000
C	1.633485000	0.928811000	2.928262000
H	2.709240000	0.946466000	2.739177000
H	1.248511000	-0.030288000	2.568167000
H	1.481372000	0.979425000	4.013802000
P	-2.563615000	-1.908821000	0.504165000
C	-3.140909000	-0.817109000	-0.906424000
H	-2.934568000	-1.282486000	-1.879587000
H	-4.227744000	-0.758074000	-0.819113000
C	-1.305251000	-3.045963000	-0.350612000
C	-0.181738000	-2.132709000	-0.866062000
H	0.601188000	-2.734170000	-1.340250000
H	0.349977000	-1.701527000	0.027373000
H	-0.520101000	-1.388672000	-1.600865000
C	-1.825857000	-3.894358000	-1.509542000
H	-0.988507000	-4.404345000	-2.004353000
H	-2.338977000	-3.298186000	-2.270942000
H	-2.512772000	-4.670256000	-1.160804000
C	-0.668267000	-3.957532000	0.708356000
H	-1.355822000	-4.722376000	1.074901000
H	-0.312213000	-3.380776000	1.569230000
H	0.195746000	-4.476409000	0.273700000
C	-4.180978000	-2.864753000	0.836170000
C	-5.021854000	-1.871447000	1.655344000
H	-5.240129000	-0.948311000	1.105813000
H	-4.517232000	-1.590347000	2.585460000
H	-5.984352000	-2.330189000	1.916333000
C	-4.999164000	-3.316545000	-0.377878000
H	-5.929555000	-3.784679000	-0.029525000
H	-4.476577000	-4.054802000	-0.989546000

H	-5.290301000	-2.486031000	-1.028750000
C	-3.889279000	-4.081226000	1.718135000
H	-4.829115000	-4.469025000	2.132055000
H	-3.234574000	-3.831659000	2.560563000
H	-3.427789000	-4.895277000	1.151372000
H (Iso=2.014)	1.958362000	-0.420306000	0.069355000

Int after decoordination (E = -1928.20036253; G = -1927.604845)

Ni	0.519662000	0.040243000	-0.154510000
B	-1.233425000	0.929994000	-0.447355000
N	-2.571900000	0.503566000	-0.796989000
N	-1.345259000	2.373808000	-0.395926000
C	-3.402188000	1.609367000	-0.916645000
C	-4.755990000	1.720496000	-1.219104000
H	-5.363621000	0.842343000	-1.418889000
C	-5.328072000	2.995104000	-1.269775000
H	-6.384862000	3.091550000	-1.502973000
C	-4.567080000	4.139879000	-1.031248000
H	-5.035577000	5.118773000	-1.081135000
C	-3.205807000	4.042410000	-0.731624000
H	-2.606045000	4.930275000	-0.550332000
C	-2.633789000	2.777927000	-0.671575000
P	1.100466000	1.981050000	0.375778000
C	-0.207691000	3.210636000	-0.156902000
H	-0.392178000	3.971601000	0.612945000
H	0.111259000	3.732812000	-1.067832000
C	2.669074000	2.646670000	-0.431058000
C	2.556825000	2.208593000	-1.898784000
H	1.670901000	2.626544000	-2.390354000
H	2.507742000	1.117298000	-1.977550000
H	3.437827000	2.557707000	-2.451681000

C	2.794171000	4.173617000	-0.368859000
H	3.694779000	4.471600000	-0.919956000
H	2.900268000	4.549691000	0.650865000
H	1.950536000	4.688844000	-0.837193000
C	3.916456000	2.009153000	0.182218000
H	3.833721000	0.918696000	0.212040000
H	4.110739000	2.377202000	1.194232000
H	4.787920000	2.268498000	-0.431706000
C	1.107961000	2.118874000	2.260088000
C	1.634223000	3.465280000	2.759398000
H	1.100065000	4.315609000	2.322375000
H	2.702590000	3.586941000	2.561187000
H	1.497265000	3.519452000	3.846498000
C	-0.343255000	1.931658000	2.726298000
H	-0.364116000	1.950836000	3.823026000
H	-0.753225000	0.973005000	2.393022000
H	-1.010602000	2.722178000	2.370754000
C	1.947544000	0.981443000	2.851669000
H	2.999835000	1.041083000	2.565363000
H	1.572546000	0.005833000	2.528825000
H	1.892793000	1.031276000	3.946518000
P	-2.723241000	-1.931415000	0.518587000
C	-3.023939000	-0.852036000	-0.986541000
H	-2.549570000	-1.288862000	-1.873616000
H	-4.096266000	-0.848220000	-1.186526000
C	-1.311756000	-3.056681000	-0.077815000
C	-0.090059000	-2.149513000	-0.319481000
H	0.784864000	-2.763832000	-0.547497000
H	0.148982000	-1.612448000	0.629208000
H	-0.244290000	-1.454839000	-1.177834000
C	-1.572064000	-3.892422000	-1.330082000

H	-0.643841000	-4.385445000	-1.648459000
H	-1.926550000	-3.293201000	-2.175098000
H	-2.305004000	-4.680961000	-1.139051000
C	-0.916911000	-3.985623000	1.080075000
H	-1.674839000	-4.745148000	1.280216000
H	-0.745905000	-3.424558000	2.005414000
H	0.013669000	-4.511336000	0.830744000
C	-4.367006000	-2.895665000	0.564412000
C	-5.350347000	-1.879821000	1.169408000
H	-5.454394000	-0.975614000	0.559080000
H	-5.036812000	-1.565845000	2.170237000
H	-6.346173000	-2.334229000	1.251571000
C	-4.929367000	-3.395943000	-0.769195000
H	-5.910991000	-3.857252000	-0.596489000
H	-4.294351000	-4.152601000	-1.234305000
H	-5.081977000	-2.592538000	-1.496302000
C	-4.255776000	-4.078286000	1.530530000
H	-5.260331000	-4.447037000	1.775177000
H	-3.767404000	-3.798703000	2.470663000
H	-3.705789000	-4.915756000	1.091410000
H (Iso=2.014)	1.993554000	-0.384116000	0.028531000

Hydridoborate adducts

HBcat (E = -406.997380896; G = -406.92463)

B	2.293465000	0.000296000	-0.002155000
O	3.065172000	-1.139814000	-0.125439000
O	3.065159000	1.140398000	0.121310000
C	4.364968000	-0.691567000	-0.076160000
C	4.364960000	0.691988000	0.073668000
C	5.534690000	-1.422464000	-0.154713000
C	5.534668000	1.422771000	0.153458000

H	5.523651000	-2.500716000	-0.272525000
C	6.725603000	-0.694160000	-0.075444000
C	6.725593000	0.694348000	0.075494000
H	5.523605000	2.501033000	0.271182000
H	7.672159000	-1.223083000	-0.133134000
H	7.672142000	1.223175000	0.134155000
H	1.118257000	0.000393000	-0.003167000

Hbpin (E = -411.834515153; G = -411.676058)

B	2.342771000	-0.012342000	-0.006625000
O	3.087609000	-1.007568000	-0.568777000
O	3.072862000	0.991196000	0.560283000
C	4.468603000	-0.764579000	-0.162778000
C	4.459251000	0.763206000	0.163688000
H	1.158304000	-0.018459000	-0.010874000
C	5.362236000	1.175897000	1.306280000
H	6.409022000	0.952480000	1.074500000
H	5.282015000	2.253086000	1.476829000
H	5.100312000	0.668637000	2.236930000
C	4.719246000	-1.633601000	1.059637000
H	4.510234000	-2.677443000	0.811513000
H	5.758172000	-1.565808000	1.396637000
H	4.069941000	-1.350896000	1.893849000
C	5.383554000	-1.167283000	-1.299423000
H	6.426343000	-0.932684000	-1.060842000
H	5.315982000	-2.245225000	-1.470732000
H	5.122205000	-0.662510000	-2.231608000
C	4.708855000	1.634745000	-1.057140000
H	4.488492000	2.676507000	-0.810162000
H	5.750382000	1.576940000	-1.387879000
H	4.067256000	1.346094000	-1.895243000

Complex NiH·HBcat (E = -2336.75936364; G = -2336.064838)

Ni	0.028720000	-0.000159000	0.000057000
B	-1.887432000	-0.000233000	0.000450000
N	-2.777265000	1.130434000	-0.087993000
N	-2.777173000	-1.130943000	0.089351000
C	-4.095380000	0.708076000	-0.064422000
C	-5.289819000	1.413923000	-0.136632000
H	-5.290510000	2.495840000	-0.243571000
C	-6.486304000	0.694039000	-0.067491000
H	-7.431225000	1.227477000	-0.121681000
C	-6.486243000	-0.694701000	0.070674000
H	-7.431119000	-1.228171000	0.125326000
C	-5.289696000	-1.414537000	0.139233000
H	-5.290291000	-2.496454000	0.246174000
C	-4.095321000	-0.708643000	0.066436000
P	-0.414441000	-2.208465000	0.040020000
C	-2.250771000	-2.441812000	0.306505000
H	-2.667020000	-3.193806000	-0.379018000
H	-2.449550000	-2.789073000	1.328334000
C	0.275557000	-3.277731000	1.453913000
C	0.174758000	-2.397974000	2.704400000
H	-0.850573000	-2.051479000	2.886250000
H	0.815038000	-1.513423000	2.631138000
H	0.488023000	-2.974447000	3.583208000
C	-0.528865000	-4.561140000	1.696395000
H	-0.042291000	-5.116510000	2.507306000
H	-0.556992000	-5.223624000	0.828851000
H	-1.557015000	-4.376758000	2.018598000
C	1.730993000	-3.665597000	1.212668000
H	2.364263000	-2.808486000	0.976832000
H	1.824292000	-4.393685000	0.399612000
H	2.123844000	-4.144504000	2.118145000
C	-0.148990000	-3.004416000	-1.661623000

C	-0.467416000	-4.497489000	-1.683102000
H	-1.467249000	-4.730222000	-1.300637000
H	0.264359000	-5.080553000	-1.115889000
H	-0.429375000	-4.855638000	-2.719231000
C	-1.086401000	-2.262869000	-2.621413000
H	-0.938418000	-2.653194000	-3.635498000
H	-0.871215000	-1.187810000	-2.640204000
H	-2.145624000	-2.389847000	-2.374107000
C	1.286538000	-2.788839000	-2.137617000
H	2.028349000	-3.266310000	-1.493515000
H	1.539727000	-1.727264000	-2.196761000
H	1.391548000	-3.216500000	-3.142452000
P	-0.414597000	2.208218000	-0.039562000
C	-2.251055000	2.441345000	-0.305349000
H	-2.667139000	3.193305000	0.380312000
H	-2.450236000	2.788572000	-1.327110000
C	-0.148648000	3.004031000	1.662041000
C	-1.085624000	2.262296000	2.622110000
H	-0.937249000	2.652484000	3.636191000
H	-0.870383000	1.187245000	2.640653000
H	-2.144951000	2.389263000	2.375252000
C	-0.467239000	4.497069000	1.683771000
H	-0.428705000	4.855165000	2.719899000
H	-1.467304000	4.729688000	1.301841000
H	0.264152000	5.080270000	1.116199000
C	1.287089000	2.788560000	2.137464000
H	2.028597000	3.266157000	1.493103000
H	1.540402000	1.727000000	2.196416000
H	1.392451000	3.216146000	3.142294000
C	0.274620000	3.277876000	-1.453569000
C	0.173770000	2.398218000	-2.704118000
H	-0.851493000	2.051389000	-2.885715000
H	0.814360000	1.513872000	-2.631121000

H	0.486592000	2.974883000	-3.582956000
C	-0.530368000	4.561012000	-1.695628000
H	-0.044168000	5.116740000	-2.506518000
H	-0.558593000	5.223310000	-0.827941000
H	-1.558504000	4.376288000	-2.017672000
C	1.729973000	3.666310000	-1.212738000
H	2.122282000	4.145646000	-2.118222000
H	2.363683000	2.809401000	-0.977378000
H	1.823244000	4.394207000	-0.399506000
H	1.473485000	0.092695000	-1.025704000
B	2.228491000	0.000008000	-0.000569000
O	3.120458000	-1.166421000	-0.135201000
O	3.119971000	1.166846000	0.133717000
C	4.387481000	-0.696211000	-0.077359000
C	4.387187000	0.697222000	0.075372000
C	5.568225000	-1.412778000	-0.151473000
C	5.567608000	1.414369000	0.149020000
H	5.557869000	-2.493040000	-0.267115000
C	6.770020000	-0.690710000	-0.074649000
C	6.769720000	0.692890000	0.071717000
H	5.556755000	2.494627000	0.264662000
H	7.714257000	-1.225642000	-0.131404000
H	7.713722000	1.228278000	0.128080000
H	1.473992000	-0.093019000	1.024933000

Complex NiH·Hbpin (E = -2341.59230007; G = -2340.810869)

Ni	0.112103000	-0.006083000	0.190646000
B	-1.796414000	-0.001855000	0.381447000
N	-2.705611000	1.073567000	0.068527000
N	-2.661763000	-1.124925000	0.650255000
C	-4.012358000	0.614258000	0.091404000
C	-5.216873000	1.248351000	-0.185748000
H	-5.238647000	2.295257000	-0.477945000

C	-6.395152000	0.500197000	-0.091460000
H	-7.346836000	0.978532000	-0.306185000
C	-6.367479000	-0.846821000	0.270416000
H	-7.297735000	-1.404521000	0.334492000
C	-5.160464000	-1.494478000	0.553053000
H	-5.138604000	-2.543784000	0.836879000
C	-3.984651000	-0.760127000	0.463099000
P	-0.377015000	-2.181987000	0.052063000
C	-2.091352000	-2.435436000	0.754789000
H	-2.657094000	-3.186208000	0.183643000
H	-2.033701000	-2.785493000	1.793748000
C	0.598201000	-3.541422000	0.964072000
C	1.002328000	-2.928036000	2.307975000
H	0.139200000	-2.536165000	2.860326000
H	1.722241000	-2.117910000	2.171775000
H	1.468000000	-3.701595000	2.931946000
C	-0.254898000	-4.786832000	1.235387000
H	0.380370000	-5.536064000	1.724137000
H	-0.654120000	-5.249769000	0.329689000
H	-1.089104000	-4.593204000	1.915864000
C	1.851284000	-3.960861000	0.198129000
H	2.473145000	-3.104327000	-0.067796000
H	1.611024000	-4.534566000	-0.702171000
H	2.453862000	-4.614237000	0.842761000
C	-0.606108000	-2.641924000	-1.778153000
C	-0.940971000	-4.114051000	-2.007463000
H	-1.834505000	-4.429797000	-1.457473000
H	-0.119072000	-4.783705000	-1.740910000
H	-1.151394000	-4.271861000	-3.072658000
C	-1.760836000	-1.795534000	-2.325116000
H	-1.862048000	-1.987294000	-3.400513000
H	-1.576386000	-0.723440000	-2.188394000
H	-2.721649000	-2.036803000	-1.857039000

C	0.662538000	-2.266265000	-2.547043000
H	1.549382000	-2.809221000	-2.207527000
H	0.873142000	-1.195006000	-2.456650000
H	0.520650000	-2.495138000	-3.610575000
P	-0.357172000	2.155545000	-0.093132000
C	-2.183004000	2.295148000	-0.468157000
H	-2.629749000	3.195058000	-0.021718000
H	-2.344182000	2.354621000	-1.554309000
C	-0.215302000	3.187338000	1.496505000
C	-1.209677000	2.582839000	2.493569000
H	-1.098975000	3.095327000	3.456993000
H	-1.018398000	1.516263000	2.655283000
H	-2.252394000	2.695792000	2.179094000
C	-0.551532000	4.664479000	1.295771000
H	-0.567188000	5.158528000	2.275344000
H	-1.536378000	4.823922000	0.843013000
H	0.195054000	5.185245000	0.688892000
C	1.185840000	3.064145000	2.095029000
H	1.968623000	3.461320000	1.445780000
H	1.444484000	2.024086000	2.308126000
H	1.212349000	3.623777000	3.038473000
C	0.412204000	3.069072000	-1.572336000
C	0.513355000	2.033817000	-2.696027000
H	-0.456408000	1.574642000	-2.927287000
H	1.210425000	1.230084000	-2.438428000
H	0.873527000	2.521566000	-3.610571000
C	-0.446979000	4.237794000	-2.065646000
H	0.085748000	4.740223000	-2.882658000
H	-0.636374000	4.990083000	-1.296818000
H	-1.410626000	3.916099000	-2.471063000
C	1.810902000	3.581361000	-1.234587000
H	2.303413000	3.909923000	-2.159004000
H	2.429473000	2.810233000	-0.765883000

H	1.775728000	4.446937000	-0.565050000
H	1.568720000	-0.036378000	-0.625032000
B	2.258129000	0.019709000	0.501730000
O	3.120857000	-1.138652000	0.529823000
O	3.106035000	1.186431000	0.531551000
H	1.386300000	0.055948000	1.445800000
C	4.449795000	-0.682775000	0.793976000
C	4.422408000	0.748407000	0.185616000
C	5.422403000	1.720245000	0.780589000
H	6.452452000	1.377297000	0.629796000
H	5.325235000	2.701087000	0.302552000
H	5.255272000	1.856028000	1.852071000
C	4.659883000	-0.661799000	2.305161000
H	4.456840000	-1.658459000	2.710125000
H	5.684720000	-0.387504000	2.579200000
H	3.973751000	0.044623000	2.784188000
C	5.430216000	-1.637912000	0.139227000
H	5.390023000	-2.616410000	0.629639000
H	5.196495000	-1.787113000	-0.918278000
H	6.459598000	-1.269717000	0.218421000
C	4.550544000	0.716397000	-1.336506000
H	4.308344000	1.701962000	-1.747379000
H	5.565016000	0.458051000	-1.659548000
H	3.859054000	-0.012301000	-1.774596000

9. References

[1] Curado, N.; Maya, C; López-Serrano, J.; Rodríguez, A. *Chem. Commun.* **2014**, *50*, 15718-15721.

[2] Burés, J. Angew. Chem. Int. Ed. **2016**, *55*, 16084–16087.

[3] Zhao, Y.; Truhlar, D. G. J. Chem. Phys. **2006**, *125*, 194101–194118.

[4] Frisch, M. J.; Trucks, G. W.; Schlegel, H. B.; Scuseria, G. E.; Robb, M. A.; Cheeseman, J. R.; Scalmani, G.; Barone, V.; Mennucci, B.; Petersson, G. A.; Nakatsuji, H.; Caricato, M.; Li, X.; Hratchian, H. P.; Izmaylov, A. F.; Bloino, J.; Zheng, G.; Sonnenberg, J. L.; Hada, M.; Ehara, M.; Toyota, K.; Fukuda, R.; Hasegawa, J.; Ishida, M.; Nakajima, T.; Honda, Y.; Kitao, O.; Nakai, H.; Vreven, T.; Montgomery, J. A., Jr.; Peralta, J. E.; Ogliaro, F.; Bearpark, M.; Heyd, J. J.; Brothers, E.; Kudin, K. N.; Staroverov, V. N.; Keith, T.; Kobayashi, R.; Normand, J.; Raghavachari, K.; Rendell, A.; Burant, J. C.; Iyengar, S. S.; Tomasi, J.; Cossi, M.; Rega, N.; Millam, J. M.; Klene, M.; Knox, J. E.; Cross, J. B.; Bakken, V.; Adamo, C.; Jaramillo, J.; Gomperts, R.; Stratmann, R. E.; Yazyev, O.; Austin, A. J.; Cammi, R.; Pomelli, C.; Ochterski, J. W.; Martin, R. L.; Morokuma, K.; Zakrzewski, V. G.; Voth, G. A.; Salvador, P.; Dannenberg, J. J.; Dapprich, S.; Daniels, A. D.; Farkas, O.; Foresman, J. B.; Ortiz, J. V.; Cioslowski, J.; Fox, D. J. Gaussian 09, Revision E.01; Gaussian, Inc.: Wallingford, CT, 2013.

[5] Grimme, S.; Anthony, J.; Ehrlich, S.; Krieg, H. J. Chem. Phys. **2010**, *132*, 154104–154119.

[6] Marenich, S. A. V.; Cramer, C. J.; Truhlar, D. G. J. Phys. Chem. B **2009**, *113*, 6378–6396.

[7] (a) Hehre, W. J.; Ditchfield, R.; Pople, J. A. J. Chem. Phys. **1972**, *56*, 2257–2261. (b) Hariharan, P. C.; Pople, J. A. Theor. Chim. Acta **1973**, *28*, 213–222. (c) Francl, M. M.; Pietro, W. J.; Hehre, W. J.; Binkley, J. S.; Gordon, M. S.; DeFrees, D. J.; Pople, J. A. J. Chem. Phys. **1982**, *77*, 3654–3665.

[8] Andrae, D.; Häussermann, U.; Dolg, M.; Stoll, H.; Preuss, H. Theor. Chim. Acta **1990**, *77*, 123–141.

[9] Ehlers, A. W.; Boehme, M.; Dapprich, S.; Gobbi, A.; Hoellwarth, A.; Jonas, V.; Koehler, K. F.; Stegmann, R.; Veldkamp, A.; Frenking, G. Chem. Phys. Lett. **1993**, *208*, 111–114.

[10] (a) Weigend, F.; Ahlrichs, R. Phys. Chem. Chem. Phys. **2005**, *7*, 3297–3305. (b) Weigend, F. Phys. Chem. Chem. Phys. **2006**, *8*, 1057–1065.

[11] Grimme, S. Chem. Eur. J. **2012**, *18*, 9955–9964.

[12] Luchini, G.; Alegre-Requena, J. V.; Funes-Ardoiz, I.; Paton, R. S. F1000Research **2020**, *9*, 291. DOI: 10.12688/f1000research.22758.1.

[13] Chemcraft - Graphical software for visualization of quantum chemistry computations. https://www.chemcraftprog.com.

[14] (a) Adamo, C.; Barone, V. J. Chem. Phys. **1999**, *110*, 6158–6170. (b) Grimme, S.; Ehrlich, S.; Goerigk, L. J. Comp. Chem. **2011**, *32*, 1456–1465.
